# Anti-CD38 monoclonal antibody CM313 for systemic lupus erythematosus: a randomized, double-blind, placebo-controlled phase Ib/IIa trial

**DOI:** 10.1038/s41392-025-02487-2

**Published:** 2025-11-26

**Authors:** Jiuliang Zhao, Changsong Lin, Qibing Xie, Qiang Shu, Yang Cui, Hui Luo, Wenqiang Fan, Anbin Huang, Yi Zhao, Zili Fu, Changhao Xie, Huaxiang Wu, Niansheng Yang, Lan He, Ping Feng, Tiandong Zhang, Huan Zhou, Wei Liu, Qiaoyun Hou, Xihua Mao, Jing Sun, Bo Chen, Xiaofeng Zeng

**Affiliations:** 1 Department of Rheumatology and Clinical Immunology, Peking Union Medical College Hospital, Chinese Academy of Medical Sciences, Peking Union Medical College, Beijing, China; 2 National Clinical Research Center for Dermatologic and Immunologic Diseases (NCRC-DID), Ministry of Science & Technology, Beijing, China; 3 State Key Laboratory of Complex Severe and Rare Diseases, Peking Union Medical College Hospital, Beijing, China; 4 Key Laboratory of Rheumatology and Clinical Immunology, Ministry of Education, Beijing, China; 5 Department of Rheumatology, The First Affiliated Hospital of Guangzhou University of Chinese Medicine, Guangzhou, Guangdong China; 6 Department of Rheumatology and Immunology, West China Hospital of Sichuan University, Chengdu, Sichuan China; 7 Department of Rheumatology and Immunology, Qilu Hospital of Shandong University, Jinan, Shandong China; 8 Department of Rheumatology, Guangdong Provincial People’s Hospital, Guangzhou, Guangdong China; 9 Department of Rheumatology and Immunology, Xiangya Hospital, Central South University, Changsha, Hunan China; 10 Department of Rheumatology and Immunology, Xinxiang Central Hospital, The Fourth Clinical College of Xinxiang Medical University, Xinxiang, Henan China; 11 Department of Rheumatology and Immunology, Union Hospital, Tongji Medical College, Huazhong University of Science and Technology, Wuhan, Hubei China; 12 Department of Rheumatology & Allergy, Xuanwu Hospital Capital Medical University, Beijing, China; 13 Department of Rheumatology and Immunology, The First Hospital of Shanxi Medical University, Taiyuan, Shanxi China; 14 Department of Rheumatology and Immunology, The First Affiliated Hospital of Bengbu Medical University, Bengbu, Anhui China; 15 Department of Rheumatology, The Second Affiliated Hospital of Zhejiang University School of Medicine, Hangzhou, Zhejiang China; 16 Department of Rheumatology, The First Affiliated Hospital of Sun Yat-sen University, Guangzhou, Guangdong China; 17 Department of Rheumatology and Immunology, The First Affiliated Hospital of Xi’An Jiao Tong University, Xi’An, Shaanxi China; 18 Clinical Trial Center, National Medical Products Administration Key Laboratory for Clinical Research and Evaluation of Innovative Drugs, West China Hospital of Sichuan University, Chengdu, Sichuan China; 19 Drug Clinical Trial Instituti, Xinxiang Central Hospital, The Fourth Clinical College of Xinxiang Medical University, Xinxiang, Henan China; 20 Phase I Center, The First Affiliated Hospital of Bengbu Medical University, Bengbu, Anhui China; 21 Keymed Biosciences (Chengdu) Co., Ltd, Chengdu, Sichuan China

**Keywords:** Rheumatic diseases, Immunotherapy

## Abstract

CD38 is highly expressed on various immune cells, including long-lived plasma cells, making it a potential therapeutic target in autoimmune diseases. This phase Ib/IIa study aimed to explore the safety, pharmacokinetics, pharmacodynamics, and preliminary efficacy of CM313, an anti-CD38 antibody, in patients with systemic lupus erythematosus (SLE). Eligible patients were sequentially enrolled in four ascending dose groups (2, 4, 8, and 16 mg/kg) and randomized 4:1 to receive CM313 or placebo intravenously at days 1, 29, 36, 43, and 50. The primary endpoint was safety, and efficacy was exploratorily investigated. Between October 14, 2022, and March 7, 2024, 40 patients were enrolled, including 8 patients in each CM313 dose group and the pooled placebo group. Adverse events occurred in 90.6% and 62.5% of participants receiving CM313 and placebo, all of which were mild or moderate. Upper respiratory tract infection (87.5%/62.5% vs. 12.5%), urinary tract infection (12.5%/25.0% vs. 0), and herpes zoster (25.0%/0 vs. 0) were more frequent in CM313 8 and 16 mg/kg groups than the placebo group. The CM313 groups had greater reductions in anti-ds-DNA antibodies, immunoglobulin G (IgG), IgA, IgM, IgE, and greater increases in complement C3 and C4 compared with placebo. Systemic Lupus Erythematosus Responder Index-4 response rates were 33.3%, 40.0%, 62.5%, 71.4%, and 37.5% in CM313 2, 4, 8, 16 mg/kg, and placebo groups at day 57, respectively. CM313 showed a manageable safety profile in SLE patients at 2–16 mg/kg and encouraging clinical efficacy at 8 and 16 mg/kg. The results support further investigation of CM313 for treating SLE patients (ClinicalTrials.gov ID: NCT05465707).

## Introduction

Systemic lupus erythematosus (SLE) is a complex autoimmune disease characterized by inflammation and tissue damage in multiple organs.^1^ It impacts approximately 3.4 million people worldwide, and disproportionately affects women.^2^ The incidence and prevalence vary widely across regions, with the incidence in East Asia being second only to Central Europe.^2,3^ In urban China, SLE affected an estimated 0.4 million individuals in 2017.^4^ SLE is associated with a wide spectrum of clinical manifestations, leading to significantly impaired quality of life.^1,5^

The treatment of SLE is challenging, and long-term outcomes remain inadequately improved.^6,7^ The standard of care (SOC) for SLE can bring significant side effects, which hinder their long-term use.^8–10^ Biologics are recommended for patients who are refractory or intolerant to SOC therapies. Belimumab (anti-B-cell-activating factor) and anifrolumab (anti-interferon alpha receptor-1) have been approved globally for treating SLE patients.^5^ Rituximab (anti-CD20) is recommended as an off-label treatment option for refractory, organ-threatening SLE.^8^ However, there is still a substantial proportion of patients failing to respond to available therapies, underscoring the urgent need for more effective therapies. Numerous biological agents are under development for the treatment of SLE or lupus nephritis (LN). Obinutuzumab, a novel anti-CD20 monoclonal antibody, has demonstrated significant efficacy in combination with SOC for LN in a phase III trial.^11^

Autoantibodies play a pivotal role in the pathogenesis of SLE and are associated with disease activity.^12^ Depleting autoantibody-secreting plasma cells would serve as a promising strategy for treating SLE, but is challenging. Unlike short-lived plasma cells, long-lived plasma cells reside in the bone marrow and inflammatory tissue niches. They produce autoantibodies independent of B cell activation, driving persistent autoimmune inflammation. These cells do not respond to conventional immunosuppression or to current B cell-directed therapies due to a lack of standard B-cell markers.^13^

CD38 is a type II glycoprotein with ectoenzymatic functions, which is highly expressed on the surface of various immune cells, including plasma cells, natural killer (NK) cells, B cells, dendritic cells (DCs), T cells, hematopoietic stem cells, monocytes, and polymorphonuclear cells.^14^ Anti-CD38 monoclonal antibody daratumumab, which has been approved for multiple myeloma (MM), effectively depletes malignant plasma cells in the bone marrow of MM patients.^15^ Anti-CD38 antibodies also demonstrated substantial ex vivo depletion of plasma cells/plasmablasts in PBMC (daratumumab and mezagitamab) and long-lived plasma cells in the bone marrow (mezagitamab) from patients with SLE.^16,17^ However, clinical evidence for the effectiveness of targeting CD38 for SLE remains limited. Preliminary efficacy of daratumumab has only been reported in sporadic cases of refractory SLE^18,19^ and lupus nephritis^20^ as well as in a single-arm investigator-initiated study with a small sample size of 10.^21^ Nevertheless, mezagitamab did not show observable differences in disease activity improvement compared with placebo after 12 weeks of treatment in a phase Ib trial.^22^

CM313 is a novel anti-CD38 monoclonal antibody with a unique complementarity-determining region sequence that induces complement-dependent cytotoxicity (CDC), antibody-dependent cellular cytotoxicity (ADCC), and antibody-dependent cellular phagocytosis (ADCP). Preclinical studies revealed potent pathogenic CD38+ cell-killing activity comparable with daratumumab.^23^ CM313 monotherapy in patients with relapsed/refractory MM (RRMM) showed a tolerable and manageable safety profile as well as promising efficacy.^24^ In patients with immune thrombocytopenia (ITP), CM313 maintained long-term efficacy with mainly low-grade toxic effects, supporting the therapeutic potential of CM313 in autoimmune diseases.^25^

This study aimed to explore the safety, tolerability, pharmacokinetics, pharmacodynamics, immunogenicity, and preliminary efficacy of CM313 injection in adult patients with SLE.

## Results

### Patient characteristics

**Fig. 1:**
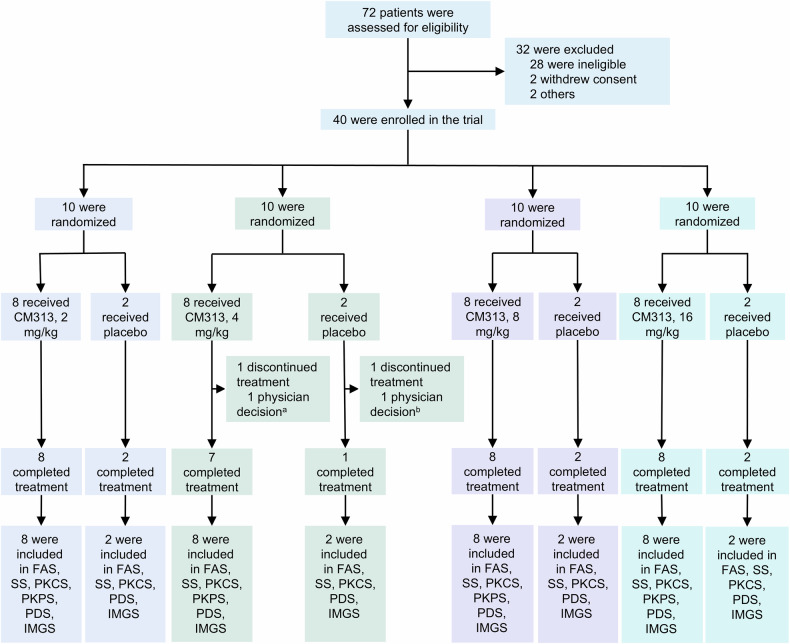
Flow diagram of the study. ^a^The patient had worse headaches than before enrollment, raising safety concerns. ^b^The patient had to be vaccinated with rabies vaccine, and the study treatment had to be delayed for a long time. The patient would benefit little from restarting treatment as judged by the investigator. FAS full analysis set, SS safety set, PKCS pharmacokinetic concentration set, PKPS pharmacokinetic parameter set, PDS pharmacodynamic set, IMGS immunogenicity set

Between October 14, 2022, and March 7, 2024, a total of 72 patients were screened, and 40 were enrolled. In each dose cohort, ten participants were randomized 4:1 to receive either CM313 or a placebo. One participant receiving CM313 4 mg/kg and one participant receiving placebo had discontinued treatment due to physician's decision (Fig. 1).

The demographic and disease characteristics of the participants at baseline are presented in Table 1. The mean (± standard deviation [SD]) age of the participants was 33.6 ± 12.0 years, and 87.5% (35/40) of patients were female. Participants had a median SLE duration of 8.11 years (range, 0.2 to 20.9). The mean (±SD) baseline Safety of Estrogens in Lupus Erythematosus National Assessment-Systemic Lupus Erythematosus Disease Activity Index (SELENA-SLEDAI) scores were 3.3 ± 1.8, 4.5 ± 3.1, 8.3 ± 1.7, and 6.5 ± 3.0 in the CM313 2 mg/kg, 4 mg/kg, 8 mg/kg, and 16 mg/kg groups and 8.5 ± 3.8 in the placebo group. All participants were taking SOC for SLE at baseline, including corticosteroids for systemic use (100%), immunosuppressants (97.5%), and anti-inflammatory and anti-rheumatic products (5.0%). Most patients (72.5%) were receiving an average daily dose of prednisone (or equivalent) of ≤10 mg at baseline.

**Table 1:** Baseline demographic and disease characteristics of the patients

Characteristic	CM313, 2 mg/kg (*n* = 8)	CM313, 4 mg/kg (*n* = 8)	CM313, 8 mg/kg (*n* = 8)	CM313, 16 mg/kg (*n* = 8)	Placebo (*n* = 8)
Age, years	32.1 (10.1)	35.0 (15.6)	33.8 (9.4)	38.3 (14.2)	28.9 (10.6)
Sex^*^					
Male	1 (12.5)	1 (12.5)	3 (37.5)	0	0
Female	7 (87.5)	7 (87.5)	5 (62.5)	8 (100)	8 (100)
Body mass index, kg/m^2^	22.9 (3.4)	20.8 (2.9)	20.6 (3.0)	24.0 (5.6)	23.2 (7.4)
Duration of SLE, years	8.3 (0.2–16.6)	6.9 (1.1–14.6)	10.6 (0.3–17.3)	5.4 (1.4–14.4)	7.6 (3.0–20.9)
SELENA-SLEDAI score	3.3 (1.8)	4.5 (3.1)	8.3 (1.7)	6.5 (3.0)	8.5 (3.8)
PGA score	1.09 (0.43)	1.09 (0.48)	1.81 (0.39)	1.78 (0.37)	1.35 (0.45)
BILAG-2004 score ≥1 A or ≥2 B^†^	1 (12.5)	1 (12.5)	2 (25.0)	1 (12.5)	1 (12.5)
Anti-ds-DNA antibody, IU/mL	58.5 (17–260)	130.5 (10–216)	198 (10–613)	127 (18–664)	137.5 (30–733)
Positive	2 (25.0)	4 (50.0)	6 (75.0)	4 (50.0)	5 (62.5)
C3 concentration, g/L	0.774 (0.148)	0.728 (0.195)	0.795 (0.194)	0.780 (0.172)	0.866 (0.212)
C4 concentration, g/L	0.120 (0.066)	0.153 (0.038)	0.138 (0.066)	0.128 (0.058)	0.170 (0.098)
Concomitant SOC for SLE	8 (100)	8 (100)	8 (100)	8 (100)	8 (100)
Corticosteroids for systemic use	8 (100)	8 (100)	8 (100)	8 (100)	8 (100)
Immunosuppressants	8 (100)	7 (87.5)	8 (100)	8 (100)	8 (100)
Anti-inflammatory and anti-rheumatic products	0	1 (12.5)	0	1 (12.5)	0
Average daily dose of prednisone (or equivalent), mg	6.3 (2.5–17.5)	5.0 (1.1–15.0)	10.0 (7.5–20.0)	8.8 (2.5–30.0)	10.0 (5.0–30.0)
≤10 mg	5 (62.5)	7 (87.5)	5 (62.5)	5 (62.5)	7 (87.5)
>10 mg	3 (37.5)	1 (12.5)	3 (37.5)	3 (37.5)	1 (12.5)

Data are mean (SD), *n* (%), or median (range)

^*^Sex was self-reported by the participants

^†^BILAG-2004 1A score means at least one severe symptom in one of the nine domains (constitutional, mucocutaneous, neuropsychiatric, musculoskeletal, cardiorespiratory, gastrointestinal, ophthalmic, renal, and hematological); BILAG 2B score means at least one moderate symptom in two of the nine domains. SLE=systemic lupus erythematosus. SELENA-SLEDAI=Safety of Estrogens in Lupus Erythematosus National Assessment-Systemic Lupus Erythematosus Disease Activity Index

*PGA* Physician Global Assessment, *BILAG* British Isles Lupus Assessment Group, *ds-DNA* double-stranded deoxyribonucleic acid, *SOC* standard of care

### Safety

Adverse events occurred in 90.6% (29 of 32) of participants receiving CM313 and in 62.5% (5 of 8) of participants receiving placebo (Table 2). All the adverse events were mild or moderate in severity. No serious adverse events or adverse events leading to death or treatment discontinuation occurred in any group. The most frequent adverse events in all CM313-treated patients were upper respiratory tract infection (17 [53.1%]), infusion-related reaction (IRR; 14 [43.8%]), and lymphocyte count decreased (5 [15.6%]). Upper respiratory tract infection (87.5%/62.5% vs. 12.5%), urinary tract infection (12.5%/25.0% vs. 0), and herpes zoster (25.0%/0 vs. 0) were more frequent in CM313 8 and 16 mg/kg groups than the placebo group. A total of 18 IRRs were reported in 5 (62.5%), 3 (37.5%), 4 (50.0%), and 2 (25.0%) patients in the CM313 2 mg/kg, 4 mg/kg, 8 mg/kg, and 16 mg/kg groups, respectively. IRRs occurred in 37.5% (12 of 32) of patients during the first infusion and in 12.5% (4 of 32) during subsequent infusions.

**Table 2:** Adverse events

Event	CM313, 2 mg/kg (*n* = 8)	CM313, 4 mg/kg (*n* = 8)	CM313, 8 mg/kg (*n* = 8)	CM313, 16 mg/kg (*n* = 8)	Placebo (*n* = 8)
Any adverse event	8 (100)	7 (87.5)	8 (100)	6 (75.0)	5 (62.5)
Drug-related	7 (87.5)	3 (37.5)	7 (87.5)	5 (62.5)	3 (37.5)
Severe adverse event	0	0	0	0	0
Serious adverse event	0	0	0	0	0
Adverse event leading to death	0	0	0	0	0
Adverse event leading to treatment discontinuation	0	0	0	0	0
Event observed in ≥2 participants in any treatment group^*^					
Upper respiratory tract infection	3 (37.5)	2 (25.0)	7 (87.5)	5 (62.5)	1 (12.5)
IRR	5 (62.5)	3 (37.5)	4 (50.0)	2 (25.0)	0
Lymphocyte count decreased	2 (25.0)	2 (25.0)	0	1 (12.5)	0
Urinary tract infection	0	1 (12.5)	1 (12.5)	2 (25.0)	0
Cough	3 (37.5)	1 (12.5)	0	0	1 (12.5)
Globulins decreased	2 (25.0)	1 (12.5)	0	0	1 (12.5)
Dizziness	2 (25.0)	1 (12.5)	0	0	1 (12.5)
Herpes zoster	0	0	2 (25.0)	0	0
Vaginal infection	2 (25.0)	0	0	0	0
Positive SARS-CoV-2 detection	2 (25.0)	0	0	0	0
Alpha-hydroxybutyrate dehydrogenase increased	2 (25.0)	0	0	0	0
White blood cell count increased	1 (12.5)	1 (12.5)	0	0	2 (25.0)
Blood triglycerides increased	2 (25.0)	0	0	0	1 (12.5)
Neutrophil count increased	1 (12.5)	1 (12.5)	0	0	2 (25.0)
Protein total decreased	2 (25.0)	0	0	0	0

Data are *n* (%)

^*^Coded according to the latest version of the Medical Dictionary for Regulatory Activities. Adverse events were those that occurred or worsened after the receipt of the first dose of CM313 or placebo until completion of the study or early withdrawal

*IRR* infusion related reaction

### Pharmacokinetics, pharmacodynamics, and immunogenicity

In the dose range of 2 mg/kg to 16 mg/kg, exposure to CM313, which was measured as the maximum concentration (C_max_) and area under the serum concentration-time curve (AUC), increased with higher doses in a greater than dose-proportional manner after the first and last infusions (Supplementary Fig. 1; Supplementary Table 1). The median time to maximum concentration (T_max_) ranged from 6.60 to 11.01 hours after the first dose of CM313 in different dose groups. Clearance decreased with increasing doses and multiple doses. The mean clearance decreased from 41.3 mL/h in the 2 mg/kg cohort to 10.6 mL/h in the 16 mg/kg cohort after the first infusion, and from 13.5 mL/h in the 2 mg/kg cohort to 8.5 mL/h in the 16 mg/kg cohort after the last infusion. After the last infusion, mean elimination half-life (t_1/2z_) ranged from 110 to 316 h in different dose groups. Mean accumulation index (R_ac_)_Cmax_ ranged from 1.85 to 2.18, and mean R_ac_,_AUC_ ranged from 2.59 to 3.43 in different dose groups after the last infusion, indicating accumulation of CM313 after multiple doses (Supplementary Table 2).

**Fig. 2:**
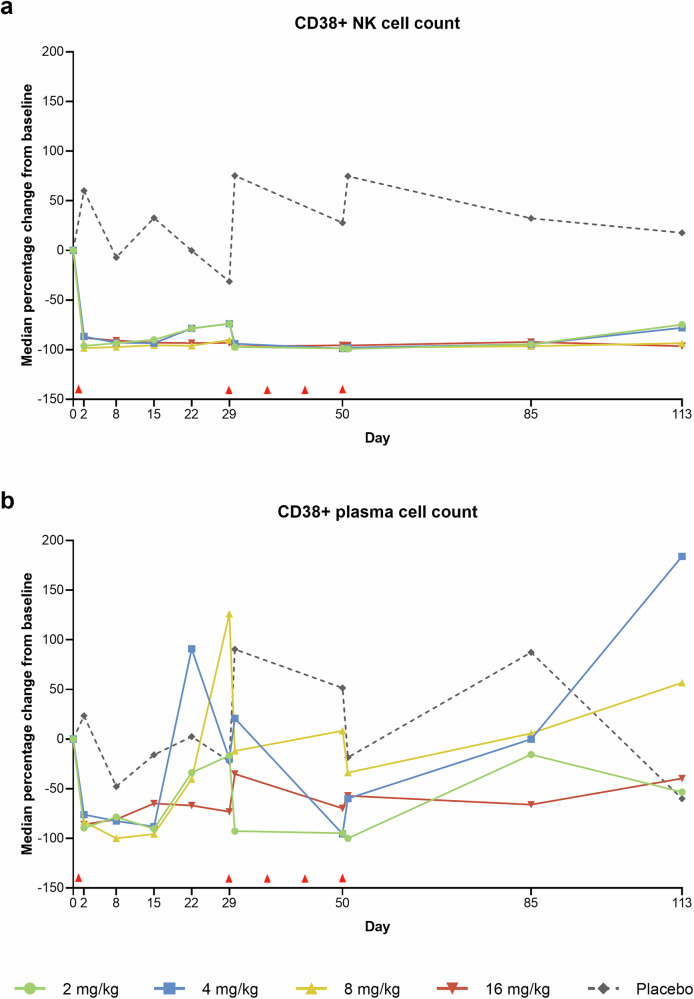
Median percentage change from baseline in CD38 + NK cell counts and plasma cell counts. **a** CD38+ natural killer (NK) cell count. **b** CD38+ plasma cell count. Red triangle marks indicate the time points of CM313 or placebo administrations. BL baseline

In patients receiving CM313, substantial reductions in nature killer (NK) cell counts (CD3-CD16 + CD56 + CD38+) in peripheral blood were observed after drug administration. CD38 + NK cell counts demonstrated reductions of 96.2%, 86.8%, 98.4%, and 88.3% in CM313 2 mg/kg, 4 mg/kg, 8 mg/kg, and 16 mg/kg groups, respectively, at 24 hours after the end of the first infusion, compared to a 60.0% increase in the placebo group. Reductions in the CM313 groups sustained even after the treatment discontinuation until the end of the study (Fig. 2a). The plasma cell (CD3-CD16-CD56-CD27 + CD38+) counts decreased by 89.6%, 76.1%, 83.3%, and 86.0% from baseline in CM313 2 mg/kg, 4 mg/kg, 8 mg/kg, and 16 mg/kg groups, respectively, versus a 23.5% increase in the placebo group, 24 hours post first infusion. However, the counts rebounded before the second infusion on day 29 and decreased following the subsequent infusions (Fig. 2b). Reductions in CD38 + B cell, CD38 + T cell, and CD38+ plasmacytoid dendritic cell (pDC) counts were also observed following CM313 administrations, except for a rebound in T cell counts in the 8 mg/kg group (Supplementary Fig. 2).

Treatment-related anti-drug antibody (ADA) was observed in one patient (3.1%, 1 of 32) in all CM313-treated patients. No effect of treatment-related ADAs on pharmacokinetics and safety of CM313 was observed.

### Efficacy

**Fig. 3:**
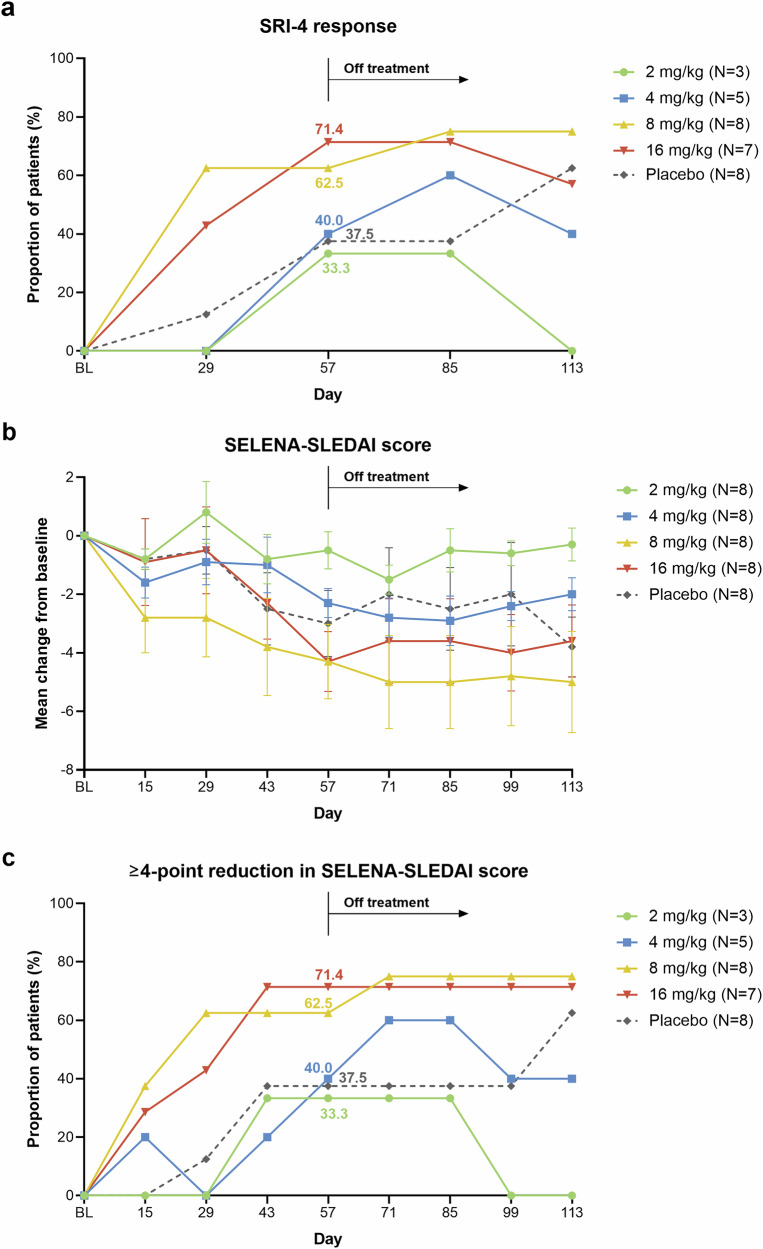
SRI-4 response rate and improvement in SELENA-SLEDAI score. **a** The proportion of patients achieving a Systemic Lupus Erythematosus Responder Index (SRI)-4 response, defined as a ≥ 4-point reduction from baseline (BL) in the Safety of Estrogens in Lupus Erythematosus National Assessment-Systemic Lupus Erythematosus Disease Activity Index (SELENA-SLEDAI) score, no new disease activity as measured by one A score or two B scores on the British Isles Lupus Assessment Group 2004 index (BILAG-2004), and a < 0.3-point increase in the Physician’s Global Assessment (PGA) score. Missing data was imputed by the last observation carried forward method. N indicates the number of evaluable patients, defined as those with a SELENA-SLEDAI score ≥4, not all A scores in all organ domains on BILAG-2004, and a PGA score ≤2.7 at baseline. **b** Mean change from baseline in SELENA-SLEDAI score. Error bars denote standard errors. All patients enrolled were evaluable. **c** The proportion of patients achieving a ≥ 4-point reduction in SELENA-SLEDAI score from baseline. *N* indicates the number of evaluable patients, defined as those with a SELENA-SLEDAI score ≥4 at baseline

Among evaluable patients, CM313 8 mg/kg and 16 mg/kg groups achieved remarkably higher Systemic Lupus Erythematosus Responder Index (SRI)-4 response rates than the placebo group after the first infusion (day 29). At the end of the treatment period (day 57), the SRI-4 response rates were 33.3%, 40.0%, 62.5%, and 71.4% in the CM313 2 mg/kg, 4 mg/kg, 8 mg/kg, and 16 mg/kg groups, respectively, compared to 37.5% in the placebo group (Fig. 3a). The CM313 8 mg/kg and 16 mg/kg groups also achieved numerically greater reductions from baseline in SELENA-SLEDAI score compared with the placebo group (Fig. 3b). The proportions of patients achieving a ≥ 4-point reduction in the SELENA-SLEDAI score showed a similar trend with SRI-4 response (Fig. 3c). These response rates continued to improve and were maintained up to day 85.

The CM313 groups achieved greater change from baseline in Physician Global Assessment (PGA) score compared with the placebo group (Supplementary Fig. 3a). In addition, the proportions of patients achieving an improved PGA score (Supplementary Fig. 3b) and an improved British Isles Lupus Assessment Group 2004 index (BILAG-2004) score (Supplementary Fig. 4) at day 57 were numerically higher in the CM313 groups compared with the placebo group.

The median time to the first SLE flare from randomization was 98.0 days in the CM313 4 mg/kg group and not reached in other groups. The changes in average daily prednisone (or equivalent) dose during the trial are detailed in Supplementary Table 3.

**Fig. 4:**
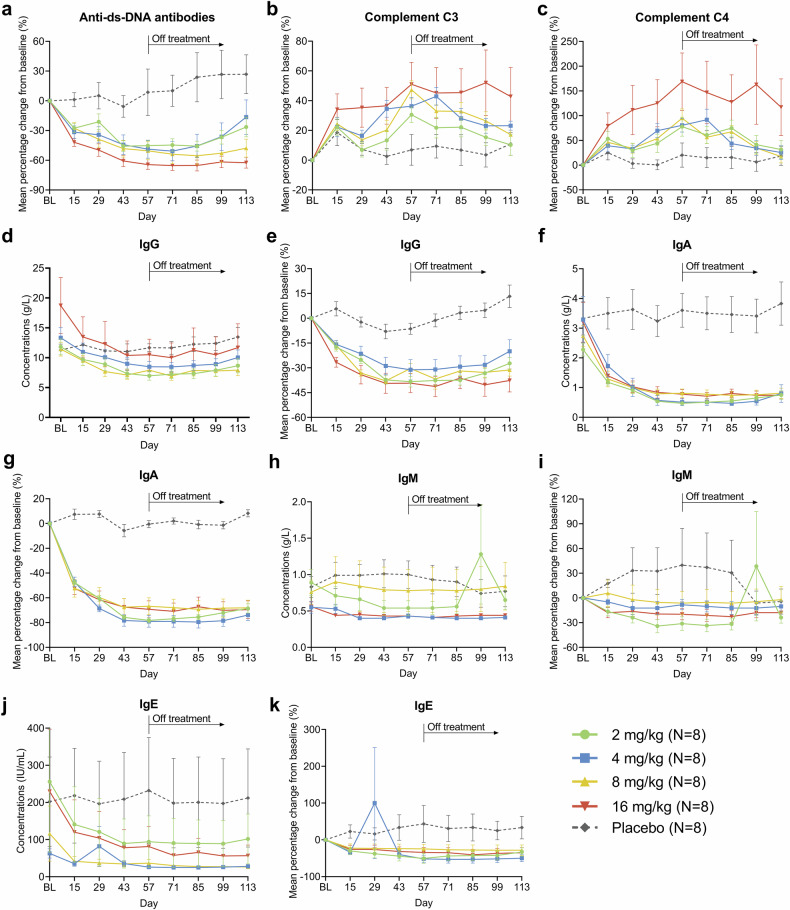
Mean percentage changes from baseline in immune biomarkers and concentrations of immunoglobulins. **a**–**c** Mean percentage changes in anti-double-stranded deoxyribonucleic acid (ds-DNA) antibodies, complement C3, and complement C4. **d**, **e** Concentrations and mean percentage changes in IgG. **f**, **g** Concentrations and mean percentage changes in IgA. **h**, **i** Concentrations and mean percentage changes in IgM. **j**, **k** Concentrations and mean percentage changes in IgE. Error bars denote standard errors. BL baseline, Ig immunoglobulin

The percentage changes in serological biomarkers and absolute levels of immunoglobulins are presented in Fig. 4. The CM313 groups achieved greater reductions from baseline in anti-ds-DNA antibodies compared with placebo, which appeared dose dependent and were sustained until day 85. Among patients with positive anti-ds-DNA antibodies at baseline, 50.0% (2/4), 66.7% (4/6), and 50.0% (2/4) in CM313 4 mg/kg, 8 mg/kg, and 16 mg/kg groups, respectively, converted to negative during the treatment and follow-up period. Furthermore, the CM313 groups had greater increases in complement proteins (C3 and C4) and greater reductions in immunoglobulins (IgG, IgA, IgM, and IgE) compared with placebo. These changes were sustained throughout the treatment period and the off-treatment period.

## Discussion

The heterogeneity in clinical presentation and pathophysiology of SLE makes the disease management and drug development challenging. Despite the recent approval of new therapies, there remains an urgent need for advanced therapies given the modest response rates.^26–28^ Unfortunately, a number of phase III trials in SLE failed to meet the primary endpoints. Besides the heterogeneity of disease, another important reason is that participants often received aggressive background medications that could contribute to high response rates in placebo arms, and significant benefits were not observed with the addition of investigational drugs.^29^ CM313, a novel humanized IgG monoclonal anti-CD38 antibody, at a dose of 2 mg/kg to 16 mg/kg in combination SOC, showed a manageable safety profile in patients with SLE and the doses of 8 mg/kg and 16 mg/kg demonstrated preliminary efficacy with higher response rates for SRI-4 versus SOC alone at day 57 (62.5% and 71.4% vs. 37.5%). The CM313 groups also showed substantial responses in serological activity, including reduced anti-ds-DNA antibodies, reduced immunoglobulins, and increased complement C3 and C4.

Daratumumab has been reported to induce substantial clinical responses in two patients with life-threatening SLE.^18^ In an open label investigator-initiated study, daratumumab (1800 mg, subcutaneously, once a week [QW] for 8 doses) resulted in a median reduction of SLEDAI-2K from 12 to 4 and an SRI-4 response of 100% in ten patients with moderate-to-severe SLE at week 12.^21^ However, in a recent randomized controlled phase Ib study, another anti-CD38 monoclonal antibody mezagitamab (45 mg, 90 mg, 135 mg, Q3W) yielded comparable reductions versus placebo in Cutaneous Lupus Erythematosus Disease Area and Severity Index and SLEDAI-2K at week 12.^22^ The authors attributed the unsuccess to the dosing regimen which was not optimal to produce maximal pharmacological effects.

The dose selection of CM313 in this study was based on previous clinical trials of CM313 in RRMM and other CD38 monoclonal antibodies. In a phase I trial, CM313 2 mg/kg was well tolerated by RRMM patients and showed preliminary efficacy.^24^ Therefore, 2 mg/kg was selected as the starting dose in this study. Daratumumab 16 mg/kg, in the above-mentioned cases, report of two patients with life-threatening SLE, showed tolerability with remarkable clinical response.^18^ Combining the favorable safety profile of the 8 mg/kg group, 16 mg/kg dose group was initiated in this study. CM313 was accumulated after multiple doses, and the elimination of CM313 decreased with increasing dose and multiple administrations. Treatment-related ADA was observed in only one patient after the last infusion.

In vitro study has showed the depletion of plasma cells by mezagitamab is mainly mediated by ADCC, which is dependent on the effector cell NK cells.^17^ Since highly expressing CD38, the level of NK cells also dramatically decreases with the treatment of CD38 antibody. In contrast, CM313 showed a high CDC activity in vitro, suggesting that pathological cells could be eliminated despite of low levels of effector cells.^23^ In this study, CM313 8 mg/kg and 16 mg/kg groups demonstrated sustained depletion of CD38 + NK cells. NK cell counts showed slight rebounds in CM313 2 mg/kg and 4 mg/kg dose groups between two and four weeks post-first dose but declined and were sustained shortly after the second dose. Plasma cell counts exhibited fluctuations, with only transient depletion post-first dose in all CM313 groups. However, following four additional QW doses, the CM313 groups generally showed greater reductions from baseline than the placebo group. Following the first administration, CM313 rapidly induced effector functions such as ADCC and ADCP, leading to a significant decrease in peripheral plasma cell levels. The 28-day interval between the first and second doses (the DLT observation period) allowed plasma concentrations to decline and plasma cell levels to gradually rebound. By the time of the second administration, plasma cells had returned to or exceeded baseline levels. Plasma cells are normally scarce in peripheral blood. Subsequent multiple dosing may also exert effects on the immune microenvironment, resulting in a relatively smaller reduction in plasma cells compared to that observed after the initial dose. Overall, however, plasma cell levels maintained a downward trend.

The incidences of adverse events were generally similar across different CM313 dose groups in this study. The most common adverse events were upper respiratory tract infection, IRR, and lymphocyte count decreased, consistent with previous report of CM313 in ITP patients (IRR and upper respiratory tract infection).^25^ Although infections were more frequently reported in CM313 8 mg/kg and 16 mg/kg groups than in the placebo group, all these events were mild to moderate, and all patients recovered completely. The incidence of IRRs was 43.8% in this study, comparable to 32% in patients with ITP^25^ and lower than 59% in patients with RRMM^24^ receiving CM313 intravenously. Most of the IRRs occurred during the first two infusions, and were transient and resolved spontaneously or with treatment on the same day, with a median duration of 1.50 hours in all CM313-treated patients. Moreover, although IgA levels decreased in the CM313 groups and were numerically lower than those in the placebo group, the clinical relevance of this reduction appeared limited. The incidence of globulins decreased was comparable between all CM313-treated (9.4%, 3/32) and placebo-treated (12.5%, 1/8) patients. No serious adverse event or adverse event leading to treatment discontinuation were reported, indicating a manageable safety profile of CM313 up to 16 mg/kg.

Our study was an early-phase trial demonstrating preliminary efficacy of CM313 in a limited sample size of patients with SLE. Patients included in this study had mild-to-moderate disease. As the participants were sequentially enrolled rather than randomized to different dose groups, baseline SELENA-SLEDAI scores showed some variability and were lower in CM313 2 mg/kg and 4 mg/kg groups. Consequently, only half of the patients in these two dose groups (8/16) were evaluable for SRI-4 response. Moreover, anti-CD38 therapies target long-lived plasma cells, suggesting potential long-term effects. The relatively short treatment and follow-up periods in this study may not be sufficient to fully evaluate the efficacy and safety of CM313. A phase II trial is underway to evaluate the efficacy and safety of CM313 subcutaneous injections in a larger group of SLE patients, with a 24-week treatment period and a 28-week follow-up period (NCT06791772).

In conclusion, CM313 was well tolerated in SLE patients at doses of 2–16 mg/kg and showed encouraging pharmacodynamic effects and clinical efficacy at doses of 8 and 16 mg/kg QW. The results support further investigation of CM313 as a new treatment option for SLE patients.

## Methods

### Study design

This was a randomized, double-blind, placebo-controlled, multiple-ascending-dose, phase Ib/IIa clinical trial conducted at 18 centers in China. The study consisted of a screening period of up to 4 weeks, an 8-week treatment period, and an 8-week follow-up period (Supplementary Fig. 5). The treatment period included a single-dose phase (Day 1 to Day 28) and a multiple-dose phase (Day 29 to Day 57). This trial was conducted in accordance with the Declaration of Helsinki and Good Clinical Practice guidelines. The study protocol and amendments were approved by the ethics committees of each participating center. The study was registered with clinicaltrials.gov (https://clinicaltrials.gov/study/NCT05465707?term=NCT05465707&rank=1, registered on July 19, 2022, identifier: NCT05465707). Written informed consent was obtained from all participants before enrollment.

### Participants

Eligible participants were 18-65 years old, diagnosed with SLE according to the 2019 European League Against Rheumatism (EULAR)/American College of Rheumatology (ACR) classification criteria for SLE,^30^ with a SELENA-SLEDAI score between 0 to 12 (inclusive) and seropositive for antinuclear antibody (ANA) at screening, and was receiving a stable SOC regimen, which refers to the use of any of the following (alone or in combination): corticosteroids, antimalarials, nonsteroidal anti-inflammatory drugs (NSAIDs), immunosuppressive drugs or immunomodulators, for at least 30 days before the first dose. Sex was self-reported by the participants.

Key exclusion criteria were renal disorders such as severe lupus nephritis (defined as urine protein >6 g/24 hours or serum creatinine >2.5 mg/dL or 221 μmol/L), active nephritis requiring treatment with drugs prohibited by the protocol, or conditions requiring hemodialysis, or treatment with prednisone ≥100 mg/d or equivalent glucocorticoids for ≥14 days, central nervous system diseases, abnormal liver or renal function or hematology, or history of clinically significant diseases. Full inclusion and exclusion criteria are detailed in Supplementary Methods.

### Randomization and blinding

Randomization was conducted using an interactive web response randomization system (IWRS) and the randomization numbers were generated using SAS v9.4 by the randomization statistician. CM313 and placebo were packaged in vials that were indistinguishable by the unaided eye. Blinding personnel who did not participate in any other work related to this clinical trial conducted the drug blinding and labeling. Grouping was blinded to the participants and investigators.

### Procedures

Eligible patients with SLE were sequentially enrolled in four cohorts receiving ascending doses (2 mg/kg, 4 mg/kg, 8 mg/kg, and 16 mg/kg). Each cohort included 10 participants who were randomized in a 4:1 ratio to receive intravenous infusions of the corresponding dose of CM313 or placebo. Patients were observed for 28 days after the first dose (Day 1). After the safety and tolerability were assessed as acceptable by the investigator, patients entered the multiple-dose phase and received four consecutive doses QW. To reduce the risk of IRRs, pre- and post-infusion medications were administered, including corticosteroids, acetaminophen, and antihistamines. The SOC regimens were allowed to be adjusted by the investigators as needed per the clinical conditions of the patients. Details of the SOC regimens and pre- and post-infusion medications were provided in Supplementary Methods.

### Outcomes

The primary objective was safety and tolerability. Safety assessments included monitoring of adverse events, laboratory tests, physical examination, vital signs, and 12-lead electrocardiogram (ECG). Adverse events were coded using the Medical Dictionary for Regulatory Activities and classified by system organ classes and preferred terms.

Secondary objectives included pharmacokinetics, pharmacodynamics, and immunogenicity. Pharmacokinetic endpoints were analyzed after the first and last infusions, including T_max_, C_max_), AUC_tau_, AUC_0-t_, AUC_0-∞_, t_1/2z_, apparent clearance (CL), apparent volume of distribution (V_z_), mean residence time (MRT_0-t_), and R_ac_. Pharmacodynamic measures included percentage changes in different types of immune cells from peripheral blood. Immunogenicity endpoint was ADAs. Experimental details of pharmacodynamic assessments were presented in Supplementary Methods.

Preliminary efficacy was also exploratorily investigated. Efficacy outcomes included changes and percent changes from baseline in SELENA-SLEDAI score and PGA score, proportions of patients achieving the following responses: a ≥ 4-point reduction from baseline in SELENA-SLEDAI score, improved PGA score from baseline, improved BILAG-2004 score from baseline, and an SRI-4 response; time from randomization to the first SLE flare; and change from baseline in prednisone (or equivalent) dose. The SRI-4 response was defined as ≥4-point reduction in the SELENA-SLEDAI score from baseline, no new disease activity as measured by ≥1 A score or ≥2 B scores on BILAG-2004, and <0.3-point increase in the PGA score compared with baseline. Efficacy outcomes also included serologic response of biomarkers encompassing anti-double-stranded deoxyribonucleic acid (ds-DNA) antibodies, IgG, IgA, IgM, IgE, complement C3, and complement C4.

Details of the assessments of the endpoints are described in Supplementary Methods.

### Statistical analysis

The safety set included all subjects who received at least one dose of the investigational medicinal product (IMP) and was used for safety analysis. The full analysis set included all randomized subjects who received at least one dose of the IMP and was used for demographic, baseline data, and efficacy analyses. The pharmacokinetic concentration sets for the first and last doses, pharmacokinetic parameter sets for the first and last doses, pharmacodynamic set, and immunogenicity set included all patients who received the corresponding dose of the IMP and had at least one corresponding valid result.

Descriptive statistics were used for all endpoints. The Kaplan-Meier method was used to estimate the first-flare rate of SLE at different time points in each group and the median time to first-flare of SLE, along with their 95% confidence intervals (Brookmeyer-Crowley method). Missing data for SELENA-SLEDAI, PGA, and BILAG-2004 scores were imputed by the last observation carried forward (LOCF) method; the other outcomes were analyzed as observed. The pharmacokinetic parameters for the first and last doses were calculated using a non-compartmental model using Phoenix WinNonlin version 8.3.

All statistical analyses were performed using the statistical software SAS v9.4.

## Supplementary information

Supplementary materials

Clinical Study Protocol

## Data Availability

The data supporting the findings of this trial are available within the manuscript and Supplementary materials. All requests for further data sharing will be reviewed by the leading clinical center, Peking Union Medical College Hospital, and the trial sponsor, Keymed Biosciences, to verify whether the request is subject to any intellectual property or confidentiality obligations. A signed data access agreement with the sponsor is required before accessing shared data. Individual participant data will not be shared. The study protocol is available in the Supplementary Information. We note that CM313 is still under clinical development. Section 3.3 (“Rationale for Dose Selection”) of the protocol includes early research data on CM313, which is deemed relatively confidential and closely linked to other ongoing studies of CM313. Accordingly, the relevant content in Section 3.3 (pages 39–40) has been redacted.
