# Supplementary material for: Anti-CD38 monoclonal antibody CM313 for systemic lupus erythematosus: a randomized, double-blind, placebo-controlled phase Ib/IIa trial
Source: Signal Transduct Target Ther. 2025 Nov 26;10:383. doi: 10.1038/s41392-025-02487-2 (PMC12647876; doi:10.1038/s41392-025-02487-2)
Supplement: Supplementary file 1 — Supplementary materials [file 41392_2025_2487_MOESM1_ESM.docx]

Supplementary Materials for

Anti-CD38 monoclonal antibody CM313 for systemic lupus erythematosus: a randomized, double-blind, placebo-controlled phase Ib/IIa trial

Jiuliang Zhao, Changsong Lin, Qibing Xie, Qiang Shu, Yang Cui, Hui Luo, Wenqiang Fan, Anbin Huang, Yi Zhao, Zili Fu, Changhao Xie, Huaxiang Wu, Niansheng Yang, Lan He, Ping Feng, Tiandong Zhang, Huan Zhou, Wei Liu, Qiaoyun Hou, Xihua Mao, Jing Sun, Bo Chen, Xiaofeng Zeng

Correspondence to: zengxfpumc@163.com

**This PDF file includes:**

List of Investigators

Supplementary Methods

Figures. S1 to S5

Tables S1 to S3

List of Investigators

| **Site** | **Principal Investigator** | **Site Name** |
| --- | --- | --- |
| 01 | Xiaofeng Zeng, Jiuliang Zhao | Peking Union Medical College Hospital, Chinese Academy of Medical Sciences, Peking Union Medical College |
| 02 | Yi Zhao | Xuanwu Hospital Capital Medical University |
| 03 | Yuewu Lu | Beijing Chao-Yang Hospital, Capital Medical University |
| 04 | Jialin Teng | Ruijing Hospital, School of Medicine, Shanghai Jiaotong University |
| 05 | Niansheng Yang | The First Affiliated Hospital of Sun Yat-sen University |
| 06 | Anbin Huang | Union Hospital, Tongji Medical College, Huazhong University of Science and Technology |
| 07 | Shengyun Liu | The First Affiliated Hospital of Zhengzhou University |
| 08 | Lan He | The First Affiliated Hospital of Xi’an Jiaotong University |
| 09 | Hui Luo | Xiangya Hospital, Central South University |
| 10 | Qiang Shu | Qilu Hospital of Shandong University |
| 11 | Qibing Xie, Ping Feng | West China Hospital of Sichuan University |
| 12 | Huaxiang Wu, Zourong Ruan | The Second Affiliated Hospital of Zhejiang University School of Medicine |
| 13 | Yang Cui | Guangdong Provincial People’s Hospital |
| 14 | Zili Fu | The First Hospital of Shanxi Medical University |
| 15 | Changhao Xie, Huan Zhou | The First Affiliated Hospital of Bengbu Medical University |
| 16 | Li Cui | Beijing Tongren Hospital, Capital Medical University |
| 17 | Wenqiang Fan, Tiandong Zhang | Xinxiang Central Hospital, The Fourth Clinical College of Xinxiang Medical University |
| 18 | Changsong Lin | The First Affiliated Hospital of Guangzhou University of Chinese Medicine |

Supplementary Methods

Inclusion/Exclusion Criteria

**Inclusion Criteria:**

**A subject will be eligible for inclusion in this study only if all of the following criteria apply:**

1. Males or females, aged ≥ 18 and ≤ 65 years old;
2. Diagnosed with systemic lupus erythematosus (SLE) according to the 2019 European League Against Rheumatism (EULAR) / American College of Rheumatology (ACR) classification criteria for SLE based on documented medical history and/or examination results.
3. SELENA-SLEDAI score > 0 and ≤ 12 during the screening period;
4. Positive serological findings for autoantibodies during the screening period, defined as: positive antinuclear antibody (ANA) and/or positive anti-ds-DNA antibody as determined based on the reference range of each study site's laboratory; borderline findings will not be accepted;
5. Maintain a stable standard of care (SOC) regimen for at least 30 days before the first dose of the investigational medicinal product (IMP). Stable standard of care regimen refers to the stable use of any of the following (alone or in combination): corticosteroids, antimalarials, nonsteroidal anti-inflammatory drugs (NSAIDs), immunosuppressants or immunomodulators, such as azathioprine, mycophenolate (including mycophenolate mofetil [MMF], mycophenolate sodium), methotrexate, leflunomide, tacrolimus, ciclosporin [Note: Routine dose reduction of corticosteroids ≤ 20 mg/d prednisone (or equivalent dose of other corticosteroids) will also be considered as stable therapy];
6. Subjects and their partners agree to use effective contraception during the following time periods (from screening to 4 months after the last dose of IMP for female subjects and their partners, from screening to 6 months after the last dose of IMP for male subjects and their partners);
7. Capable of understanding the nature of the study and voluntarily signing the informed consent form;
8. Able to communicate well with the investigator and complete all visits according to the protocol requirements.

**Exclusion Criteria:**

**A subject will not be eligible for inclusion in this study if any of the following criteria apply:**

1. Renal disorder: severe lupus nephritis (defined as urine protein > 6 g/24 hours or serum creatinine > 2.5 mg/dL or 221 μmol/L) within 8 weeks before the first dose of IMP, or active nephritis requiring treatment with drugs prohibited by the protocol, or conditions requiring hemodialysis, or treatment with prednisone ≥100 mg/d or equivalent glucocorticoids for ≥ 14 days;
2. Subjects with central nervous system diseases caused by SLE or non-SLE (including epilepsy, psychosis, organic encephalopathy syndrome, cerebrovascular accident, encephalitis, central nervous system vasculitis) within 8 weeks before the first dose of IMP;
3. The presence of the following laboratory test abnormalities, including but not limited to:
   1. Subjects with abnormal hepatic function, such as aspartate aminotransferase (AST) or alanine aminotransferase (ALT) > 2 times the upper limit of normal (ULN), total bilirubin > 1.5 times ULN, or
   2. Subjects with abnormal renal function: creatinine (Cr) or urea nitrogen (BUN) > 1.5 times ULN; screening glomerular filtration rate (eGFR) ≤ 60 mL/min/1.73 m^2^, or
   3. Subjects with abnormal hematology results: white blood cell count < 2.5×10^9^/L, hemoglobin < 85 g/L, platelet count < 50×10^9^/L;
4. Subjects with a history of clinically significant diseases that the investigator believes will pose a risk to the subject's safety, or that will affect the safety or efficacy analysis and evaluation if the disease/condition worsens during the study (such as abnormalities of the circulatory system and endocrine system, nervous system diseases, blood system diseases, immune system disorders, mental illness, and unstable metabolic abnormalities), for example: 1) cardiovascular disorder: history of acute myocardial infarction, or unstable angina pectoris, severe arrhythmia (multifocal premature ventricular contractions, ventricular tachycardia, ventricular fibrillation) within 6 months before screening; New York Heart Association (NYHA) Class III-IV; 2) subjects with known moderate or severe persistent asthma within 5 years before screening, or subjects with ongoing inadequately controlled asthma;
5. Subjects who may have active mycobacterial infection, defined as: chest X-ray (posterioranterior and lateral position) examination suggests active tuberculosis infection within 3 months prior to screening/during the screening period (if required by the ethics, tuberculosis testing will be performed according to site procedures);
6. Subjects with active hepatitis, positive hepatitis B surface antigen (HBsAg), positive hepatitis B core antibody (HBcAb) + positive hepatitis B virus (HBV) deoxyribonucleic acid (DNA), or positive hepatitis C virus (HCV) antibody at screening;
7. A history of human immunodeficiency virus (HIV) infection, or HIV antibody positive at screening;
8. Subjects with treponema pallidum antibody positive at screening;
9. Having chronic active infection or acute infection requiring systemic treatment with antibiotics, antivirals, antiparasites, antiprotozoals or antifungals within 4 weeks prior to screening, or having superficial skin infections requiring treatment within 1 week prior to screening. Note: After the infection is cured, the patient may be re-screened once;
10. Subjects with known or suspected history of immunosuppression, including history of invasive opportunistic infections (e.g., histoplasmosis, listeriosis, coccidioidomycosis, pneumocystosis, and aspergillosis), even if the infection has recovered; or have unusual frequent, recurrent, or prolonged infections (as judged by the investigator);
11. Major surgery (craniotomy, thoracotomy, or laparotomy) or unhealed wounds, ulcers, or fractures within 4 weeks prior to the first dose of IMP, or major surgery planned during the study;
12. Subjects with malignancy within 5 years prior to screening (except for completely cured cervical carcinoma in situ and non-metastatic squamous cell or basal cell carcinoma of the skin);
13. A history of major organ transplant (e.g., heart, lung, kidney, liver) or hematopoietic stem cell/or bone marrow transplant;
14. Subjects who received live or live attenuated vaccines within 4 weeks prior to the first dose of IMP or plan to receive the above during the study;
15. Subjects who have participated in any clinical trial within 4 weeks before the first dose of IMP or who are within 5 half-lives of the investigational product in the previous clinical trial (whichever is longer);
16. Targeted drug therapy: received rituximab within 180 days before the first dose of IMP; received any drug therapy targeting T or B lymphocytes, cytokines, or receptors (e.g., belimumab, telitacicept, abatacept, etc.) within 180 days before the first dose of IMP; received JAK inhibitor therapy within 30 days before the first dose of IMP; or within 5 half-lives after discontinuation of the above drugs (whichever is longer);
17. Received intravenous cyclophosphamide within 180 days before the first dose of IMP or oral cyclophosphamide within 30 days before the first dose of IMP;
18. Used intravenous immunoglobulins (IVIG), prednisone ≥ 100 mg/d or equivalent glucocorticoids, or undergone plasmapheresis within 28 days before the first dose of IMP;
19. Used IL-2, thalidomide, Tripterygium wilfordii Hook F, or drug products containing Tripterygium wilfordii Hook F within 28 days before the first dose of IMP;
20. Known allergy to monoclonal antibodies or to excipients of CM313;
21. Patients with depression or tendance for suicide;
22. A history of large alcohol consumption [i.e., more than 14 units of alcohol per week (1 unit = 360 mL of beer or 45 mL of liquor containing 40% alcohol or 150 mL of wine] or a history of drug abuse within 3 months prior to screening;
23. Female subjects who are pregnant or lactating, or planning to become pregnant or breastfeeding during the study; males whose partners plan to become pregnant during the study;
24. Any reason that, in the opinion of the investigator, contraindicates the subject's participation.

**The SOC regimen**

Subjects enrolled in this study needed to maintain a stable SOC for at least 30 days before the first dose of the study drug. The SOC regimen referred to the use of any of the following (alone or in combination): corticosteroids, antimalarials, NSAIDs, immunosuppressants or immunomodulators (including azathioprine, mycophenolate [including MMF and mycophenolic sodium], methotrexate, leflunomide, tacrolimus, ciclosporin).

Antimalarials in the SOC regimen: hydroxychloroquine (HCQ) maximum dose of 400 mg/day. Quinacrine (atabrine, mepacrine) maximum dose of 100 mg/day.

The maximum dose of corticosteroids (equivalent to prednisone dose) in the SOC regimen was 1 mg/kg/day, and maximal daily dose should be no more than 60 mg/day. For subjects with SLE treated with corticosteroids every other day, the average daily dose of corticosteroids was calculated as the average of the 2-day dose.

The maximum dose of azathioprine in the SOC regimen was 200 mg/day. The maximum dose of MMF was 2 g/day. The maximum dose of methotrexate was 25 mg/week. The maximum dose of ciclosporin was 4 mg/kg/day. The maximum dose of tacrolimus was 0.1 mg/kg/day. The maximum dose of leflunomide was 40 mg/day.

If the subject’s clinical condition changed, the SOC regimen was allowed to be adjusted as needed per the clinical condition, and the reason for adjusting the treatment regimen was recorded.

**Pre- and post-infusion medications**

**Pre-infusion medications (within 1-3 hours before infusion):**

- Corticosteroids (intermediate-acting or long-acting): It is recommended to administer 100 mg methylprednisolone (or equivalent drug) intravenously before the 1st and 2nd IMP administrations; starting from the 3rd IMP administration, the corticosteroid dose can be reduced to 60 mg methylprednisolone (or equivalent drug), orally or intravenously. If patients receive the aforementioned methylprednisolone (or equivalent drug) as pre-infusion medication on the IMP administration day, they should not receive additional corticosteroids as part of their standard SLE treatment.
- Paracetamol, 650 mg to 1000 mg, oral.
- Antihistamines: Diphenhydramine, 25 mg to 50 mg (or equivalent), oral or intramuscular.

**Post-infusion medications:**

- Starting from the day after the completion of the infusion, subjects should receive corticosteroids for 2 consecutive days (20 mg methylprednisolone or equivalent drug daily). If the oral corticosteroid dose in the subject's standard SLE treatment is ≥ the above dose, no additional corticosteroids will be given; if the oral corticosteroid dose in the subject's standard SLE treatment is < the above dose, corticosteroids will be supplemented to the above dose).
- In addition, for subjects with a history of chronic obstructive pulmonary disease (COPD), post-infusion medications should be considered, including short-acting and long-acting bronchodilators and inhaled corticosteroids.

**Glucocorticoids dosing tapering**

Glucocorticoids dosing tapering was at the investigator’s discretion according to Expert Consensus on the Rational Use of Glucocorticoids in Patients with Systemic lupus Erythematosus (China, 2014). During the maintenance treatment period, the dose of glucocorticoids was slowly reduced at a rate of 10% of the original dose every 1-2 weeks. After the dose was reduced to 0.5 mg/kg/d of prednisone (equivalent to 0.4 mg/kg/d of methylprednisolone), the rate of dose reduction was appropriately slowed down according to the disease condition.

**Flow cytometry**

Flow cytometry, performed with the Beckman CytoFLEX S, was used to evaluate changes in different immune cells in whole blood. A mixture of fluorescent antibodies was added to BD Trucount tubes containing a known number of fluorescent counting beads, followed by the addition of peripheral blood samples. After incubation and red blood cell lysis, the samples were analyzed using a flow cytometer. A scatter plot was used to gate the target cell population. Gating strategies were as follows:

Panel A:

- Lymphocytes were gated with FSC-A-SSC-A.
- Then single cells were gated with FSC-A-FSC-H to exclude cell doublets.
- CD45 positive cells were then gated with CD45+.
- CD38+ T cells were gated with CD3+CD38+ upon CD45+ cells.
- CD38+ B cells were gated with CD3-CD19+CD38+ upon CD45+ cells.
- CD38+ NK cells were gated with CD3-CD16+CD56+CD38+ upon CD45+ cells.

Panel B:

- Peripheral blood mononuclear cells (PBMCs) were gated with FSC-A-SSC-A.
- Single cells were gated with FSC-A-FSC-H to exclude cell doublets.
- CD38+ pDC cells were gated with CD123+BDCA2+CD38+.

The absolute number of target cells was calculated based on the ratio of target cells to fluorescent counting beads. The absolute number of target cells (cells/μL) = (events of target cells / events of fluorescent beads) × (number of fluorescent beads per tube / volume of peripheral blood in μL). The proportion of target cells is the percentage of target cells within the lymphocyte population. After gating the lymphocytes, the flow cytometry software CytoExpert automatically calculated the cell proportion.

The work was performed at Triaplex Biotechnology.

**Assessments of the disease scores**

The Lupus Erythematosus National Assessment-Systemic Lupus Erythematosus Disease Activity Index (SELENA-SLEDAI) measures disease activity in SLE patients over the past 10 days and primarily evaluates the presence of 24 clinical characteristics across 9 organ systems. The total SELENA-SLEDAI score is the sum of the weighted scores, with a total score range of 0 to 105 and higher total scores indicating more severe disease. Evaluable patients for a ≥4-point reduction in SELENA-SLEDAI score from baseline were defined as those with a SELENA-SLEDAI score ≥4 at baseline.

The Physician’s Global Assessment (PGA) score is a physician's assessment of the overall disease activity in SLE patients over the past 2 weeks using a visual analogue scale, with 0 points representing no activity, 1 point representing mild activity, 2.0 to 2.5 points representing moderate activity, and 3 points representing severe activity. Improved PGA score was defined as ≥0.3-point reduction from baseline, and evaluable patients were defined as those with a PGA score ≥0.3 at baseline.

The British Isles Lupus Assessment Group 2004 index (BILAG-2004) assesses changes in the patient's clinical manifestations over the past 4 weeks compared to the previous 4 weeks (not present, improving, same, worse, or new), based on the physician's treatment intent, including 97 clinical signs, symptoms, and laboratory parameters across 9 organ systems. A five-level system is used to assess the degree of disease change in all 9 organ systems: A (severe), B (moderate), C (mild), D (inactive), E (inactive with no previous involvement). Improved BILAG-2004 score was defined as no new disease activity as measured by ≥1 A score or ≥2 B scores (a baseline A score reducing to B was not a new B score), and improvement in at least one organ domain (defined as a baseline A score reducing to B, C, or D, or a baseline B score reducing to C or D). Evaluable patients for an improved BILAG-2004 score were defined as those with at least one A or B score on BILAG-2004 at baseline.

Evaluable patients for a Systemic Lupus Erythematosus Responder Index (SRI)-4 response were defined as those with a SELENA-SLEDAI score ≥4, not all A scores in all organ domains on BILAG-2004, and a PGA score ≤2.7 at baseline.

The assessments of SELENA-SLEDAI and PGA scores were performed at V1 (screening visit), V2 (baseline visit), V4, V6, V8, V10 (EOT visit), V11, V12, V13, and V14 (EOS visit). The BILAG-2004 score was assessed at V2, V6, V10, V12, and V14. The number of SLE flares and the severity of each flare, as well as the date of each flare, were recorded on V2, V4, V6, V8, V10, V11, V12, V13, and V14.

**Assessment of serologic markers**

Serum concentrations of IgG, IgA, IgM, and complement C3 and C4 were determined using immunoturbidimetry. The analytes in serum were bound to IgG, IgA, IgM, C3, and C4 specific antibodies, and insoluble immune complex particles were shown by measuring the specific turbidity at 340 nm for IgM and C4, 600 nm for C3, and 700 nm for IgAand IgG absorbance.

Serum concentration of IgE was determined using nephelometry, which was designed to measure the scattering coefficient. The polystyrene particles coated with specific IgE antibodies in the dispersion solution interact with serum IgE and caused the immuno-aggregation of polystyrene particles.

Serum concentrations of anti-dsDNA antibodies in human serum were determined using a validated Enzyme linked immunosorbent assay (ELISA). Microplates were coated with ds-DNA antibodies, samples were added, and the bounded analyte detected by enzyme-labeled antibody.

Figure. S1.

**Serum CM313 concentration-time curve.**

**a, b** Serum CM313 concentration after the first infusion. **c, d** Serum CM313 concentration after the last infusion. Error bars denote standard errors.

Figure. S2.

**Median percentage change from baseline in other pharmacodynamic markers.**

**a** CD38+ B cell count. **b** CD38+ T cell count. **c** CD38+ plasmacytoid dendritic cell (pDC) count. Red triangle marks indicate the time points of CM313 or placebo administrations.

Figure. S3.

**Improvement in PGA score.**

**a** Mean change from baseline (BL) in Physician Global Assessment (PGA) score. Error bars denote standard errors. All enrolled patients were evaluable. **b** The proportion of patients achieving improved PGA score, defined as ≥0.3-point reduction from baseline. N indicates the number of evaluable patients, defined as those with a PGA score ≥0.3 at baseline.

Figure. S4.

**The proportion of patients achieving improved BILAG-2004 score from baseline.**

Improved British Isles Lupus Assessment Group-2004 (BILAG-2004) score was defined as no new disease activity as measured by ≥1 A score or ≥2 B scores (a baseline [BL] A score reducing to B was not a new B score), and improvement in at least one organ domain (defined as a baseline A score reducing to B, C, or D, or a baseline B score reducing to C or D). N indicates the number of evaluable patients, defined as those with at least one A or B score on BILAG-2004 at baseline.

Figure. S5.

**Study design.**

The trial comprised a screening period of up to 4 weeks, an 8-week treatment period, and an 8-week follow-up period. The next dose level was initiated after all subjects in the previous dose level had completed safety observation (D1 to D28) without any adverse events meeting the interruption and stopping criteria for dose escalation as judged by the investigator and the sponsor. Black arrows show the time points of administration of the study drug. D=day.

Table S1. Dose proportional relationship of major pharmacokinetic parameters following the first and last dose

|  | Dose groups | Slope | 90% confidence interval |
| --- | --- | --- | --- |
| **Following the first dose** | | | |
| Ln (C_max_) | 2 mg/kg (n=8), 4 mg/kg (n=8), 8 mg/kg (n=8), 16 mg/kg (n=8) | 1.16 | 1.08-1.24 |
| Ln (AUC_0-t_) | 2 mg/kg (n=8), 4 mg/kg (n=8), 8 mg/kg (n=8), 16 mg/kg (n=8) | 1.65 | 1.51-1.80 |
| **Following the last dose** | | | |
| Ln (C_max_) | 2 mg/kg (n=8), 4 mg/kg (n=7), 8 mg/kg (n=8), 16 mg/kg (n=8) | 1.19 | 1.10,1.28 |
| Ln (AUC_0-t_) | 2 mg/kg (n=8), 4 mg/kg (n=7), 8 mg/kg (n=8), 16 mg/kg (n=8) | 1.63 | 1.41-1.84 |
| Ln (AUC_0-∞_) | 2 mg/kg (n=5), 4 mg/kg (n=7), 8 mg/kg (n=8), 16 mg/kg (n=8) | 1.43 | 1.23-1.63 |

*C_max_* maximum concentration, *AUC* area under the curve

Table S2. Pharmacokinetic endpoints

| **Parameters** | **CM313,**  **2 mg/kg** | **CM313,**  **4 mg/kg** | **CM313,**  **8 mg/kg** | **CM313,**  **16 mg/kg** |
| --- | --- | --- | --- | --- |
| **Following the first dose** | | | | |
| n | 8 | 8 | 8 | 8 |
| T_max_, h | 6.60 (3.95–10.47) | 7.07 (6.02–11.42) | 11.01 (6.88–17.07) | 8.83 (6.80–14.17) |
| C_max_, ug/mL | 45.8 (9.91) | 84.8 (13.9) | 209 (35.3) | 494 (106) |
| AUC_0-168h_, h×ug/mL | 2880 (1170) | 7080 (1870) | 18600 (2560) | 45900 (12700) |
| AUC_0-t_, h×ug/mL | 3190 (1630) | 9540 (3340) | 33300 (6730) | 88300 (25800) |
| AUC_0-∞_, h×ug/mL | 3240 (1610) | 9640 (3380) | 34900 (8190) | 99100 (35100) |
| t_1/2z_, h | 39.7 (10.8) | 67.0 (23.5) | 130 (58.0) | 173 (72.7) |
| CL, mL/h | 41.3 (16.1) | 23.5 (7.89) | 13.1 (2.83) | 10.6 (4.44) |
| V_z_, mL | 2180 (320) | 2140 (537) | 2280 (774) | 2400 (913) |
| **Following the last dose** | | | | |
| n | 8 | 7 | 8 | 8 |
| T_max_, h | 4.96 (3.78–8.68) | 9.10 (6.55–29.83) | 10.00 (6.45–14.05) | 8.17 (6.30–11.30) |
| C_max_, ug/mL | 85.2 (23.7) | 178 (24.7) | 414 (59.5) | 1030 (348) |
| AUC_tau_^*^, h×ug/mL | 8990 (2700) | 19800 (2830) | 47900 (10800) | 117000 (37200) |
| AUC_0-t_, h×ug/mL | 15300 (12600) | 50000 (11500) | 153000 (63600) | 378000 (157000) |
| AUC_0-∞_, h×ug/mL | 22000 (12700) | 50100 (11400) | 160000 (71100) | 405000 (179000) |
| t_1/2z_, h | 128 (49.6) | 110 (42.9) | 241 (145) | 316 (138) |
| CL, mL/h | 13.5 (3.13) | 10.5 (2.24) | 9.53 (2.08) | 8.50 (2.78) |
| V_z_, mL | 3300 (1200) | 3380 (559) | 4010 (639) | 3760 (1270) |
| R_ac,Cmax_ | 1.85 (0.226) | 2.18 (0.375) | 2.00 (0.230) | 2.10 (0.543) |
| R_ac,AUC_^†^ | 3.43 (1.37) | 3.05 (0.570) | 2.59 (0.552) | 2.70 (0.864) |

Data are mean (SD) or median (range).

^*^AUC_tau_: Area under the serum concentration-time curve from dosing time to dosing time plus 168 h.

^†^R_ac_AUC_=AUC_tau_/AUC_0-168 h._

*n* number, *T_max_* time to maximum concentration, *SD* standard deviation, *C_max_* maximum concentration, *AUC* area under the curve, *t_1/2z_* elimination half-life, *CL* apparent clearance, *V_z_* apparent volume of distribution, *R_ac_* accumulation index

Table S3. Changes in average daily prednisone (or equivalent) dose during the trial

| **Patient ID** | **Dose group** | **Average daily dose at baseline** | **Average daily dose during the trial** |
| --- | --- | --- | --- |
| 05001 | CM313 2 mg/kg | 15 mg | The average daily prednisone (or equivalent) dose was reduced to 6.429 mg at day 29, 5 mg at day 36 and maintained until day 113. |
| 06001 | CM313 2 mg/kg | 17.5 mg | The average daily prednisone (or equivalent) dose was reduced to 15 mg at day 8, 12.5 mg at day 29, 10.625 mg at day 36, 10 mg at day 43, 7.5 mg at day 85 and maintained until day 113. |
| 13001 | CM313 2 mg/kg | 15 mg | The average daily prednisone (or equivalent) dose was reduced to 12.5 mg at day 15, 10 mg at day 71, 7.5 mg at day 99, and 5.357 mg at day 113. |
| 18004 | CM313 4 mg/kg | 1.071 mg | The average daily prednisone (or equivalent) dose was changed to 1.250 mg at day 8, 1.429 mg at day 15, 1.071 mg at day 22, 1.5 mg at day 29, 1.250 mg at day 36, 1.5 mg at day 43, 1.25 mg at day 50, 1.429 mg at day 71, and 1.071 mg at day 113. |
| 08001 | CM313 8 mg/kg | 10 mg | The average daily prednisone (or equivalent) dose was reduced to 7.5 mg at day 43 and maintained until day 113. |
| 09006 | CM313 8 mg/kg | 20 mg | The average daily prednisone (or equivalent) dose was reduced to 15 mg at day 99 and maintained until day 113. |
| 17005 | CM313 8 mg/kg | 7.857 mg | The average daily prednisone (or equivalent) dose was changed to 8.0 mg at day 8, 7.5 mg at day 15, 7.143 mg at day 22, 7.5 mg at day 36, 8.0 mg at day 57, 7.143 mg at day 71, 7.857 mg at day 85 and maintained until day 113. |
| 13006 | CM313 16 mg/kg | 2.857 mg | The average daily prednisone (or equivalent) dose was changed to 2.5 mg at day 8, 2.857 mg at day 15, 2.143 mg at day 22, 2.727 mg at day 36, 2.5 mg at day 43, 3.0 mg at day 57, 2.143 mg at day 71, and 2.857 mg at day 113. |
| 13007 | CM313 16 mg/kg | 15 mg | The average daily prednisone (or equivalent) dose was changed to 13.448 mg at day 8, 15.0 mg at day 15, 12.5 mg at day 29, 10.625 mg at day 57, 10 mg at day 71 and maintained until day 113. |
| 09005 | Placebo | 30 mg | The average daily prednisone (or equivalent) dose was reduced to 25 mg at day 113. |
| 14002 | Placebo | 6.25 mg | Prednisone was discontinued from day 99 until day 113. |
