# Supplementary material for: Anti-CD38 monoclonal antibody CM313 for systemic lupus erythematosus: a randomized, double-blind, placebo-controlled phase Ib/IIa trial
Source: Signal Transduct Target Ther. 2025 Nov 26;10:383. doi: 10.1038/s41392-025-02487-2 (PMC12647876; doi:10.1038/s41392-025-02487-2)
Supplement: Supplementary file 2 — Clinical Study Protocol [file 41392_2025_2487_MOESM2_ESM.pdf]

Registration Category: Category 1 of Therapeutic Biological Products

# Clinical Study Protocol

# A Randomized, Double-blind, Placebo-controlled, Dose-escalating, Multiple-dose Phase 1b/2a Clinical Study to Evaluate the Safety, Tolerability, Pharmacokinetics, Pharmacodynamics, Immunogenicity, and Preliminary Efficacy of CM313 Injection in Subjects with Systemic Lupus Erythematosus

Protocol No.: CM313-106001

Leading Clinical Study Site: Peking Union Medical College Hospital

Principal Investigator: Xiaofeng Zeng

Clinical Study Sponsor: Keymed Biosciences (Chengdu) Co., Ltd.

Sponsor's Principal Representative: Bo Chen

Version No.: 2.0

Version Date: 18 Jul 2022

**Confidentiality Statement**

The information in this document contains trade secrets or commercial information of the drug registration applicant, which are privileged or confidential, and may not be disclosed unless such disclosure is required by applicable laws or regulations. Any individual receiving the document should be aware of its proprietary and confidential nature and shall not disclose it to others. Restrictive clause in the confidentiality of the above document shall apply to any future proprietary or confidential documents and materials provided to you.

## Summary of Changes in the Protocol

After the initial submission of this study protocol to the ethics committee, the description of relevant scales in the appendices of version 1.0 was updated, so the protocol was upgraded to version 2.0 (version date: 18 Jul 2022) with the following amendments:

| Revised Section/Content | Content before Revision | Content after Revision | Reason for Revision |
|---|---|---|---|
| Version and Date | Version: 1.0<br>Version Date: 02 Apr 2022 | Version: **2.0**<br>Version Date: **18 Jul 2022** | The protocol was updated, and version number and date were updated accordingly |
| Appendix 2 | a. Clinical manifestations of seizure: Recent onset (last 10 days). Exclude metabolic, infectious or drug cause, or seizure due to past related CNS damage;<br>b. Clinical manifestations of alopecia: Abnormal patchy or diffuse loss of hair;<br>c. Clinical manifestations of rash: Inflammatory lupus rash.<br>d. Clinical manifestation of mucosal ulcers: Oral or nasal mucosal ulcerations.<br>e. Clinical manifestations of pleurisy: Classic or severe pleuritic chest pain, pleural rub, effusion or new pleural thickening;<br>f. Clinical manifestations of pericarditis: Pericardial pain accompanied by at least one of the following: pericardial rub, pericardial effusion, or electrocardiogram confirmation.<br>g. Clinical manifestations of thrombocytopenia: < 100 × $10^9$/L. Exclude drug causes; | a. Clinical manifestations of seizure: Recent onset (last 10 days). Exclude metabolic, infectious or drug cause, or seizure due to past **irreversible** CNS damage;<br>b. Clinical manifestations of alopecia: Ongoing abnormal, patchy or diffuse loss of hair **due to active lupus**;<br>c. Clinical manifestations of rash: **Ongoing** inflammatory lupus rash;<br>d. Clinical manifestation of mucosal ulcers: **Ongoing** oral or nasal ulcerations **due to active lupus**;<br>e. Clinical manifestations of pleurisy: Classic **and** severe pleuritic chest pain **or** pleural rub **or** effusion or new pleural thickening **due to lupus**;<br>f. Clinical manifestations of pericarditis: **Classic and severe** pencardial pain **or** rub **or** effusion, **or** electrocardiogram confirmation;<br>g. Clinical manifestations of thrombocytopenia: < 100 × $10^9$/L; | Consistent with the content of the original English scale |
| Appendix 4 | Scoring Criteria<br>or circle Yes/No | Scoring Criteria<br>or Yes/No<br>*Yes/No Circle “Yes” or “No” to indicate whether | Consistent with the content of the original English scale |

| | | | |
|---|---|---|---|
| | *Yes/No Circle "Yes" or "No" to indicate whether the abnormal value (according to BIALG) is caused by SLE.<br><br>80. Accelerated hypertension Yes/No<br>81. Urine dipstick protein (abnormal value ≥ + 1) value ( ) Yes/No*<br>85. Nephrotic syndrome value ( ) Yes/No*<br>88. Active urinary sediment Yes/No<br>89. Active nephritis Yes/No<br>91. Total white cell count (abnormal value ≤ $3.9 \times 10^9$ g/dL) value ( ) Yes/No*<br>92. Neutrophils (abnormal value ≤ $1.9 \times 10^9$ g/dL) value ( ) Yes/No*<br>93. Lymphocytes (abnormal value ≤ $1.0 \times 10^9$ g/dL) value ( ) Yes/No*<br>94. Platelets (abnormal value ≤ $149 \times 10^9$ g/dl) value ( ) Yes/No*<br>96. Evidence of active hemolysis Yes/No<br>97. Coomb's test positive (isolated) Yes/No | the abnormal value is caused by SLE.<br><br>80. Accelerated hypertension Yes/No **( )**<br>81. Urine dipstick protein (+=**1**, ++=**2**, +++=**3**) value ( ) Yes/No*<br>85. Nephrotic syndrome Yes/No **( )**<br>88. Active urinary sediment Yes/No **( )**<br>89. Active nephritis Yes/No **( )**<br>91. Total white cell count (abnormal value ≤ $3.9 \times 10^9$/**L**) value ( ) Yes/No*<br>92. Neutrophils (abnormal value ≤ $1.9 \times 10^9$/**L**) value ( ) Yes/No*<br>93. Lymphocytes (abnormal value ≤ $1.0 \times 10^9$/**L**) value ( ) Yes/No*<br>94. Platelets (abnormal value ≤ $149 \times 10^9$/**L**) value ( ) Yes/No*<br>96. Evidence of active hemolysis Yes/No **( )**<br>97. Coomb's test positive (isolated) Yes/No **( )** | |

## Protocol Synopsis

<table>
<tr><td colspan="2">Sponsor: Keymed Biosciences (Chengdu) Co., Ltd.</td></tr>
<tr><td colspan="2">Title: A Randomized, Double-blind, Placebo-controlled, Dose-escalating, Multiple-dose Phase 1b/2a Clinical Study to Evaluate the Safety, Tolerability, Pharmacokinetics, Pharmacodynamics, Immunogenicity, and Preliminary Efficacy of CM313 Injection in Subjects with Systemic Lupus Erythematosus</td></tr>
<tr><td colspan="2">Test Drug: CM313 Injection (hereinafter referred to as "CM313")</td></tr>
<tr><td colspan="2">Number of Study Sites: Multicenter</td></tr>
<tr><td colspan="2">Study Phase: 1b/2a</td></tr>
<tr><td colspan="2">Study Period: The maximum duration of study participation for each subject is expected to be approximately 20 weeks, including a screening period of up to 4 weeks, a treatment period of 8 weeks, and a follow-up period of 8 weeks.</td></tr>
<tr><td colspan="2">Planned Study Duration: Approximately 1 year.</td></tr>
<tr><td colspan="2">Study Objectives and Study Endpoints:</td></tr>
<tr><td>Primary Objective</td><td>Primary Endpoint</td></tr>
<tr><td>To evaluate the safety and tolerability of multiple doses of CM313 in subjects with systemic lupus erythematosus (SLE).</td><td>Safety endpoints: adverse events (AEs), laboratory tests, physical examinations, vital signs, 12-lead electrocardiogram (ECG) abnormalities, etc.</td></tr>
<tr><td>Secondary Objectives</td><td>Secondary Endpoints</td></tr>
<tr><td>➢ To evaluate the pharmacokinetic (PK) characteristics of multiple doses of CM313;<br>➢ To evaluate the pharmacodynamic (PD) characteristics of multiple doses of CM313;<br>To evaluate the immunogenicity of multiple doses of CM313.</td><td>➢ PK endpoints:<br>• PK parameters after the first dose of CM313, including time to maximum concentration ($T_{max}$), maximum concentration ($C_{max}$), area under the serum concentration-time curve over a dosing interval ($AUC_{tau}$), area under the serum concentration-time curve from time 0 to time t ($AUC_{0-t}$), etc.;<br>• PK parameters after the last dose of CM313, including $T_{max}$, $C_{max}$, $AUC_{tau}$, $AUC_{0-t}$, area under the concentration-time curve from 0 to ∞ ($AUC_{0-\infty}$), elimination half-life ($T_{1/2z}$), clearance (CL), volume of distribution ($V_z$), mean residence time ($MRT_{0-t}$), and accumulation index ($R_{ac}$) , etc.<br>➢ PD endpoints:<br>• Change and percentage change from baseline in immune cells (including T lymphocytes, B lymphocytes, pDC cells, and NK cells).<br>Immunogenicity endpoint: anti-drug antibodies (ADAs).</td></tr>
<tr><td>Exploratory Objectives</td><td>Exploratory Endpoints</td></tr>
<tr><td>To explore the preliminary efficacy and some PD characteristics of CM313 after multiple doses.</td><td>➢ Preliminary efficacy endpoints:<br>• Change from baseline in the SELENA-SLEDAI score at each evaluation visit;<br>• Change from baseline in Physician Global Assessment (PGA) score at each evaluation visit;<br>• Time to first SLE flare after randomization;<br>• Change from baseline in prednisone dosage at each evaluation visit;</td></tr>
</table>

| | |
|---|---|
| | • Change from baseline in the BILAG-2004 score at each evaluation visit;<br>• Changes from baseline in 24-hour protein urine quantification and protein urine/creatinine ratio (only for subjects with protein urine/creatinine ratio exceeding the upper limit of normal during the screening period) at each evaluation visit;<br>• Percentage change from baseline in immunology-related endpoints [immunoglobulin G (IgG), immunoglobulin A (IgA), immunoglobulin M (IgM), immunoglobulin E (IgE), anti-double-stranded deoxyribonucleic acid (ds-DNA) antibodies, complement (C3, C4)], C-reactive protein (CRP), and erythrocyte sedimentation rate (ESR) at each evaluation visit.<br>The expression levels of type I interferon (IFN)-related genes and their changes and rates of change from baseline at each assessment visit. |

**Study Design**

This is a multicenter, randomized, double-blind, multiple-dose, dose-escalation, placebo-controlled Phase 1b/2a trial designed to evaluate the safety, tolerability, PK, PD, immunogenicity, and preliminary efficacy of CM313 in subjects with SLE.

The study plans to enroll 40 SLE subjects, with a total of 4 dose levels: 2 mg/kg, 4 mg/kg, 8 mg/kg, and 16 mg/kg; and the dose escalation starts from the lowest dose. Ten subjects are to be enrolled at each dose level and randomized in a 4: 1 ratio to receive the respective dose of CM313 (8 subjects) or placebo (2 subjects). Both CM313 and placebo will be administered intravenously. Subjects will be observed for 28 days after the first dose (single-dose phase), followed by once-weekly (QW) administration for a total of 4 doses (multiple-dose phase). The 16 mg/kg doselevel is optional. After all subjects at the 8 mg/kg dose level have completed the single-dose safety observation period, the sponsor and the investigator will decide whether to proceed with the 16 mg/kg dose level based on the safety data.

The multiple dose escalation scheme for this study is as follows:

| **Dose Level** | **2 mg/kg** | **4 mg/kg** | **8 mg/kg** | **16 mg/kg** |
|---|---|---|---|---|
| Total Subjects<br>(CM313: placebo) | 10<br>(4:1) | 10<br>(4:1) | 10<br>(4:1) | 10<br>(4:1) |
| Escalation Magnitude | Starting dose | 100% | 100% | 100% |

The study design schema is as follows (black arrows in the figure indicate drug administration):

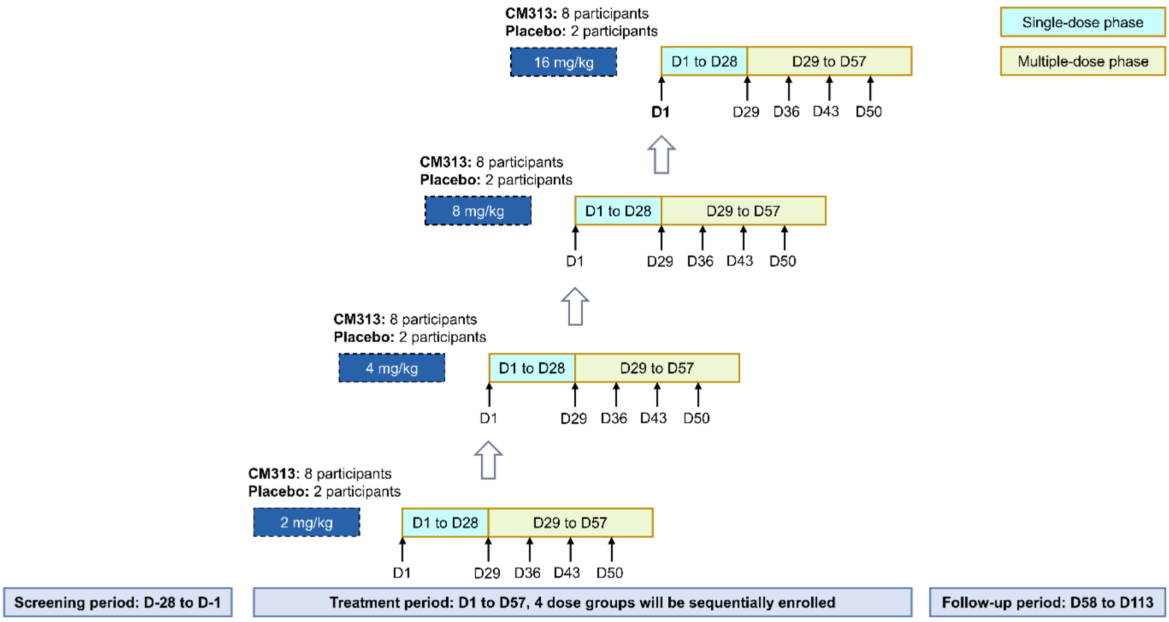


This study will consist of three periods: a screening period of up to 4 weeks, an 8-week treatment period, and an 8-week follow-up period.

**Screening Period (4 weeks)**

All participants who have signed the informed consent form (ICF) will be screened for eligibility within 28 days prior to the first dose.

If a subject is screened as ineligible due to an incidental, temporary condition (e.g., insufficient washout from prior therapy, etc.), re-screening may be performed once, but the ICF needs to be re-signed and a new screening number should be assigned.

**Treatment Period (8 weeks)**

The treatment period includes a single-dose phase (Day 1 to Day 28) and a multiple-dose phase (Day 29 to Day 57). Successfully enrolled participants will be randomized prior to the first dose administration and will receive the corresponding dose of CM313 or placebo via intravenous infusion according to their assigned dose level. Subjects will be observed for 28 days (single-dose phase) after the first dose (D1). After the safety and tolerability are assessed as acceptable by the investigator, subjects will enter the multiple-dose phase and receive four consecutive doses (QW) of the investigational medicinal product (IMP) (multiple-dose phase). The administration for the next dose level can be initiated after completion of the single-dose phase in the previous dose level, and that there are no AEs meeting the dose escalation interruption and stopping criteria as evaluated by the investigator and the sponsor. The details are as follows:

- **Dose Levels:** Four dose levels are planned: 2 mg/kg, 4 mg/kg, 8 mg/kg, and 16 mg/kg.
- **Number of Subjects:** Each dose level will include 10 subjects (8 subjects will receive CM313, and 2 subjects will receive placebo).
- **Route and Frequency of Administration:**

  Both CM313 and placebo will be administered intravenously.
  - **Single-dose phase (D1 to D28):** On D1, subjects will receive the corresponding dose of CM313 or placebo according to their assigned dose level via intravenous infusion. Following administration, they will enter a 28-day safety observation period.
  - **Multiple-dose phase (D29-D57):** On D29, subjects will enter the multiple-dose phase and receive the corresponding dose of CM313 or placebo via intravenous infusion, once weekly (QW), for a total of 4 doses. After completing all doses, subjects will have an end of treatment (EOT) visit 1 week after the last dose.
- **Dose escalation:** The next dose level can be initiated after all subjects in the previous dose level have completed D1-D28 safety observation without any AEs meeting the interruption and

stopping criteria for dose escalation as judged by the investigator and the sponsor.

**Follow-up Period (8 weeks)**

After completing the EOT visit, subjects will enter an 8-week follow-up period until the end of study (EOS) visit is completed.

**Dose Escalation**

The investigators and the sponsor will determine whether to proceed to the next dose level based on the safety findings of the single-dose phase for the previous dose level, following the principle of dose escalation.

Upon completion of the pre-defined multiple-dose administration and based on the obtained CM313 PK parameters, PD, safety, and efficacy data, the investigator and sponsor will jointly discuss whether to add new dose levels (including adjustments to dosing frequency and dose levels) and adjust sampling time pointsa.

**Standard of Care**

All subjects will maintain a stable standard of care regimen for at least 30 days before the first dose of IMP and will continue the standard of care regimen during the trial. The standard of care regimen refers to the use of any of the following (alone or in combination): corticosteroids, antimalarials, nonsteroidal anti-inflammatory drugs (NSAIDs), immunosuppressants or immunomodulators, such as azathioprine, mycophenolate (including mycophenolate mofetil, mycophenolic sodium), methotrexate, leflunomide, tacrolimus, ciclosporin. If the subject's clinical condition changes, the standard of care regimen is allowed to be adjusted as needed per the clinical condition, and the reason for adjusting the treatment regimen needs to be recorded.

**Dose Modifications**

Dose modifications of IMP received by individual subjects are not allowed during the treatment period. However, if dosing is interrupted due to an AE or other reasons, the investigator will consider resuming dosing, discontinuing treatment, or withdrawing the subject from the study depending on the specific circumstances.

**Premature Discontinuation of Treatment**

If a subject discontinues treatment prematurely but does not withdraw from the study, they should complete the EOT visit and subsequent follow-up visits until the EOS visit, as far as possible.

**Early Withdrawal from the Study**

If a subject withdraws early from the study, the "early withdrawal" visit should be completed and blood samples for PK, PD, and immunogenicity should be collected. The procedures, examinations and assessments required for early withdrawal visit are the same as those for the EOS visit, and the sample collection time or safety examination time should be as close as possible to the time of withdrawal from the study.

**Unscheduled Visits**

Throughout the study, the investigator may arrange unscheduled visits based on the actual situation of each subject. Clinically significant results from procedures, examinations and assessments at unscheduled visits should be recorded in the clinical medical records and case report forms (CRFs). If clinically significant abnormalities are identified, the investigator will determine whether the subject needs to undergo reexamination, treatment, or hospitalization observation and the subject will be followed up until recovery or stabilized outcome.

**Dose Escalation Interruption and Stopping Criteria:**

After completion of the safety observation period following the first dose of IMP for subjects at each dose level, safety will be assessed, and the relationship between each AE and the IMP will be evaluated. AEs that are "definitely related", "probably related", and "possibly related" will be considered related to the IMP. The investigator and the sponsor will decide whether to continue dose escalation of the IMP based on the safety and tolerability data obtained from all subjects who have completed the previous dose level.

The interruption of dose escalation may be considered if moderate or higher IMP-related AEs with the same system organ class (SOC) are observed in 50% or more of the subjects at that dose level, or if unacceptable pharmacological effects reasonably attributable to the IMP occur, or if more than 1 subject experiences a serious adverse event (SAE) in the same SOC.

**If dose escalation is interrputed, the investigator will decide on one of the following solutions after discussion with the sponsor:**

- Continue to escalate to the next dose level as specified in the protocol; or
- Add an intermediate dose or adjust dosing frequency.

**If the same reason for interruption of dose escalation occurs for a second time during the study, dose escalation should be stopped.**

Note: AEs definitely due to other reasons will not be considered AEs that meet the interruption and stopping criteria for dose escalation. If any IMP-related SAE occurs during the study, the study needs to be suspended, and the investigator will discuss and analyze the reasons with the sponsor and determine the impact on the subsequent study.

**Inclusion/Exclusion Criteria**

**Inclusion Criteria:**

**A subject will be eligible for inclusion in this study only if all of the following criteria apply:**

1. Males or females, aged ≥ 18 and ≤ 65 years old;
2. Diagnosed with SLE according to the 2019 European League Against Rheumatism (EULAR)/American College of Rheumatology (ACR) classification criteria for SLE (see Appendix 1) based on documented medical history and/or examination results.
3. SELENA-SLEDAI score > 0 and ≤ 12 during the screening period;
4. Positive serological findings for autoantibodies during the screening period, defined as: positive antinuclear antibody (ANA) and/or positive anti-ds-DNA antibody as determined based on the reference range of each study site's laboratory; borderline findings will not be accepted;
5. Maintain a stable standard of care regimen for at least 30 days before the first dose of IMP. Stable standard of care regimen refers to the stable use of any of the following (alone or in combination): corticosteroids, antimalarials, NSAIDs, immunosuppressants or immunomodulators, such as azathioprine, mycophenolate (including mycophenolate mofetil, mycophenolate sodium), methotrexate, leflunomide, tacrolimus, ciclosporin [Note: Routine dose reduction of corticosteroids ≤ 20 mg/d prednisone (or equivalent dose of other corticosteroids) will also be considered as stable therapy];
6. Subjects and their partners agree to use effective contraception during the following time periods (from screening to 4 months after the last dose of IMP for female subjects and their partners, from screening to 6 months after the last dose of IMP for male subjects and their partners) (see Appendix 6);
7. Capable of understanding the nature of the study and voluntarily signing the ICF;
8. Able to communicate well with the investigator and complete all visits according to the protocol requirements.

**Exclusion Criteria:**

**A subject will not be eligible for inclusion in this study if any of the following criteria apply:**

1. Renal disorder: Severe lupus nephritis (defined as urine protein > 6 g/24 hours or serum creatinine > 2.5 mg/dL or 221 μmol/L) within 8 weeks before the first dose of IMP, or active nephritis requiring treatment with drugs prohibited by the protocol, or conditions requiring hemodialysis, or treatment with prednisone ≥100 mg/d or equivalent glucocorticoids for ≥ 14 days;
2. Subjects with central nervous system diseases caused by SLE or non-SLE (including epilepsy, psychosis, organic encephalopathy syndrome, cerebrovascular accident, encephalitis, central nervous system vasculitis) within 8 weeks before the first dose of IMP;
3. The presence of the following laboratory test abnormalities, including but not limited to:
   a) Subjects with abnormal hepatic function, such as aspartate aminotransferase (AST) or alanine aminotransferase (ALT) > 2 times the upper limit of normal (ULN), total bilirubin > 1.5 times ULN, or
   b) Subjects with abnormal renal function: creatinine (Cr) or urea nitrogen (BUN) > 1.5 times ULN; screening glomerular filtration rate (eGFR) ≤ 60 mL/min/1.73 $m^2$, or
   c) Subjects with abnormal hematology results: white blood cell count < $2.5\times10^9$/L, hemoglobin < 85 g/L, platelet count < $50x10^9$/L;

4. Subjects with a history of clinically significant diseases that the investigator believes will pose a risk to the subject's safety, or that will affect the safety or efficacy analysis and evaluation if the disease/condition worsens during the study (such as abnormalities of the circulatory system and endocrine system, nervous system diseases, blood system diseases, immune system disorders, mental illness, and unstable metabolic abnormalities), for example: 1) cardiovascular disorder: history of acute myocardial infarction, or unstable angina pectoris, severe arrhythmia (multifocal premature ventricular contractions, ventricular tachycardia, ventricular fibrillation) within 6 months before screening; New York Heart Association (NYHA) Class III-IV; 2) subjects with known moderate or severe persistent asthma within 5 years before screening, or subjects with ongoing inadequtaly controlled asthma;
5. Subjects who may have active mycobacterial infection, defined as: chest X-ray (posterioranterior and lateral position) examination suggests active tuberculosis infection within 3 months prior to screening/during the screening period (if required by the ethics, tuberculosis testing will be performed according to site procedures);
6. Subjects with active hepatitis, positive hepatitis B surface antigen (HBsAg), positive hepatitis B core antibody (HBcAb) + positive hepatitis B virus (HBV) deoxyribonucleic acid (DNA), or positive hepatitis C virus (HCV) antibody at screening;
7. A history of human immunodeficiency virus (HIV) infection, or HIV antibody positive at screening;
8. Subjects with treponema pallidum antibody positive at screening;
9. Having chronic active infection or acute infection requiring systemic treatment with antibiotics, antivirals, antiparasites, antiprotozoals or antifungals within 4 weeks prior to screening, or having superficial skin infections requiring treatment within 1 week prior to screening. Note: After the infection is cured, the patient may be re-screened once;
10. Subjects with known or suspected history of immunosuppression, including history of invasive opportunistic infections (e.g., histoplasmosis, listeriosis, coccidioidomycosis, pneumocystosis, and aspergillosis), even if the infection has recovered; or have unusual frequent, recurrent, or prolonged infections (as judged by the investigator);
11. Major surgery (craniotomy, thoracotomy, or laparotomy) or unhealed wounds, ulcers, or fractures within 4 weeks prior to the first dose of IMP, or major surgery planned during the study;
12. Subjects with malignancy within 5 years prior to screening (except for completely cured cervical carcinoma in situ and non-metastatic squamous cell or basal cell carcinoma of the skin);
13. A history of major organ transplant (e.g., heart, lung, kidney, liver) or hematopoietic stem cell/or bone marrow transplant;
14. Subjects who received live or live attenuated vaccines within 4 weeks prior to the first dose of IMP or plan to receive the above during the study;
15. Subjects who have participated in any clinical trial within 4 weeks before the first dose of IMP or who are within 5 half-lives of the investigational product in the previous clinical trial (whichever is longer);
16. Targeted drug therapy: received rituximab within 180 days before the first dose of IMP; received any drug therapy targeting T or B lymphocytes, cytokines, or receptors (e.g., belimumab, telitacicept, abatacept, etc.) within 180 days before the first dose of IMP; received JAK inhibitor therapy within 30 days before the first dose of IMP; or within 5 half-lives after discontinuation of the above drugs (whichever is longer);
17. Received intravenous cyclophosphamide within 180 days before the first dose of IMP or oral cyclophosphamide within 30 days before the first dose of IMP;
18. Used intravenous immunoglobulins (IVIG), prednisone ≥ 100 mg/d or equivalent glucocorticoids, or undergone plasmapheresis within 28 days before the first dose of IMP;
19. Used IL-2, thalidomide, Tripterygium wilfordii Hook F, or drug products containing Tripterygium wilfordii Hook F within 28 days before the first dose of IMP;
20. Known allergy to monoclonal antibodies or to excipients of CM313;
21. Patients with depression or tendance for suicide;
22. A history of large alcohol consumption [i.e., more than 14 units of alcohol per week (1 unit = 360 mL of beer or 45 mL of liquor containing 40% alcohol or 150 mL of wine] or a history of drug abuse

within 3 months prior to screening;

23. Female subjects who are pregnant or lactating, or planning to become pregnant or breastfeeding during the study; males whose partners plan to become pregnant during the study;
24. Any reason that, in the opinion of the investigator, contraindicates the subject's participation.

**Number of Subjects**

This study will enroll 40 subjects with SLE in a total of 4 dose levels: 2 mg/kg, 4 mg/kg, 8 mg/kg, and 16 mg/kg. Ten subjects will be enrolled at each dose level and randomized in a 4:1 ratio to receive CM313 (8 subjects) or placebo (2 subjects) at the corresponding dose.

**Study Treatment**

**Test drug:** CM313 Injection. Strength: 100 mg (5 mL)/vial. Storage conditions: 2-8°C, protected from light. Provided by Keymed Biosciences (Chengdu) Co., Ltd.

**Control drug:** Placebo. Strength: 5 mL/vial. Storage conditions: 2-8°C, protected from light. Provided by Keymed Biosciences (Chengdu) Co., Ltd.

**Dosing Regimen:**

**Dose levels and frequency:** This study will include 4 dose levels: 2 mg/kg, 4 mg/kg, 8 mg/kg, and 16 mg/kg, with dose escalation from the lowest to the highest dose level. Subjects will receive the corresponding dose of CM313 or an equal volume of placebo according to the assigned dose level and frequency. Subjects will be observed for 28 days after the first dose (single-dose phase), followed by a multiple-dose phase with QW dosing for a total of 4 doses. The 16 mg/kg dose level is optional. After all subjects in the 8 mg/kg dose level have completed the single-dose safety observation period, the sponsor and the investigator will decide whether to proceed with the 16 mg/kg dose level based on the safety data.

**Route of administration:** Both CM313 and placebo will be administered by intravenous infusion.

**Mode of administration:** The IMP will be administered by intravenous infusion after dilution and preparation (refer to the "CM313 Injection Pharmacy Manual" for details). The initial infusion rate is recommended to be 50 mL/hour, then increasing to 75 mL/hour at 60 to 90 minutes and 100 mL/hour at 90 to 120 minutes. After 120 minutes, if the subject does not experience any infusion-related reactions (IRRs), the rate may be increased by 50 mL/hour, up to the maximum allowable infusion rate of 200 mL/hour as specified in the protocol. Subjects will be closely monitored during the infusion, and if an IRR of any grade occurs, the infusion should be interrupted immediately and symptomatic treatment should be considered. To reduce the risk of IRRs, the following medications are recommended for each subject, before and after each IMP infusion:

1) Pre-infusion medications (within 1-3 hours before infusion):
   - Corticosteroids (intermediate-acting or long-acting): It is recommended to administer 100 mg methylprednisolone (or equivalent drug) intravenously before the 1st and 2nd IMP administrations; starting from the 3rd IMP administration, the corticosteroid dose can be reduced to 60 mg methylprednisolone (or equivalent drug), orally or intravenously.If patients receive the aforementioned methylprednisolone (or equivalent drug) as pre-infusion medication on the IMP administration day, they should not receive additional corticosteroids as part of their standard SLE treatment.
   - Paracetamol, 650 mg to 1000 mg, oral.
   - Antihistamines: Diphenhydramine, 25 mg to 50 mg (or equivalent), oral or intramuscular.
2) Post-infusion medications:
   - Starting from the day after the completion of the infusion, subjects should receive corticosteroids for 2 consecutive days (20 mg methylprednisolone or equivalent drug daily). If the oral corticosteroid dose in the subject's standard SLE treatment is ≥ the above dose, no additional corticosteroids will be given; if the oral corticosteroid dose in the subject's standard SLE treatment is < the above dose, corticosteroids will be supplemented to the above dose).
   - In addition, for subjects with a history of chronic obstructive pulmonary disease (COPD), post-infusion medications should be considered, including short-acting and long-acting bronchodilators and inhaled corticosteroids.

**Standard of Care:**

| |
|---|
| All subjects will maintain a stable standard of care regimen for at least 30 days before the first dose of IMP and will continue the standard of care regimen during the trial. The standard of care regimen refers to the use of any of the following (alone or in combination): corticosteroids, antimalarials, NSAIDs, immunosuppressants or immunomodulators [including azathioprine, mycophenolate (including mycophenolate mofetil, mycophenolic sodium), methotrexate, leflunomide, tacrolimus, ciclosporin].If the subject's condition changes, the standard of care regimen is allowed to be adjusted as needed per the clinical condition, and the reason for adjusting the treatment regimen needs to be recorded. |
| **Study Evaluation Measures:**<br>**Safety Evaluation**<br>Safety endpoints include: AEs, laboratory tests, physical examinations, vital signs, 12-lead ECG examinations, etc.<br>All subjects who have signed the ICF will undergo safety assessments, and AEs will be recorded as mild, moderate, or severe in terms of severity.<br>All AEs occurring from signing the ICF until the end of follow-up will be collected throughout the study and physical examinations, vital signs measurements, 12-lead ECGs, and laboratory tests will be performed according to the Schedule of Activities.<br>**Pharmacokinetic Evaluation:**<br>The PK endpoints include: PK parameters after the first dose ($T_{max}$, $C_{max}$, $AUC_{tau}$, $AUC_{0-t}$, etc.), and PK parameters after the last dose ($T_{max}$, $C_{max}$, $AUC_{tau}$, $AUC_{0-t}$, $AUC_{0-\infty}$, $T_{1/2z}$, CL, $V_z$, $MRT_{0-t}$, $R_{ac}$, etc.).<br>Blood samples will be collected for PK analysis. The sampling schedule is shown in Table 2.<br>**Pharmacodynamic Evaluation:**<br>The PD endpoints include immune cell typing (T lymphocytes, B lymphocytes, pDC cells, and NK cells) and the expression of type I IFN-related genes.<br>Blood samples will be collected for the analysis of the aforementioned PD endpoints. The sampling schedule can be found in Table 2.<br>**Immunogenicity Evaluation:**<br>The immunogenicity endpoint is ADA.<br>Blood samples will be collected to assess ADA production. The sampling schedule is shown in Table 2.<br>**Preliminary Efficacy Assessment:**<br>The efficacy endpoints include SELENA-SLEDAI score, PGA score, BILAG-2004 score, time from randomization to the first SLE flare, prednisone dosage, 24-hour urine protein quantification (only for subjects whose urine protein/creatinine ratio exceeds the ULN during the screening period), urine protein/creatinine ratio (only for subjects whose urine protein/creatinine ratio exceeds the ULN during the screening period), immunology-related endpoints, CRP, and ESR. |
| **Statistical Considerations**<br>**Efficacy Evaluation**<br>Descriptive statistics will be performed on the SELENA-SLEDAI score, PGA score, prednisone dosage, BILAG-2004 score, 24-hour urine protein quantification (only for subjects with urine protein/creatinine ratio exceeding the ULN during screening), urine protein/creatinine ratio (only for subjects with urine protein/creatinine ratio exceeding the ULN during screening), and their changes from baseline, as well as immunology-related endpoints, CRP, ESR, and their percentage changes from baseline at each visit after treatment for each dose level.<br>The Kaplan-Meier method will be used to estimate the first-flare rate of SLE in each group at different time points, as well as the 25th percentile, median, and 75th percentile of the time from randomization to the first flare of SLE, along with their respective 95% confidence intervals (Brookmeyer-Crowley method).<br>**Pharmacokinetic Evaluation**<br>Descriptive statistics will be performed on serum concentrations at each dose level according to the scheduled sampling time points.<br>PK parameters after the first dose and the last dose will be calculated using a non-compartmental model. Descriptive statistics will be performed for each PK parameter at each dose level. |

The dose linearity of $C_{max}$, $AUC_{0-t}$, $AUC_{tau}$, and $AUC_{0-\infty}$ (for the last dose only) will be assessed using the Power Model for the first dose and the last dose, respectively. A linear regression model will be fitted using log-transformed PK parameters as dependent variables and log-transformed doses as independent variables, and the slope and its 90% confidence interval (CI) will be estimated.

**Pharmacodynamic Evaluation**

Descriptive statistics will be performed for PD endpoints at each visit and their changes and percentage changes from baseline at each dose level.

**Immunogenicity Evaluation**

Descriptive statistics will be performed for the ADA results at each visit for each dose level.

**Safety Evaluation**

AEs will be coded using the latest version of the Medical Dictionary for Regulatory Activities (MedDRA) and classified by SOC and preferred term (PT). This study will mainly focus on treatment-emergent adverse events (TEAEs, defined as any AE that newly appears after treatment, or any AE that is present before treatment but worsens in severity after treatment).

The number of events, number of subjects, and percentage of the following AEs will be summarized by SOC and PT, respectively, for each dose level: all TEAEs, IMP-related TEAEs, severe TEAEs, severe IMP-related TEAEs, SAEs, IMP-related SAEs, TEAEs leading to treatment discontinuation, IMP-related TEAEs leading to treatment discontinuation, TEAEs leading to early withdrawal, IMP-related TEAEs leading to early withdrawal, TEAEs leading to death, IMP-related TEAEs leading to death, etc.

Descriptive statistics will be performed for changes from baseline in vital signs, physical examination, laboratory tests, and 12-lead ECG after treatment at each dose level.

**Version Date:** 18 Jul 2022

## Table 1 Schedule of Activities

| **Study Period►** | **Screening Period** | **Treatment Period** | | | | | | | | | **Follow-up Period** | | | |
|---|---|---|---|---|---|---|---|---|---|---|---|---|---|---|
| **Visit►** | **Screening** | **Baseline** | | | | | | | | **EOT[1]** | | | | **EOS[1]** |
| | **V1** | **V2** | **V3** | **V4** | **V5** | **V6** | **V7** | **V8** | **V9** | **V10** | **V11** | **V12** | **V13** | **V14** |
| **Study Day►** | **D-28 to D-1** | **D1[22]** | **D8** | **D15** | **D22** | **D29** | **D36** | **D43** | **D50** | **D57 or last dose +7d** | **D71 or last dose + 21d** | **D85 or last dose + 35d** | **D99 or last dose + 49d** | **D113 or last dose + 63d** |
| **Time Window►** | | **/** | **±1d** | **±1d** | **±1d** | **±1d** | **±1d** | **±1d** | **±1d** | **±1d** | **±3d** | **±3d** | **±3d** | **±3d** |
| Informed consent | X | | | | | | | | | | | | | |
| Eligibility verification | X | X[23] | | | | | | | | | | | | |
| Demographics[2] | X | | | | | | | | | | | | | |
| Medical, medication, and surgical history[3] | X | | | | | | | | | | | | | |
| Body height/weight (BMI)[4] | X | X | | | | X | X | X | X | X | | X | | X |
| Infectious disease screening[5] | X | | | | | | | | | | | | | |
| Chest X-ray | X | | | | | | | | | | | | | X |
| Antinuclear antibody[6] | X | X | | | | | | | | | | | | X |
| Blood type and IAT[7] | X | | | | | | | | | | | | | |
| Randomization | | X | | | | | | | | | | | | |
| IMP administration[8] | | X | | | | X | X | X | X | | | | | |
| Collection of prednisone dosage | X | X | X | X | X | X | X | X | X | X | X | X | X | X |
| Dispensing/collection of subject diary cards | | X | X | X | X | X | X | X | X | X | X | X | X | X |

| **Study Period▶** | **Screening Period** | **Treatment Period** | | | | | | | | | **Follow-up Period** | | | |
|---|---|---|---|---|---|---|---|---|---|---|---|---|---|---|
| **Visit▶** | **Screening** | **Baseline** | | | | | | | | **EOT[1]** | | | | **EOS[1]** |
| | **V1** | **V2** | **V3** | **V4** | **V5** | **V6** | **V7** | **V8** | **V9** | **V10** | **V11** | **V12** | **V13** | **V14** |
| **Study Day▶** | **D-28 to D-1** | **D1[22]** | **D8** | **D15** | **D22** | **D29** | **D36** | **D43** | **D50** | **D57 or last dose +7d** | **D71 or last dose + 21d** | **D85 or last dose + 35d** | **D99 or last dose + 49d** | **D113 or last dose + 63d** |
| **Time Window▶** | | **/** | **±1d** | **±1d** | **±1d** | **±1d** | **±1d** | **±1d** | **±1d** | **±1d** | **±3d** | **±3d** | **±3d** | **±3d** |
| Recording of concomitant therapies[9] | X | | | | | | | | | | | | | |
| **Safety Evaluation** | | | | | | | | | | | | | | |
| AEs | X | | | | | | | | | | | | | |
| Vital signs[10] | X | X | X | X | X | X | X | X | X | X | X | X | X | X |
| Physical examination[11] | X | X | X | X | X | X | X | X | X | X | X | X | X | X |
| 12-lead ECG[12] | X | X | X | X | X | X | X | X | X | X | X | X | X | X |
| Hematology[13] | X | X | X | X | X | X | X | X | X | X | X | X | X | X |
| Blood chemistry[14] | X | X | X | X | X | X | X | X | X | X | X | X | X | X |
| Coagulation function[15] | X | X | X | X | X | X | X | X | X | X | X | X | X | X |
| Urinalysis[16] | X | X | X | X | X | X | X | X | X | X | X | X | X | X |
| Pregnancy test[17] | X | X | | | | X | | | | X | | X | | X |
| **Biological Sample Collection** | | | | | | | | | | | | | | |
| PK blood sample collection | See "Pharmacokinetic, Pharmacodynamic, and Immunogenicity Blood Sampling Schedule" | | | | | | | | | | | | | |
| PD blood sample collection | See "Pharmacokinetic, Pharmacodynamic, and Immunogenicity Blood Sampling Schedule" | | | | | | | | | | | | | |
| Immunogenicity blood sample collection | See "Pharmacokinetic, Pharmacodynamic, and Immunogenicity Blood Sampling Schedule" | | | | | | | | | | | | | |
| **Efficacy Evaluation** | | | | | | | | | | | | | | |

| **Study Period►** | **Screening Period** | **Treatment Period** | | | | | | | | | **Follow-up Period** | | | |
|---|---|---|---|---|---|---|---|---|---|---|---|---|---|---|
| **Visit►** | **Screening** | **Baseline** | | | | | | | | **EOT[1]** | | | | **EOS[1]** |
| | **V1** | **V2** | **V3** | **V4** | **V5** | **V6** | **V7** | **V8** | **V9** | **V10** | **V11** | **V12** | **V13** | **V14** |
| **Study Day►** | **D-28 to D-1** | **D1[22]** | **D8** | **D15** | **D22** | **D29** | **D36** | **D43** | **D50** | **D57 or last dose +7d** | **D71 or last dose + 21d** | **D85 or last dose + 35d** | **D99 or last dose + 49d** | **D113 or last dose + 63d** |
| **Time Window►** | | **/** | **±1d** | **±1d** | **±1d** | **±1d** | **±1d** | **±1d** | **±1d** | **±1d** | **±3d** | **±3d** | **±3d** | **±3d** |
| 24-hour urine protein[18] | X | X | | X | | X | | X | | X | X | X | X | X |
| Urine protein/creatinine ratio[18] | X | X | | X | | X | | X | | X | X | X | X | X |
| Immunology-related endpoints[19] | X | X | | X | | X | | X | | X | X | X | X | X |
| CRP, ESR[20] | X | X | | X | | X | | X | | X | X | X | X | X |
| SELENA-SLEDAI score | X | X | | X | | X | | X | | X | X | X | X | X |
| SLE flare assessment[21] | | X | | X | | X | | X | | X | X | X | X | X |
| BILAG-2004 score | | X | | | | X | | | | X | | X | | X |
| PGA score | X | X | | X | | X | | X | | X | X | X | X | X |

Abbreviations: BMI=body mass index; CRP=C-reactive protein; ECG=electrocardiogram; EOT=end of treatment; EOS=end of study; ESR=erythrocyte sedimentation rate; IAT=indirect antiglobulin test; SLE=systemic lupus erythematosus; PGA= Physician Global Assessment.

Notes:

1. If a subject discontinues treatment early but does not withdraw from the study, the EOT visit and subsequent follow-up visits should be completed as far as possible. For subjects who withdraw early from the study, an early withdrawal visit will be completed. The required procedures, examinations, and assessments will be the same as those conducted at the EOS visit. The sample collection time or safety examination time should be close to the time of withdrawal from the study as far as possible. At the "early withdrawal" visit, procedures, examinations and assessments will be waived if there are results within 3 days.
2. For demographics, the age, sex, ethnicity, smoking history, alcohol consumption and menstrual history of the subjects should be recorded.
3. Medical, medication and surgical history: ① History of SLE: The date of diagnosis of SLE should be recorded. ② SLE medication history: The medication use (including specific drugs/treatment, dosage, and usage, etc.) for at least 2 years prior to the screening visit should be recorded. ③ Medical

history of other diseases: The occurrence of other diseases (including disease name and time of onset, etc.) within at least 6 months prior to the screening visit should be recorded. ④ Previous treatment history of other diseases: The treatment (including specific drugs/treatment, usage and dosage, etc.) within at least 1 month prior to the screening visit should be recorded. ⑤ Previous surgical history: The surgery (including name and date of surgery, etc.) within at least 6 months prior to the screening visit should be recorded.

4. Body height/weight: Body height (cm) and weight (kg) will be measured at screening (D-28 to D-1), and BMI will be calculated based on body height and weight. Only weight will be measured at V2, V6, V7, V8, V9, V12, EOT visit, and EOS visit.
5. Infectious disease screening:
    a) Hepatitis B screening: Hepatitis B five items (hepatitis B virus surface antigen [HBsAg], hepatitis B virus surface antibody [HBsAb], hepatitis B virus core antibody [HBcAb], hepatitis B virus e antigen [HBeAg] and hepatitis B virus e antibody [HBeAb]); hepatitis B virus deoxyribonucleic acid (HBV-DNA) test may be added if necessary. HbsAg-positive subjects as well as subjects with both HbcAb-positive and HBV-DNA-positive results need to be excluded from the study;
    b) Hepatitis C screening: Hepatitis C virus (HCV) antibody, and HCV-ribonucleic acid (HCV-RNA) test may be added if necessary;
    c) HIV screening: HIV antibody;
    d) Tuberculosis screening: Chest X-ray (posteroanterior and lateral), and additional TB screening-related tests may be added according to the site's diagnostic and treatment routine;
    e) Syphilis screening: treponema pallidum antibody test.
6. Antinuclear antibody (ANA): At the baseline visit, ANA results within 3 days prior to the first dose will be acceptable.
7. Blood type and IAT: Blood type and IAT, including ABO, Rh, and IAT, must be performed during the screening period, and subjects will be provided with an identification card indicating their blood type and IAT information.
8. IMP administration: The dose will be calculated based on the subject's weight before each administration. The IMP will be administered by intravenous infusion after dilution and preparation. The infusion rate needs to be controlled, and drugs such as glucocorticoids will be given before and after the infusion to prevent infusion-related reactions.
9. Concomitant therapies: including drug therapy and non-drug therapy.
10. Vital signs: including temperature, pulse, respiration, blood pressure. Subjects should rest for at least 5 min prior to examination.
11. Physical examination: including head and face, eyes, ears, nose and throat, oral cavity, skin, lymph nodes, respiratory system, cardiovascular system, abdomen, genitourinary system (if necessary), musculoskeletal system, nervous system, and mental status.
12. 12-lead ECG: including heart rate, PR interval, QRS duration, QT interval, and QTcF. If blood samples need to be collected on the day of 12-lead ECG, the 12-lead ECG should be performed before blood collection or at least 20 minutes after the completion of blood collection. Subjects should rest for at least 5 min prior to examination.
13. Hematology: including red blood cells, hemoglobin, white blood cells, hematocrit, platelets, neutrophil count and percentage, lymphocyte count and percentage, monocyte count and percentage. Hematology results within 3 days prior to the first dose will be acceptable at the baseline visit.
14. Blood chemistry: including glucose, total cholesterol, low density lipoprotein cholesterol, high density lipoprotein cholesterol, alanine aminotransferase, aspartate aminotransferase, total protein, albumin, total bilirubin, direct bilirubin, lactate dehydrogenase, alkaline phosphatase, glutamyl transpeptidase, creatinine, urea/urea nitrogen, uric acid, sodium, potassium, chloride, calcium. Serum chemistry results within 3 days prior to the first dose will be acceptable at the baseline visit.

15. Coagulation: including prothrombin time, activated partial thromboplastin time, thrombin time, fibrinogen and international normalized ratio. Coagulation results within 3 days prior to the first dose will be acceptable at the baseline visit.
16. Urinalysis: including urine protein, glucose, ketones, occult blood, microscopic examination (including red blood cells/high power field, white blood cells/high power field, poikilocytes, casts and specific classification), urobilinogen, urine bilirubin, nitrite, urine specific gravity, and acidity or alkalinity. Urinalysis results within 3 days prior to the first dose will be acceptable at the baseline visit. If the subject is menstruating, the urinalysis may be postponed until after the menstrual period.
17. Pregnancy test: for women of childbearing potential only. Blood pregnancy tests will be performed at the screening visit and the EOS visit, while blood or urine pregnancy tests will be performed at other visits. Unscheduled blood or urine pregnancy tests may be performed at the investigator's discretion during the course of the study.
18. 24-hour urine protein will only be conducted for subjects whose urine protein/creatinine ratio exceeds the ULN during the screening period. The 24-hour urine protein and urine protein/creatinine ratio at the baseline visit can be completed on D1 or within 48 hours before D1. The 24-hour urine protein and urine protein/creatinine ratio within 3 days prior to the first dose are acceptable at the baseline visit.
19. Immunology-related endpoints: including IgG, IgA, IgM, IgE, complement (C3, C4), and anti-ds-DNA antibody. These will be tested by the laboratories of each study site during the screening period. Baseline and subsequent examinations will be conducted by the central laboratory. Among them, the results of IgG, IgA, IgM, and IgE at baseline and thereafter will not be provided to each study site before unblinding to avoid breaking of blindness.
20. CRP and ESR results within 3 days prior to the first dose will be acceptable. Baseline examinations can be completed on D1 or within 48 hours before D1.
21. SLE flare assessment: The number of SLE flares, the severity of each flare, and the specific date of each flare need to be recorded. The severity of the flare refers to the SLE Flare Scoring Index (see Appendix 3).
22. Safety laboratory tests before the first dose, including pregnancy tests, can be completed on D1 or within 48 hours before D1.
23. Laboratory test results from the screening period can be used for the eligibility verification based on inclusion/exclusion criteria.

**Table 2 Pharmacokinetic, Pharmacodynamic, and Immunogenicity Blood Sampling Schedule**

| | PK blood sample collection | PD blood sample collection (immune cell typing) | PD blood sample collection (IFN-I related gene expression) | Immunogenicity blood sample collection |
|---|---|---|---|---|
| **First dose** | Within 2 hours before the start of infusion, 3 hours (±15 min) after the start of infusion, at the end of infusion (within 5 min), and at 2 hours (±15 min), 5 hours (±30 min), 24 hours (±2 h), 72 hours (±4 h), 168 hours (±24 h), 336 hours (±24 h), 504 hours (±72 h) after the end of infusion | Within 2 hours before the start of infusion, and at 24 hours (± 2 hours), 168 hours (± 24 hours), 336 hours (± 24 hours), and 504 hours (± 72 hours) after the end of infusion | Within 2 hours before the start of infusion and at 168 hours (±24 hours) after the end of infusion | Within 2 hours before the start of infusion |
| **Second dose** | Within 2 hours before the start of infusion | Within 2 hours before the start of infusion and at 24 hours (±2 hours) after the end of infusion | Within 2 hours before the start of infusion | Within 2 hours before the start of infusion |
| **Third dose** | Within 2 hours before the start of infusion | NA | NA | NA |
| **Fourth dose** | Within 2 hours before the start of infusion | NA | NA | NA |
| **Last dose** | Within 2 hours before the start of infusion, 3 hours (±15 min) after the start of infusion, at the end of infusion (within 5 min), and at 2 hours (±15 min), 5 hours (±30 min), 24 hours (±2 h), 72 hours (±4 h), 168 hours (±24 h), 504 hours (±72 h), 840 hours (±72 h), 1176 hours (±72 h), and 1512 hours (±72 h) after the end of infusion | Within 2 hours before the start of infusion, and at 24 hours (± 2 h), 840 hours (± 72 h), and 1512 hours (± 72 h) after the end of infusion | Within 2 hours before the start of infusion and at 840 hours (± 72 h) and 1512 hours (± 72 h) after the end of infusion | Within 2 hours before the start of infusion and at 840 hours (± 72 h) and 1512 hours (± 72 h) after the end of infusion |
| At early withdrawal (if applicable) | | | | |

# Table of Contents

**SUMMARY OF CHANGES IN THE PROTOCOL ........ 2**

**PROTOCOL SYNOPSIS ........ 4**

**TABLE 1 SCHEDULE OF ACTIVITIES ........ 13**

**TABLE 2 PHARMACOKINETIC, PHARMACODYNAMIC, AND IMMUNOGENICITY BLOOD SAMPLING SCHEDULE ........ 18**

**TABLE OF CONTENTS ........ 19**

**LIST OF ABBREVIATIONS AND DEFINITION OF TERMS ........ 24**

**1. INTRODUCTION ........ 27**

1.1. DISEASE BACKGROUND AND AVAILABLE THERAPIES ........ 27

1.2. BACKGROUND FOR THE TREATMENT TARGET ........ 29

*1.2.1. CD38 as a Potential Target for SLE Treatment ........ 30*

*1.2.2. Current Status of Drug Development for SLE Targeting CD38 ........ 30*

1.3. INTRODUCTION OF THE TEST DRUG ........ 31

*1.3.1. Nonclinical Studies ........ 32*

*1.3.2. Clinical Studies ........ 32*

**2. STUDY OBJECTIVES AND STUDY ENDPOINTS ........ 35**

**3. STUDY PLAN ........ 36**

3.1. OVERALL STUDY DESIGN AND PLAN ........ 36

3.2. RATIONALE FOR STUDY DESIGN ........ 38

3.3. RAIONALE FOR DOSE SELECTION ........ 38

3.4. BENEFIT/RISK ASSESSMENT ........ 40

3.5. NUMBER OF SUBJECTS ........ 42

3.6. STUDY PERIOD ........ 42

3.7. SELECTION OF STUDY POPULATION ........ 42

*3.7.1. Inclusion Criteria ........ 42*

*3.7.2. Exclusion Criteria ........ 43*

*3.7.3. Subject Re-screening ........ 46*

*3.7.4. Criteria for Withdrawal of Subject ........ 46*

3.8. STUDY TREATMENT ........ 47

*3.8.1. Investigational Medicinal Products ........ 47*

*3.8.2. Mode of Administration ........ 47*

*3.8.3. Randomization and Blinding ........ 50*

*3.8.4. Packaging and Labeling of Investigational Medicinal Products ........ 51*

*3.8.5. Storage and Management of Investigational Medicinal Products* .......... *52*
*3.8.6. Dose Escalation Interruption and Stopping Criteria* .......... *52*
*3.8.7. Premature Discontinuation of Treatment* .......... *53*
*3.8.8. Concomitant Therapies* .......... *54*
*3.8.9. Medication Compliance* .......... *57*
3.9. ENDPOINTS .......... 57
*3.9.1. Safety Endpoints* .......... *57*
*3.9.2. Pharmacokinetic Endpoints* .......... *58*
*3.9.3. Pharmacodynamic Endpoints* .......... *58*
*3.9.4. Immunogenicity Endpoints* .......... *58*
*3.9.5. Efficacy Endpoints* .......... *58*
3.10. END OF STUDY .......... 58
3.11. ACTIONS IN CASE OF STUDY INTERRUPTION DUE TO NATURAL DISASTERS OR PUBLIC HEALTH EVENTS .......... 59
**4. STUDY PROCEDURES .......... 60**
4.1. STUDY STEPS .......... 60
*4.1.1. V1 (D-28 to D-1, Screening Period)* .......... *60*
*4.1.2. Treatment Period (D1 to D57)* .......... *61*
*4.1.3. Follow-up Period (D58 to D113)* .......... *68*
4.2. DOSE INTERRUPTION .......... 71
4.3. EARLY WITHDRAWAL FROM THE STUDY .......... 71
4.4. UNSCHEDULED VISITS .......... 71
4.5. STUDY TERMINATION CRITERIA .......... 71
4.6. PREGNANCY/LACTATION AND CONTRACEPTIVE REQUIREMENTS .......... 72
**5. METHODS OF ASSESSMENT .......... 73**
5.1. ROUTINE ASSESSMENTS .......... 73
5.2. SAFETY ASSESSMENTS .......... 73
*5.2.1. Physical Examination* .......... *74*
*5.2.2. Vital Signs* .......... *74*
*5.2.3. 12-lead ECG* .......... *74*
*5.2.4. Laboratory Tests* .......... *75*
*5.2.5. Adverse Events* .......... *75*
*5.2.6. Pregnancy Test* .......... *75*
5.3. EFFICACY EVALUATION .......... 75
*5.3.1. SELENA-SLEDAI Score* .......... *75*

*5.3.2. SLE Flare/BILAG-2004/PGA Score* ........ *76*
*5.3.3. Urine Protein/Creatinine Ratio, 24-hour Urine Protein* ........ *76*
*5.3.4. Prednisone Dosage* ........ *77*
*5.3.5. IgG, IgA, IgM, IgE, Anti-ds-DNA Antibody, Complement (C3, C4), CRP, ESR* ........ *77*
5.4. PHARMACOKINETIC EVALUATION ........ 77
*5.4.1. Pharmacokinetic Sample Collection* ........ *77*
*5.4.2. Pharmacokinetic Sample Processing* ........ *77*
5.5. PHARMACODYNAMIC EVALUATION ........ 78
*5.5.1. Biomarker Sample Collection* ........ *78*
*5.5.2. Biomarker Sample Processing* ........ *78*
5.6. IMMUNOGENICITY EVALUATION ........ 78
*5.6.1. Immunogenicity Sample Collection* ........ *78*
*5.6.2. Immunogenicity Sample Processing* ........ *78*
**6. SAFETY ASSESSMENTS ........ 79**
6.1. ADVERSE EVENTS ........ 79
*6.1.1. Definition of Adverse Event* ........ *79*
*6.1.2. Recording of Adverse Events* ........ *79*
*6.1.3. Severity* ........ *79*
*6.1.4. Causality* ........ *80*
6.2. SERIOUS ADVERSE EVENTS ........ 80
*6.2.1. Definition of Serious Adverse Event* ........ *80*
*6.2.2. Reporting of Serious Adverse Events* ........ *81*
6.3. OTHER SAFETY INFORMATION ........ 82
*6.3.1. Abnormal Laboratory Results* ........ *82*
*6.3.2. Overdose* ........ *82*
*6.3.3. Pregnancy* ........ *82*
**7. DATA MANAGEMENT ........ 84**
7.1. DATA COMPLETION REQUIREMENTS FOR INVESTIGATORS ........ 84
7.2. DATA MONITORING REQUIREMENTS FOR MONITORS ........ 84
7.3. DATA MANAGEMENT REQUIREMENTS ........ 84
*7.3.1. Case Report Form Design* ........ *84*
*7.3.2. Database Design* ........ *85*
*7.3.3. Electronic Case Report Form Completion Guidelines* ........ *85*
*7.3.4. Case Report Form Completion* ........ *85*

*7.3.5. Data Review and Query Management* ...... *85*
*7.3.6. Medical Coding* ...... *86*
*7.3.7. Consistency Comparison of Serious Adverse Events* ...... *86*
*7.3.8. Data Review Meetings* ...... *86*
*7.3.9. Database Lock and Unlock* ...... *86*
**8. STATISTICAL CONSIDERATIONS ...... 87**
8.1. SAMPLE SIZE ESTIMATION ...... 87
8.2. ANALYSIS SETS ...... 87
8.3. STATISTICAL ANALYSES ...... 88
*8.3.1. General Principles* ...... *88*
*8.3.2. Subject Disposition* ...... *88*
*8.3.3. Demographics and Baseline Characteristics* ...... *88*
*8.3.4. Efficacy Evaluation* ...... *88*
*8.3.5. Pharmacokinetic Evaluation* ...... *89*
*8.3.6. Pharmacodynamic Evaluation* ...... *89*
*8.3.7. Immunogenicity Evaluation* ...... *89*
*8.3.8. Safety Evaluation* ...... *89*
*8.3.9. Subgroup Analysis* ...... *91*
*8.3.10. Interim Analysis* ...... *91*
*8.3.11. Multiplicity Issues* ...... *91*
*8.3.12. Handling of Missing Data* ...... *91*
**9. QUALITY CONTROL AND ASSURANCE ...... 91**
9.1. MONITORING ...... 91
9.2. AUDIT AND INSPECTION ...... 91
9.3. QUALITY CONTROL ...... 92
**10. ETHICS ...... 93**
10.1. ETHICS COMMITTEE ...... 93
10.2. ETHICAL GUIDANCE FOR THE STUDY ...... 93
10.3. INFORMED CONSENT OF THE SUBJECTS ...... 93
**11. STUDY MANAGEMENT AND MATERIALS ...... 94**
11.1. ELECTRONIC CASE REPORT FORM ...... 94
11.2. DATA COLLECTION ...... 94
11.3. STORAGE OF SOURCE DOCUMENTS ...... 95
11.4. STUDY DATA RECORDS ...... 95
11.5. CONFIDENTIALITY ...... 96

11.6. REVIEW BY THE ETHICS COMMITTEE ........ 96
11.7. PROTOCOL AMENDMENTS ........ 96
11.8. PROTOCOL COMPLIANCE AND PROTOCOL DEVIATIONS ........ 97
11.9. PUBLICATION OF STUDY RESULTS ........ 97
11.10. CLINICAL STUDY REPORT ........ 97
11.11. INSURANCE, INDEMNITY, AND COMPENSATION ........ 98
11.12. STUDY TERMINATION ........ 98
11.13. DOCUMENT MANAGEMENT AT STUDY SITE ........ 98

**12. REFERENCES ........ 99**

**13. APPENDIXES ........ 101**

APPENDIX 1 2019 EULAR/ACR CLASSIFICATION CRITERIA FOR SLE ........ 101
APPENDIX 2 SELENA-SLEDAI SCORE ........ 102
APPENDIX 3 SLE FLARE SCORING INDEX ........ 104
APPENDIX 4 BILAG-2004 SCORE ........ 105
APPENDIX 5 PGA SCORE ........ 108
APPENDIX 6 CONTRACEPTIVE MEASURES, DEFINITION OF WOMEN OF CHILDBEARING POTENTIAL, AND CONTRACEPTIVE REQUIREMENTS ........ 109

# List of Abbreviations and Definition of Terms

| **Abbreviation** | **Definition** |
|---|---|
| ACR | American College of Rheumatology |
| ADA | Anti-drug antibody |
| ADCC | Antibody dependence cell-mediated cytotoxicity |
| ADCP | Antibody-dependence cell-mediated phagocytosis |
| ADPR | Adenosine diphosphate ribose |
| AE | Adverse event |
| ALT | Alanine aminotransferase |
| ANA | Antinuclear antibody |
| AST | Aspartate aminotransferase |
| $AUC_{0-t}$ | Area under the serum concentration-time curve from time 0 to time t |
| $AUC_{0-\infty}$ | Area under the serumconcentration-time curve from time 0 to time ∞ |
| $AUC_{tau}$ | Area under the serum concentration-time curve over a dosing interval |
| BMI | Body mass index |
| BUN | Urea nitrogen |
| cADPR | Cyclic adenosine diphosphate ribose |
| CDC | Complement dependent cytoxicity |
| CL | Clearance |
| $C_{max}$ | Maximum concentration |
| COPD | Chronic obstructive pulmonary disease |
| Cr | Creatinine |
| CRF | Case Report Form |
| CRO | Contract research organization |
| CRP | C-reactive protein |
| DLT | Dose-limiting toxicity |
| ds-DNA | Double-stranded deoxyribonucleic acid |
| EC | Ethics Committee |
| ECG | Electrocardiogram |
| eCRF | Electronic case report form |
| EDC | Electronic data capture |
| eGFR | Glomerular filtration rate |
| EMA | European Medicines Agency |
| EOT | End of treatment |
| EOS | End of study |
| ESR | Erythropenia sedimentation rate |
| EULAR | European League Against Rheumatism |
| FAS | Full Analysis Set |

| Abbreviation | Definition |
|---|---|
| FDA | Food and Drug Administration |
| GCP | Good Clinical Practice |
| HBcAb | Hepatitis B core antibody |
| HBeAb | Hepatitis B e antibody |
| HBeAg | Hepatitis B e antigen |
| HBsAb | Hepatitis B surface antibody |
| HBsAg | Hepatitis B surface antigen |
| HCV | Hepatitis C virus |
| HCV-RNA | Hepatitis C virus-ribonucleic acid |
| HIV | Human immunodeficiency virus |
| IAT | Indirect antiglobulin test |
| ICF | Informed consent form |
| IgA | Immunoglobulin A |
| IgE | Immunoglobulin E |
| IgG | Immunoglobulin G |
| IgM | Immunoglobulin M |
| IFN | Interferon |
| IRR | Infusion-related reaction |
| IMGS | Immunogenicity Set |
| IVIG | IV immunoglobulin |
| IWRS | Interactive web response system |
| NSAIDs | Nonsteroidal anti-inflammatory drugs |
| MedDRA | Medical Dictionary for Regulatory Activities |
| MR | Minimal response |
| $MRT_{0-t}$ | Mean residence time |
| NOAEL | No-observed-adverse-effect level |
| NMPA | National Medical Products Administration |
| PD | Pharmacodynamics |
| NYHA | New York Heart Association |
| PDS | PD Set |
| PGA | Physician Global Assessment |
| PK | Pharmacokinetics |
| PKCS1 | Pharmacokinetic Concentration Set for First Dose |
| PKCS2 | Pharmacokinetic Concentration Set for Last Dose |
| PKPS1 | Pharmacokinetic Parameter Set for First Dose |
| PKPS2 | Pharmacokinetic Parameter Set for Last Dose |
| PMDA | Pharmaceuticals and Medical Devices Agency |
| PPS | Per Protocol Set |

| **Abbreviation** | **Definition** |
|---|---|
| PT | Preferred term |
| QW | Once weekly |
| $R_{ac}$ | Accumulation index |
| SAE | Serious adverse event |
| SAP | Statistical Analysis Plan |
| SAS | Statistical analysis software |
| SD | Stable disease |
| SLE | Systemic lupus erythematosus |
| SOC | System organ class |
| SS | Safety Set |
| SUSAR | Suspected unexpected serious adverse reaction |
| TEAE | Treatment-emergent adverse event |
| $T_{max}$ | Time to reach the maximum concentration |
| TRAE | Treatment-related adverse event |
| $T_{1/2z}$ | Elimination half-life |
| ULN | Upper limit of normal |
| VGPR | Very good partial response |
| $V_z$ | Volume of distribution |

# 1. Introduction

## 1.1. Disease Background and Available Therapies

Systemic lupus erythematosus (SLE) is a systemic autoimmune disease characterized by a large number of autoantibodies in the body, abnormal B lymphocyte function, multi-system and multi-organ involvement throughout the body, and repeated relapses and remissions. If not treated promptly, it can cause irreversible damage to the affected organs, ultimately leading to the death of the patient. The cause of SLE is still unclear and may be related to genetic, sex hormone, and environmental factors [1]. SLE is more common in women, with a male-to-female morbidity ratio of about 1:10-12. The morbidity of SLE varies greatly geographically. In the United States, there are about 100,000 to 500,000 SLE patients, and there is also estimation that there are more than 1 million SLE patients in the United States. The reported prevalence of SLE in Europe is 25-39/100,000; the prevalence of SLE in mainland China is about 30-70/100,000 [2][3].

SLE can lead to arthritis, renal insufficiency, inflammation of the heart and lungs, central nervous system disorder, vasculitis, severe rash, and blood system diseases (including anemia, leukopenia, and thrombocytopenia). The clinical manifestations of SLE are heterogeneous and there is a lack characteristics or examinations for diagnosis, making it difficult for clinicians to diagnose SLE. In 1997, the American College of Rheumatology (ACR) established classification criteria for SLE based on SLE-related symptoms and/or signs, abnormal laboratory test results, and antinuclear antibodies (ANA). Meeting 4 of the 11 classification criteria can be diagnosed as SLE. To further improve the sensitivity and specificity of the SLE classification criteria, in 2019, the European League Against Rheumatism (EULAR) and ACR jointly launched the 2019 EULAR/ACR classification criteria for SLE based on the 1997 ACR SLE classification criteria. This standard includes 1 entry criterion, 10 domains, and 18 criteria. In each criterion, causes such as infection, neoplasm malignant, and drug need to be excluded. Those who have met a certain criterion in the past can also be scored. The highest weighted score in each domain is included in the total score. A total score ≥ 10 can be classified as SLE [1][4].

The short-term goals of SLE treatment are to control disease activity and improve clinical symptoms to achieve clinical remission or the lowest possible disease activity; the long-term goals are to prevent and reduce SLE recurrence, reduce drug adverse reactions, prevent and control organ damage caused by the disease, achieve sustained remission, reduce mortality,

and improve patients' quality of life. Currently, SLE treatment includes a variety of drugs such as glucocorticoids, antimalarials, immunosuppressants, and biologics [1]:

- **Glucocorticoids:** Glucocorticoids play a crucial role in the treatment of SLE and are the most commonly used basic drugs for inducing remission and controlling SLE, as recommended by both domestic and international guidance. The incidence of glucocorticoid-related adverse reactions is >30%. Long-term high-dose use of glucocorticoids can lead to significant adverse reactions such as osteoporosis, femoral head necrosis, metabolic disorders, increased risk of infection, oedema, weight increased, and hyperlipidemia.
- **Antimalarials:** Long-term use of hydroxychloroquine in SLE patients can reduce disease activity, lower the risk of organ injury and thrombosis, improve blood lipid levels, and increase survival rates. However, long-term use of hydroxychloroquine can lead to retinopathy, and some high-risk groups (long-term use and/or high dose of hydroxychloroquine, with liver or kidney disease, concurrent use of tamoxifen, history of retinal or macular disease, elderly, etc.) are more prone to developing retinopathy.
- **Immunosuppressants:** The use of immunosuppressants can reduce the cumulative dose of hormones and prevent disease recurrence. For refractory (poor response to conventional treatment) or recurrent SLE patients, the use of immunosuppressants can reduce the dose of hormones, control disease activity, and improve the clinical remission rate. The main adverse reactions include severe infection, teratogenicity, and increased risk of neoplasm.
- **Biologics:** For refractory (poor response to conventional treatment) or recurrent SLE patients, the use of biologics can significantly increase the complete and partial remission rates, reduce disease activity and disease recurrence rates, and reduce hormone dosage. Currently, belimumab has been approved in China. Its adverse reactions include infection, headache, and nausea. The efficacy and safety of belimumab in Chinese SLE patients need further verification, and there is still room for further improvement in its efficacy. Currently, belimumab has been approved in China. Its adverse reactions include infection, headache, and nausea. The efficacy and safety of belimumab in Chinese SLE patients need further verification, and there is still room for further improvement in its efficacy.

SLE can present with a variety of clinical courses, ranging from relatively benign disease to rapidly progressive disease with fulminant organ failure and even death. With the improvement of SLE diagnosis and treatment, the 5-year survival rate of SLE has increased significantly since the mid-20th century, from about 40% in the 1950s to over 90% in the 1980s [5]. On the

one hand, SLE has transformed from an acute, highly fatal disease in the past to a chronic, controllable disease; on the other hand, the mortality rate of SLE patients is still 2-5 times that of the general population, and the 10-year survival rate is only 70% [6],[7]. In addition, SLE patients lack safe and effective therapeutic drugs, and long-term use of glucocorticoids and immunosuppressants can lead to serious adverse reactions, affecting patients' health and quality of life. Belimumab and anifrolumab are the only two new SLE drugs approved by the US Food and Drug Administration (FDA) in the past 60 years, but half of the patients still do not respond well to these drugs [8][9]. Therefore, there are still unmet clinical needs in the treatment of SLE.

## 1.2. Background for the Treatment Target

SLE is currently believed to have a genetic susceptibility, with >10 chromosomal regions identified as potentially linked to its development. This genetic susceptibility leads to the production of pathogenic autoantibodies, cytokine abnormalities, and autoreactive B and T cells. B cell abnormalities include alterations in B cell differentiation status, manifested by an increase in the number of immunoglobulin-secreting plasma cells, which is particularly evident during active SLE disease. B cells isolated from SLE patients often exhibit dysregulated expression of cell surface molecules (such as B7 and CD40), indicating a state of enhanced B cell activation [10].

Autoantibodies have been shown to be an important and specific early biomarker of disease progression and severity in SLE and are associated with SLE disease activity. Autoantibodies participate in the disease process of SLE by binding to target antigens and, through the formation of immune complexes, trigger a cascade of cellular and cytokine responses, leading to immune injury. The initiation mechanisms of SLE-related autoantibody production remain unclear. However, prolonged B cell survival, altered self-antigen tolerability, and abnormal apoptosis may be potential triggers for autoantibody responses [11].

Following B cell activation, short-lived plasma cells, plasmablasts, and long-lived plasma cells produce high titers of autoantibodies. Long-lived plasma cells reside in the bone marrow and inflammatory tissues and can produce large amounts of autoantibodies independently of B cell activation [12]. Rituximab (anti-CD20) and belimumab (anti-BLyS) can, to some extent, prevent the proliferation of short-lived plasma cells and reduce autoantibody production. However, non-proliferative long-lived plasma cells are less susceptible to their effects, leading to continued autoantibody production and SLE disease progression [13],[14]. Therefore, the development of drugs targeting long-lived plasma cells may provide more effective treatments for SLE.

### 1.2.1. CD38 as a Potential Target for SLE Treatment

CD38 is a type II glycoprotein receptor with a short intracellular N-terminus (21 ammonia acids) lacking known signal transduction motifs and an extracellular C-terminus (258 ammonia acids) that interacts with its ligand CD31 (PECAM-1) to regulate lymphocyte migration, activation, proliferation, and B cell differentiation [15]. The extracellular domain of CD38 possesses both cyclase and hydrolase activities, catalyzing the conversion of the substrate NAD+ into cyclic adenosine diphosphate ribose (cADPR) and adenosine diphosphate ribose (ADPR), which are involved in regulating intracellular calcium release and activating lymphocyte proliferation. CD38 forms tetramers on the cell membrane, which is crucial for its enzymatic activity.

In normal tissues, CD38 is expressed on activated lymphocytes and plasma cells, with low levels also found on lymphoblasts in lymph node germinal centers, follicular cells, peripheral blood monocytes/NK cells, T cells, B cells, platelets, and red blood cells. It is not expressed in other normal tissues [15]. As a B lymphocyte development marker, CD38 expression varies across different stages of B cells: it is highly expressed on bone marrow precursor cells, downregulated in resting normal B cells, and upregulated again in terminally differentiated plasma cells. Importantly, CD38 is also highly expressed on various immunosuppression cells, such as regulatory T cells, regulatory B cells, and bone marrow-derived suppressor cells.

Many immune cells involved in the pathogenesis of SLE express CD38 or express CD38 upon stimulation. Studies have shown that compared to healthy individuals, SLE patients exhibit increased CD38 expression in different leukocyte sub-populations, such as CD4+, CD8+ memory T cells, and monocytes [16]. Due to the high expression of CD38 on the surface of plasmablasts and plasma cells that produce autoantibodies, it is considered a potential target for the treatment of autoimmune diseases driven by autoantibodies.

### 1.2.2. Current Status of Drug Development for SLE Targeting CD38

Monoclonal antibodies targeting CD38 can effectively kill autoantibody-producing plasmablasts and plasma cells. The mechanisms by which CD38 antibodies exert their cytotoxic effects on target cells primarily involve classic antibody-dependent cell-mediated cytotoxicity (ADCC), complement-dependent cytotoxicity (CDC), and antibody-dependent cell-mediated phagocytosis (ADCP) immune effects. They may also continuously promote the death of autoantibody-producing plasmablasts and plasma cells through antibody-mediated apoptosis and regulation of ADPR enzyme activity (Figure 1).

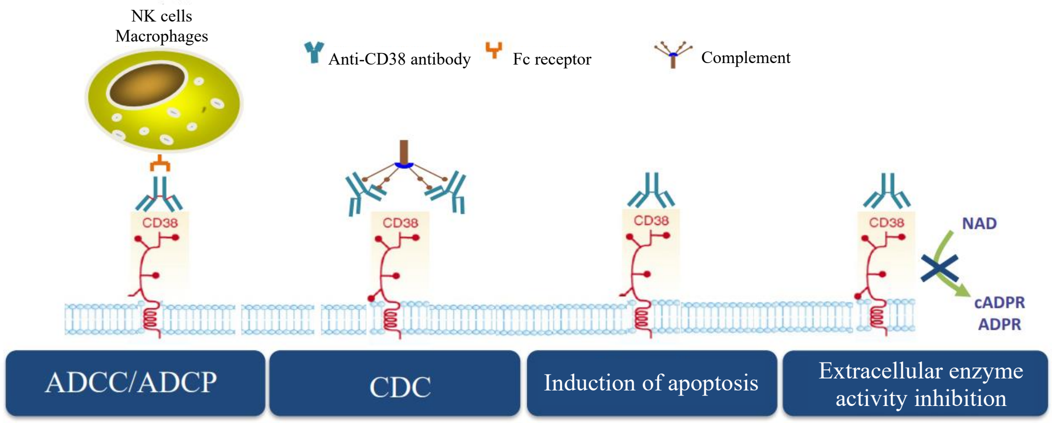


**Figure 1 Potential Mechanism of Action of Anti-CD38 Antibodies**

Daratumumab (trade name Darzalex) is a fully human IgG1 antibody drug that received accelerated approval from the FDA in November 2015 and was subsequently approved by the European Medicines Agency (EMA) in April 2017 and the Pharmaceuticals and Medical Devices Agency (PMDA) of Japan in July 2017. It is indicated for the treatment of adult patients with relapsed or refractory multiple myeloma (MM) who have received at least one prior therapy (including a protease inhibitor and an immunomodulatory agent) and whose condition aggravated after treatment. In a study published in the New England Journal of Medicine in September 2020 [17], researchers used daratumumab to treat 2 patients with refractory and life-threatening SLE. The treatment regimen consisted of daratumumab 16 mg/kg administered once weekly (QW). After 1 month of treatment, both patients showed significant clinical responses, along with the elimination of long-lived plasma cells, decreased type I interferon activity, and downregulation of activation and antigen-stimulated gene transcription in T cells. These findings provide preliminary evidence for the feasibility of using anti-CD38 monoclonal antibodies (mAbs) to treat SLE.

In summary, preliminary efficacy of anti-CD38 mAbs has been observed in patients with refractory SLE, and further clinical trials will be conducted. The efficacy of this class of drugs in MM has been confirmed and approved, and a large amount of safety data has been accumulated. All these supportits further development in the field of autoimmune disorders.

## 1.3. Introduction of the Test Drug

CM313 Injection (hereinafter referred to as "CM313") is a humanized monoclonal antibody drug with independent intellectual property rights developed by KeyMed. The active ingredient of CM313 is a recombinant humanized anti-human leukocyte differentiation antigen 38 (CD38)

monoclonal antibody, belonging to the human IgG1/kappa subtype. It has a complete molecular weight of approximately 148 kDa and is composed of two identical light chains and two identical heavy chains connected by disulfide bonds. Each light chain contains 214 ammonia acids, and each heavy chain contains 449 ammonia acids. The asparagine residue (N299) at position 299 of the heavy chain is a glycosylation site, and the isoelectric point distribution is within the range of 7.9 to 8.9.

According to the Provisions for Drug Registration, this product is classified as a Category 1 therapeutic biological product.

### 1.3.1. Nonclinical Studies

Preclinical studies have confirmed that CM313 can specifically bind to CD38 on the surface of hematologic neoplasm cells such as myeloma and lymphoma. It exerts its anti-neoplasm effects by mediating ADCC, CDC, and ADCP, as well as inducing neoplasm cell apoptosis under Fc cross-linking conditions, thereby killing various CD38-positive hematological neoplasm cells. Additionally, CM313 also possesses the ability to inhibit the extracellular enzyme activity of CD38. *In vivo* and *in vitro* pharmacodynamic (PD) studies have demonstrated that CM313 exhibits comparable anti-neoplasm efficacy to daratumumab. Toxicology findings indicate that CM313 has good safety profile. Pharmaceutical findings fully demonstrate that the production process of CM313 is stable, the quality is controllable, and it has good batch-to-batch consistency.

### 1.3.2. Clinical Studies

To date, CM313 has undergone 1 clinical study, namely a Phase 1 clinical study of CM313 in patients with relapsed/refractory MM, lymphoma, and other hematological malignancies (study protocol number CM313MM001), to evaluate the safety, tolerability, preliminary efficacy, pharmacokinetics (PK)/PD, and immunogenicity of CM313 in these patients.

The study is divided into two stages: dose escalation and dose expansion. In the dose-escalation stage, 9 dose levels were initially set: 0.006 mg/kg, 0.06 mg/kg, 0.3 mg/kg, 1 mg/kg, 2 mg/kg, 4 mg/kg, 8 mg/kg, 16 mg/kg, and 24 mg/kg. The first 4 dose levels adopted accelerated dose escalation, while the subsequent dose levels employed the traditional "3+3" dose-escalation design. After completing the first intravenous infusion of CM313 at the designated dose, all enrolled subjects entered a 21-day dose-limiting toxicity (DLT) observation period. Subjects who completed the DLT observation period then entered the multiple-dose phase of CM313. The first 7 doses in the multiple-dose phase were administered once weekly (7 days), followed

by 8 doses administered every two weeks (14 days), and subsequently, treatment was given every 4 weeks (28 days) until disease progression.

As of 11 Jan 2022, a total of 10 subjects have been enrolled in the study (1 subject each in the 0.006, 0.06, 0.3, and 1 mg/kg dose levels, and 3 subjects each in the 2 mg/kg and 4 mg/kg dose levels). The enrolled subjects were aged 45 to 70 years, with a male-to-female ratio of 6:4. All subjects were MM patients who had failed at least 2 lines of prior therapy. The duration of disease for the enrolled subjects was 2 to 10 years, and the median number of prior lines of therapy received was 2 to 10.

In terms of safety, no DLTs have been observed to date. Treatment-emergent adverse events (TEAEs) occurred in 9/10 subjects, and treatment-related adverse reactions (TRAEs, i.e., IMP-related AEs) occurred in 7/10 subjects. However, the vast majority of TEAEs and all TRAEs were grade 1 or 2. The most common (incidence ≥ 2/10) TEAEs were infusion-related reactions (IRRs) (5/10), upper respiratory tract infection (3/10), hypokalemia (2/10), hyponatremia (2/10), hypertriglyceridemia (2/10), and back pain (2/10). The TRAEs experienced by the subjects were: IRR (5/10), aspartate aminotransferase (AST) increased (1/10), lymphopenia (1/10), hypertriglyceridemia (1/10), peripheral neuropathy (1/10), and rash (1/10). A total of 9 grade 3 TEAEs (no grade 4 or higher TEAEs) occurred in 4/10 subjects, all of which were unrelated to CM313 treatment.

In terms of efficacy, in the accelerated dose escalation low-dose levels (0.06, 0.3, and 1 mg/kg), all 3 subjects discontinued treatment due to disease progression. Additionally, 1 subject in the lowest dose level (0.006 mg/kg) withdrew voluntarily, with a neoplasm assessment finding of stable disease (SD). The best efficacy assessment findings for the 3 subjects in the 2 mg/kg dose level were very good partial response (VGPR), minor response (MR), and SD, respectively. For the 2 subjects in the 4 mg/kg dose level who had completed the first efficacy assessment, the neoplasm assessment findings were SD and VGPR, respectively. Currently, only 6 subjects in the 2 mg/kg and 4 mg/kg dose levels remain on CM313 study treatment.

Regarding PK, as shown in the table below, the mean PK parameters in 3 subjects in the 2 mg/kg dose level and 2 subjects in the 4 mg/kg dose level were similar to the mean PK parameters of daratumumab at the same dose. The clearance rate showed a decreasing trend with increasing dose and number of administrations, while the half-life showed an increasing trend with increasing dose and number of administrations, both exhibiting non-linear PK characteristics, suggesting target-mediated clearance of CM313.

| **Parameter** | **CM313 dose level** |
|---|---|

| | 2 mg/kg | 4 mg/kg |
|---|---|---|
| **$C_{max}$ (μg/mL)** | | |
| **1st Dose** | 42.397±2.910 | 82.225±13.272 |
| **8th Dose** | 88.543±27.318 | NA |
| **$AUC_{(0-inf)}$ (μg·h/mL)** | | |
| **1st Dose** | 1525.950±277.876 | 8688.199±4024.305 |
| **8th Dose** | 22445.339±15987.230 | NA |
| **$T_{1/2}$ (h)** | | |
| **1st Dose** | 22.5±1.0 | 88.2±40.6 |
| **8th Dose** | 168.6±76.9 | NA |
| **CL (mL/h/kg)** | | |
| **1st Dose** | 1.338±0.229 | 0.516±0.239 |
| **8th Dose** | 0.216±0.101 | NA |
| **V (mL/kg)** | | |
| **1st Dose** | 43.224±5.832 | 58.631±0.162 |
| **8th Dose** | 47.450±1.801 | NA |

In terms of PD, a decrease in the number of target cells (T/B/NK) from baseline was observed in 3 subjects in the 2 mg/kg dose level and 2 subjects in the 4 mg/kg dose level after the first dose. After multiple doses (QW*7 times), the number of T cells could recover to baseline or even above baseline levels, while NK cells remained at a lower level. The PD characteristics were similar to the clinical study findings of daratumumab.

In summary, the current data indicate that CM313 has good safety and tolerability in patients with relapsed/refractory MM. The PK and PD characteristics are similar to those of daratumumab, and preliminary efficacy has been observed in the 2 mg/kg and 4 mg/kg dose levels.

## 2. Study Objectives and Study Endpoints

| **Objectives** | **Endpoints** |
|---|---|
| Primary Objective | Primary Endpoint |
| ➢ To evaluate the safety and tolerability of multiple doses of CM313 in subjects with SLE. | ➢ Safety endpoints: AEs, laboratory tests, physical examinations, vital signs, 12-lead electrocardiogram (ECG) abnormalities, etc. |
| Secondary Objective | Secondary Endpoints |
| ➢ To evaluate the PK characteristics of multiple doses of CM313;<br>➢ To evaluate the PD characteristics of multiple doses of CM313;<br>➢ To evaluate the immunogenicity of multiple doses of CM313. | ➢ PK endpoints:<br>• PK parameters after the first dose of CM313, including time to maximum concentration ($T_{max}$), maximum concentration ($C_{max}$), area under the serum concentration-time curve over a dosing interval ($AUC_{tau}$), area under the serum concentration-time curve from time 0 to time t ($AUC_{0-t}$), etc.;<br>• PK parameters after the last dose of CM313, including $T_{max}$, $C_{max}$, $AUC_{tau}$, $AUC_{0-t}$, area under the concentration-time curve from 0 to ∞ ($AUC_{0-\infty}$), elimination half-life ($T_{1/2z}$), clearance (CL), volume of distribution ($V_z$), mean residence time ($MRT_{0-t}$), and accumulation index ($R_{ac}$), etc.<br>➢ PD endpoints:<br>• Changes and rates of change from baseline in immune cell subtypes, including T lymphocytes, B lymphocytes, pDC cells, and NK cells.<br>➢ Immunogenicity endpoints: Anti-drug antibodies (ADAs). |
| Exploratory Objectives | Exploratory Endpoints |
| ➢ To explore the preliminary efficacy and some PD characteristics of CM313 after multiple doses. | ➢ Preliminary efficacy endpoints:<br>• Change from baseline in the SELENA-SLEDAI score at each evaluation visit;<br>• Change from baseline in Physician Global Assessment (PGA) score at each evaluation visit;<br>• Time to first SLE flare after randomization;<br>• Change from baseline in prednisone dosage at each evaluation visit;<br>• Change from baseline in the BILAG-2004 score at each evaluation visit;<br>• Changes from baseline in 24-hour protein urine quantification and protein urine/creatinine ratio (only for subjects with protein urine/creatinine ratio exceeding the upper limit of normal during the screening period) at each evaluation visit;<br>• The percentage change from baseline in immunology-related endpoints [immunoglobulin G (IgG), immunoglobulin A (IgA), immunoglobulin M (IgM), immunoglobulin E (IgE), anti-double-stranded deoxyribonucleic acid (ds-DNA) antibodies, complement (C3, C4)], C-reactive protein (CRP), and erythrocyte sedimentation rate (ESR) at each evaluation visit.<br>➢ The expression levels of type I interferon (IFN)-related genes and their changes and rates of change from baseline at each assessment visit. |

# 3. Study Plan

## 3.1. Overall Study Design and Plan

This is a multicenter, randomized, double-blind, multiple-dose, dose-escalation, placebo-controlled Phase 1b/2a trial designed to evaluate the safety, tolerability, PK, PD, immunogenicity, and preliminary efficacy of CM313 in subjects with SLE.

The study plans to enroll 40 SLE subjects, with a total of 4 dose levels: 2 mg/kg, 4 mg/kg, 8 mg/kg, and 16 mg/kg, with dose escalation starting from the lowest dose. Ten subjects are enrolled at each dose level and randomized in a 4: 1 ratio to receive the respective dose of CM313 (8 subjects) or placebo (2 subjects). CM313 or placebo will be administered intravenously, observed for 28 days after the first dose and then QW for 4 consecutive doses. The 16 mg/kg dose level is optional. After all subjects in the 8 mg/kg dose level have completed the single-dose safety observation period, the sponsor and the investigator will decide whether to proceed with the 16 mg/kg dose level based on the safety data.

The multiple dose escalation scheme for this study is as follows:

| **Dose Level** | **2 mg/kg** | **4 mg/kg** | **8 mg/kg** | **16 mg/kg** |
|---|---|---|---|---|
| Total Subjects (CM313: placebo) | 10 (4:1) | 10 (4:1) | 10 (4:1) | 10 (4:1) |
| Escalation Magnitude | Starting dose | 100% | 100% | 100% |

The study design schema will be as follows (black arrows in the figure indicate drug administration):

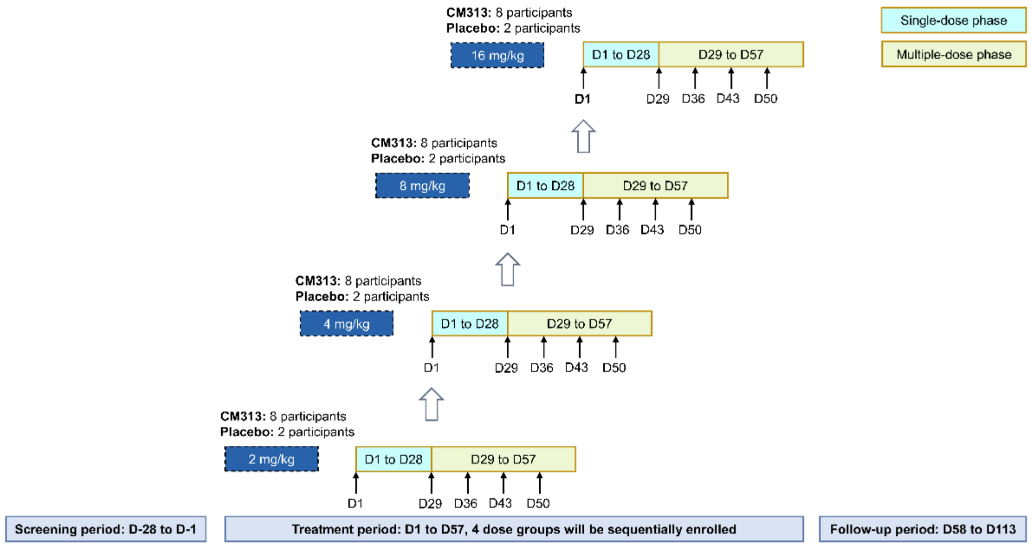


This study will consist of three periods: a screening period of up to 4 weeks, an 8-week treatment period, and an 8-week follow-up period.

**Screening Period (4 weeks)**

All participants who have signed the informed consent form (ICF) will be screened for eligibility within 28 days prior to the first dose.

If a subject is screened as ineligible due to an incidental, temporary condition (e.g., insufficient washout from prior therapy, etc.), re-screening may be performed once, but the ICF needs to be re-signed and a new screening number should be assigned.

**Treatment Period (8 weeks)**

The treatment period includes a single-dose phase (Day 1 to Day 28) and a multiple-dose phase (Day 29 to Day 57). Successfully enrolled participants will be randomized prior to the first dose administration and will receive the corresponding dose of CM313 or placebo via intravenous infusion according to their assigned dose level. Subjects will be observed for 28 days (single-dose phase) after the first dose (D1). After the safety and tolerability are assessed as acceptable by the investigator, subjects will enter the multiple-dose phase and receive four consecutive doses (QW) of the investigational medicinal product (IMP). The administration of the next dose level can be initiated after completion of the single-dose phase in the previous dose level and that there are no AEs meeting the dose escalation interruption and stopping criteria as evaluated by the investigator and the sponsor. The details are as follows:

- **Dose Levels:** Four dose levels are planned: 2 mg/kg, 4 mg/kg, 8 mg/kg, and 16 mg/kg.
- **Number of Subjects:** Each dose level will include 10 subjects (8 subjects will receive CM313, and 2 subjects will receive placebo).
- **Route and Frequency of Administration:**
  Both CM313 and placebo will be administered intravenously.
  - **Single-dose phase (D1 to D28):** On D1, subjects will receive the corresponding dose of CM313 or placebo according to their assigned dose level via intravenous infusion. Following administration, they will enter a 28-day safety observation period.
  - **Multiple-dose phase (D29-D57):** On D29, subjects will enter the multiple-dose phase and receive the corresponding dose of CM313 or placebo via intravenous infusion, once weekly (QW), for a total of 4 doses. After completing all doses, subjects will have an end of treatment (EOT) visit 1 week after the last dose.
- **Dose Escalation:** The next dose level can be initiated after all subjects at the previous dose level have completed D1-D28 safety observation without any AEs

meeting the interruption and stopping criteria for dose escalation as judged by the investigator and the sponsor.

**Follow-up Period (8 weeks)**

After completing the EOT visit, subjects will enter an 8-week follow-up period until the end of study (EOS) visit is completed.

**Dose Escalation**

The investigators and the sponsor will determine whether to proceed to the next dose level based on the safety findings of the single-dose phase for each dose level, following the principle of dose escalation.

Upon completion of the pre-defined multiple-dose administration and based on the obtained CM313 PK parameters, PD, safety, and efficacy data, the investigator and sponsor will jointly discuss whether to add new dose levels (including adjustments to dosing frequency and dose levels) and adjust sampling time points.

## 3.2. Rationale for Study Design

This study is a randomized, double-blind, placebo-controlled, dose-escalating, multiple-dose Phase 1b/2a clinical study to evaluate the safety, tolerability, PK, PD, immunogenicity, and preliminary efficacy of CM313 Injection in subjects with SLE. The primary objective is to evaluate the safety and tolerability of multiple doses of CM313 in SLE subjects, while also assessing its PK, PD, and immunogenicity characteristics, and exploring its efficacy.

In the study CM313MM001 conducted in patients with MM, multiple doses were administered once weekly after a single dose and completion of the safety observation period. As of 11 Jan 2022, the dose had been escalated to the 4 mg/kg dose level, and the findings showed that CM313 had good safety and tolerability. Therefore, in this study, subjects will receive their first dose and be observed for 28 days to fully assess the safety and tolerability of a single dose before entering the multiple-dose phase. This design is supported by CM313 clinical data, fully protects the safety and rights of subjects, and is reasonable.

This study administers the test drug/placebo on top of standard SLE treatment, ensuring the rights of subjects receiving the placebo. At the same time, the use of a placebo control and randomized grouping also meets scientific requirements and ensures the rigor of the design.

## 3.3. Raionale for Dose Selection

This study includes a total of 4 dose levels: 2 mg/kg, 4 mg/kg, 8 mg/kg, and 16 mg/kg, with dose escalation starting from the lowest dose, considering the following:

About the starting dose level:

- In the study CM313MM001, the dose level has been escalated from the 2 mg/kg dose level to the 4 mg/kg dose level. The 2 mg/kg dose level has entered the multiple-dose extension phase, with good safety and tolerability observed in subjects, and VGPR was achieved after the second dose, demonstrating preliminary efficacy. No efficacy was observed in subjects in dose levels below 2 mg/kg. To avoid ineffective exposure in SLE subjects, the sponsor plans to start dosing at the 2 mg/kg dose level in the Phase 1b/2a study of CM313 for the treatment of SLE.
- [redacted]
- Drugs targeting CD38 have already been approved for marketing, and the risk of uncontrollable target-mediated toxic effects is low. CM313 has been extensively compared with the similar drug daratumumab. [redacted]

About the setting of the highest dose level:

- The clinical dose of daratumumab, a drug with the same target, is 16 mg/kg. In a study where 2 patients with refractory and life-threatening SLE were treated with daratumumab at this dose level (16 mg/kg QW) [17], both patients showed significant clinical responses after 1 month of treatment, with good safety and tolerability.
- The "Technical Guidelines for Clinical Pharmacology Studies of Innovative Drugs (Draft

for Comments)" released in August 2021 states: In order to understand the exposure-effect relationship earlier and better, it is recommended to investigate PK and PD (if possible) within a wider dose range in single ascending dose and multiple ascending dose studies to provide a basis for the selection of regimen in subsequent clinical studies. [redacted]

- [redacted]

About the dosing frequency and treatment cycle:

- In the study CM313MM001, the dosing frequency was QW. In the study where 2 patients with refractory and life-threatening SLE were treated with daratumumab [17], both patients showed significant clinical responses after 1 month of treatment. The treatment regimen was daratumumab 16 mg/kg, intravenous infusion, QW, for 4 consecutive doses. This product draws on the experience of the study CM313MM001 and daratumumab, and sets QW continuous administration for 4 times to initially explore the safety and efficacy in SLE subjects.

Currently, the study CM313MM001 is ongoing and has progressed to the multiple-dose escalation stage, with dose levels at 2 mg/kg QW and above. At the start of this study, it is expected that the study CM313MM001 can provide more safety and efficacy data of CM313 at higher dose levels to support the conduct of this study.

In summary, based on the preclinical data of this product, the clinical data of CM313MM001, and the experience of daratumumab, a drug with the same target, the applicant plans to conduct a Phase 1b/2a clinical study of CM313 for the treatment of SLE with the dosing regimen of 2 mg/kg, 4 mg/kg, 8 mg/kg, and 16 mg/kg, escalating from a low dose, intravenous infusion, with observation for 28 days after the first dose, then followed by 4 consecutive doses.

### 3.4. Benefit/Risk Assessment

Many immune cells involved in the pathogenesis of SLE express CD38 or express CD38 upon stimulation. Studies have shown that compared to healthy individuals, SLE patients have

increased CD38 expression in different leukocyte sub-populations, such as CD4+, CD8+ memory T cells, and monocytes. Due to the high expression of CD38 on the surface of plasmablasts and plasma cells that produce autoantibodies, it is considered a potential target for the treatment of autoimmune diseases driven by autoantibodies.

Monoclonal antibodies targeting CD38 can effectively kill autoantibody-producing plasmablasts and plasma cells. The mechanisms by which CD38 antibodies kill target cells primarily involve classical immune effects like ADCC, CDC, and ADCP, and may also continuously promote the death of autoantibody-producing plasmablasts and plasma cells through antibody-mediated apoptosis and regulation of ADPR enzyme activity.

Daratumumab (trade name: Darzalex), a drug with the same target, is a fully human IgG1 antibody drug. It received accelerated approval from the FDA in November 2015 and was subsequently approved by the EMA and PMDA in April and July 2017, respectively. It will be used to treat adult patients with relapsed or refractory MM who have previously received treatment (including a protease inhibitor and an immunomodulatory agent) and whose condition has aggravated after treatment. In a study published in the *New England Journal of Medicine* in September 2020, researchers used daratumumab to treat 2 patients with refractory and life-threatening SLE. The treatment regimen was daratumumab 16 mg/kg administered once weekly (QW). After 1 month of treatment, both patients showed significant clinical responses, along with the elimination of long-lived plasma cells, decreased type I interferon activity, and downregulation of T cell activation and antigen stimulation-related gene transcription. These findings provide preliminary evidence for the feasibility of using anti-CD38 mAbs to treat SLE.

In conclusion, preliminary efficacy of CD38 mAbs has been observed in patients with refractory SLE. The CM313 mAb is expected to improve or alleviate the clinical symptoms of active SLE and may prolong the lifespan of SLE patients.

To date, there are no confirmed important risks for CM313. Based on the safety information of similar products already on the market, IRR, neutropenia/thrombocytopenia, interference with indirect antiglobulin test (IAT), and hepatitis B virus reactivation are listed as potential important risks of CM313.

As of 11 Jan 2022, the clinical trial data of CM313MM001 are as follows: 1) Safety: No DLT events have occurred so far. TEAEs occurred in 9/10 subjects and TRAEs occurred in 7/10 subjects, but the vast majority of TEAEs and all TRAEs were Grade 1 or 2. All Grade 3 TEAEs were judged to be unrelated to the IMP; 2) Efficacy: The best efficacy evaluation results of the

3 subjects in the 2 mg/kg dose level were VGPR, MR, and SD, respectively; while the neoplasm evaluation results of the 2 subjects in the 4 mg/kg dose level who had completed the first efficacy evaluation were SD and VGPR, respectively. The above findings indicate that CM313 has good safety and tolerability in patients with relapsed/refractory MM and lymphoma, and has shown preliminary efficacy.

Based on the mechanism of action of CM313, pharmacology findings, and preliminary efficacy findings, CM313 is expected to have potential benefits for the treatment of active SLE. Based on the available safety findings of CM313 and the safety data of the marketed product with the same target, daratumumab injection, CM313 has good safety and tolerability. Therefore, it can be considered that the overall benefits of CM313 outweigh the risks.

### 3.5. Number of Subjects

The study plans to enroll 40 SLE subjects, with a total of 4 dose levels: 2 mg/kg, 4 mg/kg, 8 mg/kg, and 16 mg/kg, with dose escalation starting from the lowest dose. Ten subjects are enrolled at each dose level and randomized in a 4: 1 ratio to receive the respective dose of CM313 (8 subjects) or placebo (2 subjects).

### 3.6. Study Period

This study includes a 4-week screening period, an 8-week treatment period, and an 8-week follow-up period.

### 3.7. Selection of Study Population

#### 3.7.1. Inclusion Criteria

A subject will be eligible for inclusion in this study only if all of the following criteria apply:

1. Males or females, aged ≥ 18 and ≤ 65 years old;
2. Diagnosed with SLE according to the 2019 European League Against Rheumatism (EULAR)/American College of Rheumatology (ACR) classification criteria for SLE (see Appendix 1) based on documented medical history and/or examination results.
3. SELENA-SLEDAI score > 0 and ≤ 12 during the screening period;
4. Positive serological findings for autoantibodies during the screening period, defined as: positive antinuclear antibody (ANA) and/or positive anti-ds-DNA antibody as determined based on the reference range of each study site's laboratory; borderline findings will not be accepted;
5. Maintain a stable standard of care regimen for at least 30 days before the first dose of

IMP. Stable standard of care regimen refers to the stable use of any of the following (alone or in combination): corticosteroids, antimalarials, nonsteroidal anti-inflammatory drugs (NSAIDs), immunosuppressants or immunomodulators, such as azathioprine, mycophenolate (including mycophenolate mofetil, mycophenolate sodium), methotrexate, leflunomide, tacrolimus, ciclosporin [Note: Routine dose reduction of corticosteroids ≤ 20 mg/d prednisone (or equivalent dose of other corticosteroids) will also be considered as stable therapy];

6. Subjects and their partners agree to use effective contraception during the following time periods (from screening to 4 months after the last dose of IMP for female subjects and their partners, from screening to 6 months after the last dose of IMP for male subjects and their partners) (see Appendix 6);
7. Capable of understanding the nature of the study and voluntarily signing the ICF;
8. Able to communicate well with the investigator and complete all visits according to the protocol requirements.

### 3.7.2. Exclusion Criteria

A subject will not be eligible for inclusion in this study if any of the following criteria apply:

1. Renal disorder: Severe lupus nephritis (defined as urine protein > 6 g/24 hours or serum creatinine > 2.5 mg/dL or 221 µmol/L) within 8 weeks before the first dose of IMP, or active nephritis requiring treatment with drugs prohibited by the protocol, or conditions requiring hemodialysis, or treatment with prednisone ≥100 mg/d or equivalent glucocorticoids for ≥ 14 days;
2. Subjects with central nervous system diseases caused by SLE or non-SLE (including epilepsy, psychosis, organic encephalopathy syndrome, cerebrovascular accident, encephalitis, central nervous system vasculitis) within 8 weeks before the first dose of IMP;
3. The presence of the following laboratory test abnormalities, including but not limited to:
   a) Subjects with abnormal hepatic function, such as aspartate aminotransferase (AST) or alanine aminotransferase (ALT) > 2 times the upper limit of normal (ULN), total bilirubin > 1.5 times ULN, or

b) Subjects with abnormal renal function: creatinine (Cr) or urea nitrogen (BUN) > 1.5 times ULN; screening glomerular filtration rate (eGFR) ≤ 60 mL/min/1.73 $m^2$, or
c) Subjects with abnormal hematology results: white blood cell count < $2.5\times10^9$/L, hemoglobin < 85 g/L, platelet count < $50x10^9$/L;

4. Subjects with a history of clinically significant diseases that the investigator believes will pose a risk to the subject's safety, or that will affect the safety or efficacy analysis and evaluation if the disease/condition worsens during the study (such as abnormalities of the circulatory system and endocrine system, nervous system diseases, blood system diseases, immune system disorders, mental illness, and unstable metabolic abnormalities), for example: 1) cardiovascular disorder: history of acute myocardial infarction, or unstable angina pectoris, severe arrhythmia (multifocal premature ventricular contractions, ventricular tachycardia, ventricular fibrillation) within 6 months before screening; New York Heart Association (NYHA) Class III-IV; 2) subjects with known moderate or severe persistent asthma within 5 years before screening, or subjects with ongoing inadequately controlled asthma;
5. Subjects who may have active mycobacterial infection, defined as: chest X-ray (posterioranterior and lateral position) examination suggests active tuberculosis infection within 3 months prior to screening/during the screening period (if required by the ethics, tuberculosis testing will be performed according to site procedures);
6. Subjects with active hepatitis, positive hepatitis B surface antigen (HBsAg), positive hepatitis B core antibody (HBcAb) + positive hepatitis B virus (HBV) deoxyribonucleic acid (DNA), or positive hepatitis C virus (HCV) antibody at screening;
7. A history of human immunodeficiency virus (HIV) infection, or HIV antibody positive at screening;
8. Subjects with treponema pallidum antibody positive at screening;
9. Having chronic active infection or acute infection requiring systemic treatment with antibiotics, antivirals, antiparasites, antiprotozoals or antifungals within 4 weeks prior to screening, or having superficial skin infections requiring treatment within 1 week prior to screening. Note: After the infection is cured, the patient may be re-screened once;
10. Subjects with known or suspected history of immunosuppression, including history of invasive opportunistic infections (e.g., histoplasmosis, listeriosis, coccidioidomycosis, pneumocystosis, and aspergillosis), even if the infection has recovered; or have unusual frequent, recurrent, or prolonged infections (as judged by the investigator);

11. Major surgery (craniotomy, thoracotomy, or laparotomy) or unhealed wounds, ulcers, or fractures within 4 weeks prior to the first dose of IMP, or major surgery planned during the study;
12. Subjects with malignancy within 5 years prior to screening (except for completely cured cervical carcinoma in situ and non-metastatic squamous cell or basal cell carcinoma of the skin);
13. A history of major organ transplant (e.g., heart, lung, kidney, liver) or hematopoietic stem cell/or bone marrow transplant;
14. Subjects who received live or live attenuated vaccines within 4 weeks prior to the first dose of IMP or plan to receive the above during the study;
15. Subjects who have participated in any clinical trial within 4 weeks before the first dose of IMP or who are within 5 half-lives of the investigational product in the previous clinical trial (whichever is longer);
16. Targeted drug therapy: received rituximab within 180 days before the first dose of IMP; received any drug therapy targeting T or B lymphocytes, cytokines, or receptors (e.g., belimumab, telitacicept, abatacept, etc.) within 180 days before the first dose of IMP; received JAK inhibitor therapy within 30 days before the first dose of IMP; or within 5 half-lives after discontinuation of the above drugs (whichever is longer);
17. Received intravenous cyclophosphamide within 180 days before the first dose of IMP or oral cyclophosphamide within 30 days before the first dose of IMP;
18. Used intravenous immunoglobulins (IVIG), prednisone ≥ 100 mg/d or equivalent glucocorticoids, or undergone plasmapheresis within 28 days before the first dose of IMP;
19. Used IL-2, thalidomide, Tripterygium wilfordii Hook F, or drug products containing Tripterygium wilfordii Hook F within 28 days before the first dose of IMP;
20. Known allergy to monoclonal antibodies or to excipients of CM313;
21. Patients with depression or tendance for suicide;
22. A history of large alcohol consumption [i.e., more than 14 units of alcohol per week (1 unit = 360 mL of beer or 45 mL of liquor containing 40% alcohol or 150 mL of wine] or a history of drug abuse within 3 months prior to screening;
23. Female subjects who are pregnant or lactating, or planning to become pregnant or breastfeeding during the study; males whose partners plan to become pregnant during the study;

24. Any reason that, in the opinion of the investigator, contraindicates the subject's participation.

### 3.7.3. Subject Re-screening

Subjects who do not meet the study entry requirements due to laboratory tests (e.g., serum chemistry, hematology, etc.) will be allowed one retest of the laboratory test. The retest time should still meet the overall time requirements for the screening period in the study. Re-screening will not be allowed for subjects who ultimately fail screening due to laboratory tests and thus do not meet study entry requirements.

If a subject fails screening due to an incidental AE or the washout period of a concomitant drug, etc., and does not meet the inclusion criteria, the subject may be re-screened, but a new ICF must be signed and a new screening number assigned.

### 3.7.4. Criteria for Withdrawal of Subject

For all subjects who have signed the ICF and are eligible for screening to be enrolled in the study, as long as the full course of the clinical trial is not completed whenever and for any reason, that will be considered as early withdrawal cases. Reasons for withdrawal include, but are not limited to, the following:

1) The investigator considers it necessary for the subject to withdraw from the study from the perspective of medical ethics.
2) The subject has an intolerable AE and is assessed by the investigator to be ineligible to continue the study for safety reasons.
3) The subject needs to receive prohibited treatment during the study due to AEs or other reasons and is not suitable to continue the study as assessed by the investigator.
4) The subject has poor compliance and may affect the assessment of study results as assessed by the investigator.
5) The subject is lost to follow-up.
6) The subject withdraws informed consent.
7) Other circumstances that, in the opinion of the investigator, are necessary for the subject to withdraw from the study.

Participation in this study is entirely voluntary. Subjects may withdraw from the study at any time. The subjects should inform the investigator and withdraw from the study in a timely manner if they are no longer eligible to continue study treatment for any reason. The investigator should inquire about the reason for withdrawal and record it in the source

document and electronic case report form (eCRF), and make every effort to ask the subject to return to the study site to complete the early withdrawal visit.

If possible, the subject's safety and efficacy results should be collected and recorded as thoroughly as possible. If a subject withdraws from the study and withdraws the informed consent, no further data may be collected, but the sponsor may retain and continue to use data already collected prior to withdrawal of consent. For subjects withdrawn from the study, the investigator and the sponsor will discuss and decide whether additional subjects are required for the study based on the collected data and their availability.

## 3.8. Study Treatment

### 3.8.1. Investigational Medicinal Products

The IMPs used in this study include CM313 and its placebo, both manufactured by Chengdu Kangnuoxing Biopharma, Inc.

**Test Drug: CM313 Injection**

Strength: 100 mg (5 mL)/vial. Storage conditions: 2-8°C, protected from light. Expiration date: tentatively 2 years after production.

**Control Drug: placebo**

Strength: 5 mL/vial. Storage conditions: 2-8°C, protected from light. Expiration date: tentatively 2 years after production.

### 3.8.2. Mode of Administration

#### 3.8.2.1 Administration of the Investigational Medicinal Products

This study has 4 dose levels: 2 mg/kg, 4 mg/kg, 8 mg/kg, and 16 mg/kg. Subjects will receive the corresponding dose of CM313 or an equal volume of placebo according to the corresponding dosing frequency of their respective dose levels. After the first dose, they will be observed for 28 days, and then enter the multiple-dose phase, QW, with 4 continuous doses. Both CM313 and placebo are administered by intravenous infusion. The 16 mg/kg dose level is an optional. After all subjects in the 8 mg/kg dose level have completed the single-dose safety observation period, the sponsor and investigator will decide whether to proceed with the 16 mg/kg dose level based on the safety data.

#### 3.8.2.2 Preparation and Infusion of Investigational Medicinal Product Infusion Solution

The subject's weight should be measured before each administration (can be rounded to the nearest whole number for calculation), to calculate the required drug dose. The calculation equation is as follows:

IMP dose = subject's weight (kg) × assigned dose level (mg/kg)

The appropriate strength and type of diluent will be selected for preparation based on the dose level received by the subject. See the CM313 Injection Pharmacy Manual for details.

Undiluted/unprepared IMP cannot be used directly for infusion. The prepared IMP infusion solution can be administered by intravenous infusion, and intravenous bolus or rapid push injection is prohibited.

The infusion of the IMP infusion solution will be conducted in a ward equipped with full resuscitation facilities and under the direct supervision of an experienced rheumatologist. The infusion rate will follow the recommendations outlined in Table 3, and escalation of the infusion rate will only be considered in the absence of IRRs. It is recommended that subjects limit their off-bed activities from the start of the infusion until 2 hours after the infusion is completed.

**Table 3 Infusion Rate of the Investigational Medicinal Product Infusion Solution**

| Dose Level (mg/kg) | Infusion Rate[a] | | | | |
|---|---|---|---|---|---|
| | 0-60 minutes (mL/h) | 60-90 minutes (mL/h) | 90-120 minutes (mL/h) | 120-180 minutes (mL/h) | 180 minutes to the completion of infusion (mL/h) |
| 2 | 50 | 75 | 100 | 150 | 200 |
| 4 | 50 | 75 | 100 | 150 | 200 |
| 8 | 50 | 75 | 100 | 150 | 200 |
| 16 | 50 | 75 | 100 | 150 | 200 |

**a** An increase of infusion rate will only be considered in the absence of IRRs.

Subjects will be closely monitored during the infusion, and if an IRR of any grade occurs, the infusion should be interrupted immediately and symptomatic treatment should be considered.

#### 3.8.2.3 Pre- and Post-infusion Medications of Investigational Medicinal Product Infusion Solution

To reduce the risk of IRRs, the following medications are recommended for each subject, before and after each IMP infusion:

Pre-infusion medications (within 1-3 hours before infusion):

- Corticosteroids (intermediate-acting or long-acting): It is recommended to administer 100 mg methylprednisolone (or equivalent drug) intravenously before the 1st and 2nd IMP administrations; starting from the 3rd IMP administration, the corticosteroid dose can be reduced to 60 mg methylprednisolone (or equivalent drug), orally or intravenously. If patients receive the aforementioned methylprednisolone (or equivalent drug) as pre-infusion medication on the IMP administration day, they should not receive additional corticosteroids as part of their standard SLE treatment.
- Paracetamol, 650 mg to 1000 mg, oral.
- Antihistamines: Diphenhydramine, 25 mg to 50 mg (or equivalent), oral or intramuscular.

Post-infusion medications:

- Starting from the day after the completion of the infusion, subjects should receive corticosteroids for 2 consecutive days (20 mg methylprednisolone or equivalent drug daily). If the oral corticosteroid dose in the subject's standard SLE treatment is ≥ the above dose, no additional corticosteroids will be given; if the oral corticosteroid dose in the subject's standard SLE treatment is < the above dose, corticosteroids will be supplemented to the above dose).
- In addition, for subjects with a history of chronic obstructive pulmonary disease (COPD), post-infusion medications may be considered, including short-acting and long-acting bronchodilators and inhaled corticosteroids.

#### 3.8.2.4 Adjustment for Infusion-related Reactions

If a subject experiences IRR of different levels, the investigator will provide corresponding symptomatic treatment based on the subject's condition, and it is recommended to adjust the IMP according to the following description:

- For mild to moderate IRRs: The IMP infusion should be paused. After the symptoms of IRR subside, infusion may be resumed, but the rate should not exceed half of the infusion rate at the time of the IRR. After resuming the infusion, if the subject does not experience any further IRR, the infusion rate may be increased based on the subject's condition, up to the maximum protocol-allowed infusion rate of 200 mL/hour.
- For severe IRRs: When a severe IRR first occurs, the IMP infusion should be paused. After the symptoms of IRR subside, infusion may be resumed, but the rate should not exceed half of the infusion rate at the time of the IRR. After resuming the infusion, if the subject does not experience any further IRRs, the infusion rate may be increased

based on the subject's condition, up to the maximum infusion rate of 200 mL/hour allowed by the protocol. Following the resumption of the infusion, if the subject experiences a second severe IRR, the above steps will be repeated (the minimum allowable infusion rate for the IMP is 12.5 mL/hour). If the subject experiences a third severe IRR, the IMP should be permanently discontinued.

- Life-threatening IRRs: The IMP infusion should be immediately stopped and the IMP should be permanently discontinued. Administer bedside rescue.

#### 3.8.2.5 Dose Modifications

Dose modifications of IMP received by individual subjects are not allowed during the treatment period. However, if dosing is interrupted due to an AE or other reasons, the investigator will consider resuming dosing, discontinuing treatment, or withdrawing the subject from the study depending on the specific circumstances.

### 3.8.3. Randomization and Blinding

#### 3.8.3.1. Randomization

A randomized design is used to complete the randomization of subjects and IMP using an interactive web response system (IWRS). A subject randomization list and an IMP randomization list will be generated by the randomization statistician in each dose level using statistical analysis software (SAS). Subjects who meet the inclusion criteria will be randomized to theCM313 group and placebo group in the proportion of each dose level, and will be imported into the IWRS by the system engineer. After successful screening of subjects, study personnel at each site will log into the IWRS to obtain the subject randomization number. When dispensing drugs at each visit, the investigator and the designated personnel will log into the IWRS to obtain the drug number and assign the IMPs according to the drug number. The randomization statistician will record the parameters such as the seeds of generating random numbers in the blind code. The emergency unblinding function will be turned on after importing the blind code into the IWRS.

Subjects will be assigned a unique screening number. After a subject signs the ICF, the subject will be assigned a separate enrollment number in the order of eligibility for screening, and the enrollment number is the randomization number.

#### 3.8.3.2. Blinding

A double-blind design will be used in the study. CM313 and placebo will be provided by the sponsor in vials, which are indistinguishable to the naked eye. Drug blinding will be performed by the randomization statistician and the blinding personnel who do not participate in the clinical trial, i.e., paste the printed drug label at the designated position of each drug according to the blind code. The randomization statistician will supervise the drug blinding and instruct the blinding operator to label according to the blind code. After completion of blinding, the blind code will be sealed by the randomization statistician. The entire blinding process must be documented in writing. The blinding personnel shall not participate in other relevant work of this clinical trial, and shall not disclose the blind code to any personnel participating in the clinical trial.

#### 3.8.3.3. Unblinding

In this study, after all data are entered into the database, unblinding will be performed after the blind data review, database lock, and finalization of the Statistical Analysis Plan (SAP), , and then the data will be submitted to the statistical analysis personnel for statistical analysis. The unblinding process will be performed by the principal investigator, statistician, IWRS party, sponsor or its representatives, and will be recorded accordingly.

In case of emergency unblinding by the investigator, the specific grouping information of the subject will be obtained through the emergency unblinding module of the IWRS. If emergency unblinding is required, the principal investigator and relevant sponsor personnel will be notified as far in advance as possible before unblinding the IMP. The specific treatment group information of the subjects can only be obtained after approval by the principal investigator. In case of failure to contact the sponsor before emergency unblinding, the sponsor must be contacted within 24 hours of unblinding. Once emergency unblinding is performed, the corresponding subjects are dropped out. The date and reason for unblinding the subject, and the procedure of unblinding should be documented by the investigator in the source document. If an AE occurs at population level or the study is interrupted for any reason, early unblinding may be performed with the mutual approval of the sponsor and the investigator.

### 3.8.4. Packaging and Labeling of Investigational Medicinal Products

IMPs will be packaged in accordance with regulatory requirements. Each small package box and vial will be labeled in accordance with applicable regulatory requirements. Drug labels will follow a unified format, and the label content will include, but not be limited to: clinical

IMP name (indicating "For Clinical Trial Use Only"), strength, storage conditions, batch numbers, identification numbers, manufacture date, shelf-life, and sponsor, etc. During drug administration, attention should be paid to verifying IMP-related information and operating in accordance with the drug preparation process specifications. If the label is missing or damaged, the drug is prohibited from use.

A complete record of all IMP numbers and quantities, along with drug labels, will be maintained in the sponsor's study file.

### 3.8.5. Storage and Management of Investigational Medicinal Products

#### 3.8.5.1. Storage of Investigational Medicinal Products

IMPs will be provided free of charge by the sponsor and distributed to each study site as planned. All drugs will be managed by a dedicated drug administrator at the study site to ensure that the drugs can only be used for subjects eligible for inclusion in the clinical study. Distribution of IMPs to non-subjects is prohibited. The monitor will be responsible for monitoring the supply, use, and storage of the clinical IMPs and the disposal of remaining IMPs. IMPs should be stored in a locked drug cabinet as required, under the following storage and transportation conditions: 2-8°C, protected from light.

#### 3.8.5.2. Counting of Drugs

Clinical study site is responsible for the periodic accountability, quality control and recording of the IMPs. During the study, the investigator or designee must record the quantity of drugs, as well as the missing drugs, i.e., IMPs shall be kept, distributed, received, and recovered by special personnel of the study site. Detailed records are required for the process.

The investigator should ensure the accuracy of IMP count records throughout the study. Use of each dose of IMP will be recorded in the eCRF. The investigator should return all unused or partially used IMP, empty post-use vials to the sponsor after the end of the study and confirm that no remaining drug is left at the study site.

### 3.8.6. Dose Escalation Interruption and Stopping Criteria

After the completion of the safety observation period following the first dose of IMP for each group of subjects, the severity of AEs will be assessed. The causal relationship of each AE to the IMP will be assessed as described in Section 6.1.4, and AEs that are "definitely related", "probably related" and "possibly related" will be considered related to the IMP. The investigator and the sponsor will decide whether to continue dose escalation of the IMP based

on the safety and tolerability data obtained from all subjects who have completed the previous dose level.

The interruption of dose escalation may be considered if moderate or higher IMP-related AEs with the same system organ class (SOC) are observed in 50% or more of the subjects at that dose level, or if there is an unacceptable AE that the investigator believes is reasonably attributable to the pharmacological effect of the IMP, or if more than 1 subject experiences an SAE in the same SOC, etc.

**If dose escalation is interrupted, the investigator will decide on one of the following solutions after discussion with the sponsor:**

- Continue to escalate to the next dose level as specified in the protocol; or
- Add an intermediate dose or adjust dosing frequency.

**If the interruption of dose escalation occurs for a second time during the study, dose escalation should be stopped.**

Note: AEs definitely due to other reasons will not be considered AEs that meet the interruption and stopping criteria for dose escalation. If any IMP-related SAE occurs during the study, the study needs to be suspended, and the investigator will discuss and analyze the reasons with the sponsor and determine the impact on the subsequent study.

### 3.8.7. Premature Discontinuation of Treatment

Premature discontinuation of the subject from IMPs is required if any of the following occurs:

- As requested by the subject;
- The subject experiences an intolerable AE related to the IMP as assessed by the investigator;
- The subject experiences any disease that precludes continuation of study treatment, such as any opportunistic infection suggestive of possible immune impairment or other recurrent/long-term infection (as assessed by the investigator) with an abnormal frequency, malignancy (except cervical carcinoma in situ, non-metastatic skin squamous cell carcinoma, and basal cell carcinoma) or serious cardiovascular and cerebrovascular disease;
- The female subject becomes pregnant;
- The subject takes prohibited concomitant medications that leads to discontinuation of IMPs;
- The subject requires early discontinuation of study treatment due to inadequate response;

- The subject has poor compliance with study treatment or protocol requirements;
- The investigator/sponsor decides to discontinue the subject's study treatment.

If a subject discontinues treatment early but does not withdraw from the study, the EOT visit and subsequent follow-up visits should be completed as far as possible. Subjects who discontinue the study treatment prematurely may be replaced following discussion between the investigator and the sponsor.

### 3.8.8. Concomitant Therapies

All drug therapy (including nutritional supplements) and non-drug therapy received by the subject from the time of signing the ICF until the last visit need to be collected and recorded in the electronic data capture (EDC) system.

To prevent IRRs, all subjects need to receive prophylactic medications before and after each infusion.

Subjects enrolled in this study need to maintain a stable standard of care regimen for at least 30 days before the first dose of IMP. After the subject is randomized and receives the first dose of IMP, the investigator can adjust the standard of care according to the changes in the subject's condition.

#### 3.8.8.1. Standard of Care

The standard of care regimen refers to the use of any of the following (alone or in combination): corticosteroids, antimalarials, NSAIDs, immunosuppressants or immunomodulators [including azathioprine, mycophenolate (including mycophenolate mofetil, mycophenolic sodium), methotrexate, leflunomide, tacrolimus, ciclosporin].

Antimalarials in the standard of care regimen: hydroxychloroquine maximum dose of 400 mg/day. Quinacrine (Atabrine, Mepacrine) maximum dose of 100 mg/day.

The maximum dose of corticosteroids (equivalent to prednisone dose) in the standard of care regimen is 1 mg/kg/day, and maximal daily dose should be no more than60 mg/day. For subjects with SLE treated with corticosteroids every other day, the average daily dose of corticosteroids is calculated by taking the average of the 2-day dose.

The maximum dose of azathioprine in the standard of care regimen is 200 mg/day. The maximum dose of mycophenolate mofetil (MMF) is 2 g/day. The maximum dose of methotrexate is 25 mg/week. The maximum dose of ciclosporin is 4 mg/kg/day. The maximum dose of tacrolimus is 0.1 mg/kg/day. The maximum dose of leflunomide is 40 mg/day.

If the subject's clincal condition changes, the standard of care regimen is allowed to be adjusted as needed per the clinical condition, and the reason for adjusting the treatment regimen needs to be recorded.

### 3.8.8.2. Reference for Glucocorticoid Medication

During the trial, inhaled and topical corticosteroids are allowed to treat diseases other than SLE. The duration of systemic corticosteroid treatment for diseases other than SLE should not exceed 7 days, and the dose should be reduced after 7 days.

When using corticosteroids to treat SLE-related disease activity, please refer to the "2020 Chinese Guidances for the Diagnosis and Treatment of Systemic Lupus Erythematosus" [1]: An individualized hormone therapy regimen should be established according to the type and severity of disease activity and affected organs, and the minimal dose adequate for disesase control should be used; for patients with mild active SLE, if hydroxychloroquine or NSAIDs are ineffective, low-dose hormones ($\leq$ 10 mg·$d^{-1}$ prednisone or other hormones of equivalent dose) may be considered; for patients with moderate active SLE, hormones (0.5-1 mg·$kg^{-1}$·$d^{-1}$ prednisone or other hormones of equivalent dose) combined with immunosuppressants can be used for treatment; for patients with severe active SLE, hormones ($\geq$ 1 mg·$kg^{-1}$·$d^{-1}$ prednisone or other hormones of equivalent dose) combined with immunosuppressants can be used for treatment, and after the condition is stable, the dose of hormones should be adjusted appropriately; for SLE patients with lupus crisis, hormone pulse combined with immunosuppressants can be used for treatment (hormone pulse therapy is intravenous infusion of methylprednisolone 500-1000 mg/d, usually for 3 consecutive days as a course of treatment, with an interval of 5-30 d between courses. After pulse therapy, oral prednisone 0.5-1 mg·$kg^{-1}$·$d^{-1}$ or other hormones of equivalent dose should be administered, usually for 4-8 weeks, but the specific course of treatment should depend on the condition); clinicians should pay close attention to the disease activity of SLE patients, and adjust the dose of hormones according to the degree of disease activity, and for patients with long-term stable condition, gradual discontinuation of hormones may be considered.

The specific calculation method for the average daily dose of glucocorticoids within 7 days before the visit is the average dose of all glucocorticoids administered intravenously, intramuscularly, orally, and intradermally for the treatment of SLE and non-SLE within 7 days before the visit. If acute exacerbation occurs during the study, the amount of glucocorticoids used in the treatment of acute exacerbation will be included in the calculation of total amount of glucocorticoids.

According to the "Expert Consensus on the Rational Use of Glucocorticoids in Patients with Systemic Lupus Erythematosus" (China, 2014) [18], during the maintenance treatment period, the dose of glucocorticoids should be slowly reduced at a rate of 10% of the original dose every 1-2 weeks; after the dose is reduced to 0.5 $mg \cdot kg^{-1} \cdot d^{-1}$ of prednisone (0.4 $mg \cdot kg^{-1} \cdot d^{-1}$ of methylprednisolone), the rate of dose reduction should be appropriately slowed down according to the disease condition.

### 3.8.8.3. Prohibited Concomitant Medications or Therapies

The use of concomitant medications and non-drug therapies that interfere with the evaluation of the efficacy of IMPs or affect the condition of SLE is prohibited during the trial, mainly including but not limited to the following:

- Other investigational products (biological or non-biological agents);
- Other drugs for the treatment of SLE in addition to the standard of care regimen, such as rituximab or other biological agents for the treatment of SLE, tumour necrosis factor inhibitors, interleukin receptor blockers, etc.;
- Intravenous immunoglobulins;
- Plasma exchange, leukocyte separation;
- Live vaccines or attenuated live vaccines;
- Cyclophosphamide;
- Thalidomide, iguratimod, Chinese herbal medicines and drug products containing Tripterygium wilfordii.

### 3.8.8.4. Permitted Concomitant Medications or Therapies

If other diseases occur during the study, drugs that do not affect the efficacy of the IMP (such as antibiotics) can be used, but the reasons for medication and medication conditions should be recorded in detail.

If vaccination with the COVID-19 vaccine (limited to inactivated vaccines) is planned during the study, the vaccination time should be ≥ 7 days apart from the administration of the IMP.

All medications/therapies other than the IMP, including Chinese herbal medicines and some other non-traditional therapies, are considered concomitant therapies. All concomitant therapies should be recorded in the eCRF, including but not limited to the following information: generic name of the drug, dose level, route of administration, start date, end date, and indication, etc. Any dose adjustments or changes in the treatment regimen of any concomitant therapies should be recorded in the eCRF.

#### 3.8.9. Medication Compliance

In the recruitment and screening periods, details about the purpose of this study, the basic information of the IMPs, protocols, study procedures, dosing regimens (e.g., dose, route of administration, etc.), clinical observation, frequency and procedures of and the biological sample collection, potential risks of participating in the study, compensation and reparation, etc. should be introduced to the subjects so as to make the subjects fully informed and participate voluntarily, to improve drug administration compliance. Prior to dosing, the subject number and dose administered should be carefully checked.

The site should record and maintain shipment and receipt records of the IMPs, and the sponsor should be informed immediately of any problems with quality, quantity, or cold chain transportation. The site should accurately record dose preparation information to ensure that all relevant/required information regarding dose preparation and administration is documented in the dose source document and that appropriate dose information is entered into the eCRF. Such information should be provided to the sponsor's authorized monitoring representative during the monitoring visit. After termination of the study, or at the request of the sponsor, the drug administrator must return the remaining IMPs and empty vials to the sponsor.

### 3.9. Endpoints

#### 3.9.1. Safety Endpoints

Safety evaluation endpoints include: AEs (including TEAEs, SAEs, TEAEs leading to early withdrawal, etc.), abnormalities in physical examination, vital signs, 12-lead ECG, laboratory tests (including hematology, blood chemistry, urinalysis, and coagulation function), etc.

| | |
|---|---|
| Physical Examination | Head and face, eyes, ears, nose and throat, oral cavity, skin, lymph nodes, respiratory system, cardiovascular system, abdomen, genitourinary system (if necessary), musculoskeletal system, nervous system, and mental status. |
| Vital Signs | Body temperature, pulse, respiration, and blood pressure |
| 12-lead ECG | Heart rate, PR interval, QRS duration, QT interval, and QTcF |
| Hematology | Red blood cells, hemoglobin, white blood cells, hematocrit, platelets, neutrophil count and percentage, lymphocyte count and percentage, monocyte count and percentage |
| Blood Chemistry | Glucose, total cholesterol, low density lipoprotein cholesterol, high density lipoprotein cholesterol, alanine aminotransferase, aspartate aminotransferase, total protein, albumin, total bilirubin, direct bilirubin, lactate dehydrogenase, alkaline phosphatase, glutamyl transpeptidase, creatinine, urea/urea nitrogen, uric acid, sodium, potassium, chloride, calcium |
| Urinalysis | Urine protein, urine glucose, ketones, occult blood, microscopic examination (including red blood cells/HPF, white blood cells/HPF, poikilocytes, casts and |

| | |
|---|---|
| | specific classification), urobilinogen, urine bilirubin, nitrite, urine specific gravity, acidity or alkalinity. |
| Coagulation | Prothrombin time, activated partial thromboplastin time, thrombin time, fibrinogen, international normalized ratio |

### 3.9.2. Pharmacokinetic Endpoints

PK evaluation endpoints include CM313 blood concentration, PK parameters after the first dose [including $T_{max}$, $C_{max}$, $AUC_{tau}$, $AUC_{0-t}$, etc.], PK parameters after the last dose [including $T_{max}$, $C_{max}$, $AUC_{tau}$, $AUC_{0-t}$, $AUC_{0-\infty}$, $T_{1/2z}$, CL, $V_z$, $MRT_{0-t}$, $R_{ac}$, etc.].

### 3.9.3. Pharmacodynamic Endpoints

PD evaluation endpoints include: immunophenotyping, including T lymphocytes (CD3/CD4/CD8/CD38), B lymphocytes (CD19/CD138/CD27/CD38), pDC cells (CD123/BDCA2/CD38), and NK cells (CD56/CD16/CD38); type I IFN-related gene expression.

### 3.9.4. Immunogenicity Endpoints

Immunogenicity evaluation endpoints include ADA.

### 3.9.5. Efficacy Endpoints

Efficacy evaluation endpoints include SELENA-SLEDAI score, PGA score, time from randomization to first SLE flare, prednisone dosage, BILAG-2004 score, 24-hour urine protein quantification (only for subjects whose urine protein/creatinine ratio exceeds the ULN during screening), urine protein/creatinine ratio (only for subjects whose urine protein/creatinine ratio exceeds the ULN during screening), IgG, IgA, IgM, IgE, anti-ds-DNA antibody, complement (C3, C4), CRP, ESR, etc.

## 3.10. End of Study

Under normal circumstances, the study will be considered completed when the last subject completes the last visit. The sponsor, Ethics Committee (EC), or other regulatory authorities may also terminate the study before its completion.

## 3.11. Actions in Case of Study Interruption Due to Natural Disasters or Public Health Events

To ensure the rights and safety of subjects, compliance with Good Clinical Practice (GCP), and the integrity and continuity of the clinical study during natural disasters or public health events, the investigator or designated personnel may contact the sponsor to discuss the possibility of implementing appropriate risk mitigation plans, subject to the approval of the local health authorities, EC, or local government. These plans may include:

- Obtain informed consent or re-consent on risk mitigation.
- Perform re-screening for subjects who have been successfully screend, or extend the screening period.
- Perform home or remote visits (remote contact with subjects via telecommunication technology, including telephone, video, etc., for telemedicine visits) by qualified medical personnel.
- Complete routine examinations at an alternative institution (qualified local laboratory or imaging center, e.g., Grade II Level A hospital or above).
- IMP dispensation: For IMPs that can usually be self-administered, alternative safe methods for shipment may be adopted; for IMPs that are normally used only in a medical institution, an alternative plan for IMP administration will be communicated with regulatory authorities.

# 4. Study Procedures

## 4.1. Study Steps

### 4.1.1. V1 (D-28 to D-1, Screening Period)

Subjects who sign the ICF should complete protocol-specified screening tests/assessments within 28 days prior to the first dose to determine their eligibility for enrollment.

Subjects will maintain a stable standard of care regimen for at least 30 days before the first dose of IMP. A stable standard of care regimen refers to the stable use of any of the following (alone or in combination): corticosteroids, antimalarials, NSAIDs, immunosuppressive or immunomodulatory agents, such as azathioprine, mycophenolate (including mycophenolate mofetil, mycophenolic sodium), methotrexate, leflunomide, tacrolimus, ciclosporin. [Note: Routine dose reduction of corticosteroids ≤ 20 mg/d prednisone (or equivalent dose of other corticosteroids) is also considered stable treatment]

All study procedures to be completed at screening are as follows:

- Obtain informed consent and sign ICF;
- Confirm eligility according to inclusion/exclusion criteria;
- Collect demographic data: including age, sex, ethnicity, smoking history, alcohol consumption history, and menstrual history;
- Collect medical history, medication history, and surgical history: ① History of SLE: The date of SLE diagnosis should be recorded. ② SLE medication history: The medication use (including specific drugs/treatment, dosage, and usage, etc.) for at least 2 years prior to the screening visit should be recorded. ③ Medical history of other diseases: The occurrence of other diseases (including disease name and time of onset, etc.) within at least 6 months prior to the screening visit should be recorded. ④ Previous treatment history of other diseases: The treatment (including specific drugs/treatment, usage and dosage, etc.) within at least 1 month prior to the screening visit should be recorded. ⑤ Previous surgical history: The surgery (including name and date of surgery, etc.) within at least 6 months prior to the screening visit should be recorded.
- Measure body height and weight, and calculate body mass index (BMI);
- Perform infectious disease screening, including hepatitis b screening: hepatitis B five items (HBsAg, HBsAb, HBcAb, HBeAg, and HBeAb), and HBV-DNA if necessary; perform hepatitis c screening: HCV antibody, and HCV-RNA if necessary; HIV

screening: HIV antibody; syphilis screening: treponema pallidum antibody;

- Chest X-ray: Chest X-ray (posteroanterior and lateral) should be performed, and additional tuberculosis screening-related tests may be added according to the site's diagnostic and treatment routine to rule out active pulmonary tuberculosis;
- ANA;
- Blood type and IAT: Blood type and IAT, including ABO, Rh, and IAT, must be performed during the screening period, and subjects will be provided with an identification card indicating their blood type and IAT information;
- Vital signs: including temperature, pulse, respiration, and blood pressure;
- Physical examination: including head and face, eyes, ears, nose and throat, oral cavity, skin, lymph nodes, respiratory system, cardiovascular system, abdomen, genitourinary system (if necessary), musculoskeletal system, nervous system, and mental status;
- 12-lead ECG: including heart rate, PR interval, QRS duration, QT interval, and QTcF;
- Laboratory test: including hematology, blood chemistry, urinalysis and coagulation function;
- Blood pregnancy test (for women of childbearing potential only);
- Urine protein/creatinine ratio, 24-hour urine protein (only for subjects whose urine protein/creatinine ratio exceeds the ULN during the screening period);
- Immunology-related endpoints: including IgG, IgA, IgM, IgE, complement (C3, C4), anti-ds-DNA antibody, tested by the laboratories of each study site;
- CRP, ESR tests;
- SELENA-SLEDAI score;
- PGA score;
- Collection of prednisone dosage;
- Recording of AEs;
- Recording of concomitant therapies.

### 4.1.2. Treatment Period (D1 to D57)

The treatment period will last for 8 weeks. Subjects will complete baseline examinations at the baseline visit (D1) (safety laboratory tests before the first dose, including pregnancy test, can be completed on D1 or within 48 hours before D1), and the eligibility will be verified based on the inclusion/exclusion criteria (laboratory test results from the screening period can be used for eligibility verification). Eligible subjects will be enrolled in the study.

During the treatment period, subjects will receive intravenous infusions of the corresponding dose of CM313 or an equal volume of placebo according to the corresponding dosing frequency based on their assigned dose level.

During the treatment period, subjects will complete PK, PD, and immunogenicity blood sample collection according to the time points specified in Table 2.

During the treatment period, subjects should also complete safety tests/assessments and efficacy evaluations at the time points specified in the protocol.

#### 4.1.2.1. V2 (D1, baseline visit)

- Verify eligibility based on the inclusion/exclusion criteria;
- Measure body weight;
- ANA: If laboratory test results are available within 3 days before the first dose of IMP, the test does not need to be repeated;
- Vital signs: including temperature, pulse, respiration, and blood pressure;
- Physical examination: including head and face, eyes, ears, nose and throat, oral cavity, skin, lymph nodes, respiratory system, cardiovascular system, abdomen, genitourinary system (if necessary), musculoskeletal system, nervous system, and mental status;
- 12-lead ECG: including heart rate, PR interval, QRS duration, QT interval, and QTcF;
- Laboratory tests: including hematology, blood chemistry, urinalysis, and coagulation function; if laboratory test results are available within 3 days before the first dose of IMP, the tests do not need to be repeated;
- Pregnancy test (for women of childbearing potential only): blood pregnancy test or urine pregnancy test, can be completed on D1 or within 48 hours before D1;
- Urine protein/creatinine ratio, 24-hour urine protein: can be completed on D1 or within 48 hours before D1. If test results are available within 3 days before the first dose of IMP, the tests do not need to be repeated; 24-hour urine protein test is only required for subjects whose urine protein/creatinine ratio exceeds the ULN during the screening period;
- Immunology-related endpoints: including IgG, IgA, IgM, IgE, complement (C3, C4), anti-ds-DNA antibody, tested by the central laboratory. The results of IgG, IgA, IgM, and IgE will not be provided to each study site before unblinding to avoid breaking the blindness;

- CRP, ESR: test results within 3 days before the first dose are acceptable. Baseline examinations can be completed on D1 or within 48 hours before D1;
- SELENA-SLEDAI score: If test results are available within 3 days before the first dose of IMP, the test does not need to be repeated;
- BILAG-2004 score: If test results are available within 3 days before the first dose of IMP, the test does not need to be repeated;
- PGA score: If test results are available within 3 days before the first dose of IMP, the test does not need to be repeated;
- SLE flare assessment;
- Randomization;
- IMP administration;
- PK, PD, and immunogenicity blood sampling. Please refer to Table 2 for specific sampling time points;
- Collection of prednisone dosage;
- Recording of AEs;
- Recording of concomitant therapies;
- Distribution of subject diary cards and training of subjects on how to fill them out.

#### 4.1.2.2. V3 (D8±1d)

- Vital signs: including temperature, pulse, respiration, and blood pressure;
- Physical examination: including head and face, eyes, ears, nose and throat, oral cavity, skin, lymph nodes, respiratory system, cardiovascular system, abdomen, genitourinary system (if necessary), musculoskeletal system, nervous system, and mental status;
- 12-lead ECG: including heart rate, PR interval, QRS duration, QT interval, and QTcF;
- Laboratory test: including hematology, blood chemistry, urinalysis and coagulation function;
- PK, PD, and immunogenicity blood sampling. Please refer to Table 2 for specific sampling time points;
- Collection of prednisone dosage;
- Recording of AEs;
- Recording of concomitant therapies;
- Recovery and issuance of subject diary cards.

#### 4.1.2.3. V4 (D15±1d)

- Vital signs: including temperature, pulse, respiration, and blood pressure;
- Physical examination: including head and face, eyes, ears, nose and throat, oral cavity, skin, lymph nodes, respiratory system, cardiovascular system, abdomen, genitourinary system (if necessary), musculoskeletal system, nervous system, and mental status;
- 12-lead ECG: including heart rate, PR interval, QRS duration, QT interval, and QTcF;
- Laboratory test: including hematology, blood chemistry, urinalysis and coagulation function;
- Urine protein/creatinine ratio, 24-hour urine protein (only for subjects whose urine protein/creatinine ratio exceeds the ULN during the screening period);
- Immunology-related endpoints: including IgG, IgA, IgM, IgE, complement (C3, C4), anti-ds-DNA antibody, tested by the central laboratory. The results of IgG, IgA, IgM, and IgE will not be provided to each study site before unblinding to avoid breaking the blindness;
- CRP, ESR;
- SELENA-SLEDAI score;
- PGA score;
- SLE flare assessment;
- PK, PD, and immunogenicity blood sampling. Please refer to Table 2 for specific sampling time points;
- Collection of prednisone dosage;
- Recording of AEs;
- Recording of concomitant therapies;
- Recovery and issuance of subject diary cards.

#### 4.1.2.4. V5 (D22±1d)

- Vital signs: including temperature, pulse, respiration, and blood pressure;
- Physical examination: including head and face, eyes, ears, nose and throat, oral cavity, skin, lymph nodes, respiratory system, cardiovascular system, abdomen, genitourinary system (if necessary), musculoskeletal system, nervous system, and mental status;
- 12-lead ECG: including heart rate, PR interval, QRS duration, QT interval, and QTcF;

- Laboratory test: including hematology, blood chemistry, urinalysis and coagulation function;
- PK, PD, and immunogenicity blood sampling. Please refer to Table 2 for specific sampling time points;
- Collection of prednisone dosage;
- Recording of AEs;
- Recording of concomitant therapies;
- Recovery and issuance of subject diary cards.

#### 4.1.2.5. V6 (D29±1d)

- Measure body weight;
- Vital signs: including temperature, pulse, respiration, and blood pressure;
- Physical examination: including head and face, eyes, ears, nose and throat, oral cavity, skin, lymph nodes, respiratory system, cardiovascular system, abdomen, genitourinary system (if necessary), musculoskeletal system, nervous system, and mental status;
- 12-lead ECG: including heart rate, PR interval, QRS duration, QT interval, and QTcF;
- Laboratory test: including hematology, blood chemistry, urinalysis and coagulation function;
- Pregnancy test (for women of childbearing potential only): blood or urine pregnancy test;
- Urine protein/creatinine ratio, 24-hour urine protein (only for subjects whose urine protein/creatinine ratio exceeds the ULN during the screening period);
- Immunology-related endpoints: including IgG, IgA, IgM, IgE, complement (C3, C4), anti-ds-DNA antibody, tested by the central laboratory. The results of IgG, IgA, IgM, and IgE will not be provided to each study site before unblinding to avoid breaking the blindness;
- CRP, ESR;
- SELENA-SLEDAI score;
- BILAG-2004 score;
- PGA score;
- SLE flare assessment;
- IMP administration;
- PK, PD, and immunogenicity blood sampling. Please refer to Table 2 for specific

sampling time points;

- Collection of prednisone dosage;
- Recording of AEs;
- Recording of concomitant therapies;
- Recovery and issuance of subject diary cards.

#### 4.1.2.6. V7 (D36±1d)

- Measure body weight;
- Vital signs: including temperature, pulse, respiration, and blood pressure;
- Physical examination: including head and face, eyes, ears, nose and throat, oral cavity, skin, lymph nodes, respiratory system, cardiovascular system, abdomen, genitourinary system (if necessary), musculoskeletal system, nervous system, and mental status;
- 12-lead ECG: including heart rate, PR interval, QRS duration, QT interval, and QTcF;
- Laboratory test: including hematology, blood chemistry, urinalysis and coagulation function;
- IMP administration;
- PK, PD, and immunogenicity blood sampling. Please refer to Table 2 for specific sampling time points;
- Collection of prednisone dosage;
- Recording of AEs;
- Recording of concomitant therapies;
- Recovery and issuance of subject diary cards.

#### 4.1.2.7. V8 (D43±1d)

- Measure body weight;
- Vital signs: including temperature, pulse, respiration, and blood pressure;
- Physical examination: including head and face, eyes, ears, nose and throat, oral cavity, skin, lymph nodes, respiratory system, cardiovascular system, abdomen, genitourinary system (if necessary), musculoskeletal system, nervous system, and mental status;
- 12-lead ECG: including heart rate, PR interval, QRS duration, QT interval, and QTcF;
- Laboratory test: including hematology, blood chemistry, urinalysis and coagulation function;

- Urine protein/creatinine ratio, 24-hour urine protein (only for subjects whose urine protein/creatinine ratio exceeds the ULN during the screening period);
- Immunology-related endpoints: including IgG, IgA, IgM, IgE, complement (C3, C4), anti-ds-DNA antibody, tested by the central laboratory. The results of IgG, IgA, IgM, and IgE will not be provided to each study site before unblinding to avoid breaking the blindness;
- CRP, ESR;
- SELENA-SLEDAI score;
- PGA score;
- SLE flare assessment;
- IMP administration;
- PK, PD, and immunogenicity blood sampling. Please refer to Table 2 for specific sampling time points;
- Collection of prednisone dosage;
- Recording of AEs;
- Recording of concomitant therapies;
- Recovery and issuance of subject diary cards.

#### 4.1.2.8. V9 (D50±1d)

- Measure body weight;
- Vital signs: including temperature, pulse, respiration, and blood pressure;
- Physical examination: including head and face, eyes, ears, nose and throat, oral cavity, skin, lymph nodes, respiratory system, cardiovascular system, abdomen, genitourinary system (if necessary), musculoskeletal system, nervous system, and mental status;
- 12-lead ECG: including heart rate, PR interval, QRS duration, QT interval, and QTcF;
- Laboratory test: including hematology, blood chemistry, urinalysis and coagulation function;
- IMP administration;
- PK, PD, and immunogenicity blood sampling. Please refer to Table 2 for specific sampling time points;
- Collection of prednisone dosage;
- Recording of AEs;
- Recording of concomitant therapies;

- Recovery and issuance of subject diary cards.

#### 4.1.2.9. V10 (D57±1d, EOT visit)

- Measure body weight;
- Vital signs: including temperature, pulse, respiration, and blood pressure;
- Physical examination: including head and face, eyes, ears, nose and throat, oral cavity, skin, lymph nodes, respiratory system, cardiovascular system, abdomen, genitourinary system (if necessary), musculoskeletal system, nervous system, and mental status;
- 12-lead ECG: including heart rate, PR interval, QRS duration, QT interval, and QTcF;
- Laboratory test: including hematology, blood chemistry, urinalysis and coagulation function;
- Pregnancy test (for women of childbearing potential only): blood or urine pregnancy test;
- Urine protein/creatinine ratio, 24-hour urine protein (only for subjects whose urine protein/creatinine ratio exceeds the ULN during the screening period);
- Immunology-related endpoints: including IgG, IgA, IgM, IgE, complement (C3, C4), anti-ds-DNA antibody, tested by the central laboratory. The results of IgG, IgA, IgM, and IgE will not be provided to each study site before unblinding to avoid breaking the blindness;
- CRP, ESR;
- SELENA-SLEDAI score;
- BILAG-2004 score;
- PGA score;
- SLE flare assessment;
- PK, PD, and immunogenicity blood sampling. Please refer to Table 2 for specific sampling time points;
- Collection of prednisone dosage;
- Recording of AEs;
- Recording of concomitant therapies;
- Recovery and issuance of subject diary cards.

### 4.1.3. Follow-up Period (D58 to D113)

After the treatment period, subjects will enter an 8-week follow-up period.

Subjects will return to the study site for visits at the times specified in the protocol to undergo safety tests/assessments, efficacy evaluations, and continue to provide PK, PD, and

immunogenicity samples until the EOS visit is completed at the end of follow-up. If clinically significant abnormalities are identified, the investigator will determine whether the subject needs to undergo reexamination, treatment, or hospitalization observation and the subject will be followed up until recovery or stabilized outcome.

#### 4.1.3.1. V11 (D71±3d), V12 (D85±3d), V13 (D99±3d)

- Measure weight (only at V12, not at V11 or V13);
- Vital signs: including temperature, pulse, respiration, and blood pressure;
- Physical examination: including head and face, eyes, ears, nose and throat, oral cavity, skin, lymph nodes, respiratory system, cardiovascular system, abdomen, genitourinary system (if necessary), musculoskeletal system, nervous system, and mental status;
- 12-lead ECG: including heart rate, PR interval, QRS duration, QT interval, and QTcF;
- Laboratory test: including hematology, blood chemistry, urinalysis and coagulation function;
- Pregnancy test (for women of childbearing potential only) (only at V12, not at V11 or V13): blood or urine pregnancy test;
- Urine protein/creatinine ratio, 24-hour urine protein (only for subjects whose urine protein/creatinine ratio exceeds the ULN during the screening period);
- Immunology-related endpoints: including IgG, IgA, IgM, IgE, complement (C3, C4), anti-ds-DNA antibody, tested by the central laboratory. The results of IgG, IgA, IgM, and IgE will not be provided to each study site before unblinding to avoid breaking the blindness;
- CRP, ESR;
- SELENA-SLEDAI/PGA score;
- SLE flare assessment;
- BILAG-2004 score (only at V12, not at V11 or V13);
- PK, PD, and immunogenicity blood sampling. Please refer to Table 2 for specific sampling time points;
- Collection of prednisone dosage;
- Recording of AEs;
- Recording of concomitant therapies;
- Recovery and issuance of subject diary cards.

#### 4.1.3.2. V14 (D113±3d, EOS visit)

- Measure body weight;
- Chest X-ray: posteroanterior and lateral position;
- ANA;
- Vital signs: including temperature, pulse, respiration, and blood pressure;
- Physical examination: including head and face, eyes, ears, nose and throat, oral cavity, skin, lymph nodes, respiratory system, cardiovascular system, abdomen, genitourinary system (if necessary), musculoskeletal system, nervous system, and mental status;
- 12-lead ECG: including heart rate, PR interval, QRS duration, QT interval, and QTcF;
- Laboratory test: including hematology, blood chemistry, urinalysis and coagulation function;
- Pregnancy test (for women of childbearing potential only): blood pregnancy test;
- Urine protein/creatinine ratio, 24-hour urine protein (only for subjects whose urine protein/creatinine ratio exceeds the ULN during the screening period);
- Immunology-related endpoints: including IgG, IgA, IgM, IgE, complement (C3, C4), anti-ds-DNA antibody, tested by the central laboratory. The results of IgG, IgA, IgM, and IgE will not be provided to each study site before unblinding to avoid breaking the blindness;
- CRP, ESR;
- SELENA-SLEDAI score;
- BILAG-2004 score;
- PGA score;
- SLE flare assessment;
- PK, PD, and immunogenicity blood sampling. Please refer to Table 2 for specific sampling time points;
- Collection of prednisone dosage;
- Recording of AEs;
- Recording of concomitant therapies;
- Collection of subject diary cards.

## 4.2. Dose Interruption

During the treatment period, if the IMP is interrupted for a subject due to an AE or other reasons, the investigator may consider resumption of the IMP, directly discontinue the treatment or even withdraw from the study on a case-by-case basis. If the resumption of dosing is considered, relevant discussions should be held with the sponsor's medical monitor.

## 4.3. Early Withdrawal from the Study

If a subject withdraws from the study early, they should complete the "early withdrawal" visit as far as possible. The specific procedures, examinations, and assessments will be the same as those of the EOS visit, and blood samples for PK, PD, and immunogenicity will be collected. The sample collection time or safety examination time should be close to the time of withdrawal from the study as far as possible. At the "early withdrawal" visit, procedures, examinations and assessments will be waived if there are results within 3 days. Subjects who withdraw from the study early may be replaced following discussion between the investigator and the sponsor.

## 4.4. Unscheduled Visits

During the study, the investigator may arrange unscheduled visits based on the actual situation of each subject. Clinically significant results from procedures, examinations and assessments at unscheduled visits should be recorded in the clinical medical records and CRFs. If clinically significant abnormalities are identified, the investigator will determine whether the subject needs to undergo reexamination, treatment, or hospitalization observation and the subject will be followed up until recovery or stabilized outcome.

## 4.5. Study Termination Criteria

Study termination refers to the premature discontinuation of all study-related procedures during the clinical trial, with the primary objective of protecting the rights and interests of subjects and avoiding unnecessary harm.

Study termination will be considered if one of the following is met:

- If serious safety issues occur during the study, the study should be terminated in a timely manner;
- Major errors in the study protocol are found during the study, or important deviations occur in the implementation, which make the study endpoints difficult to evaluate;
- Termination requested by the sponsor (e.g., for reasons of funding and management);

- Study termination required by administrative authorities or the ECs;
- Other reasons.

Both the investigator and the sponsor have the right to terminate the study at any time. If it is necessary to terminate, it shall be implemented after consultation and consensus are reached between the two parties. In terminating the study, the sponsor and the investigator will assure that the interests of the subjects are adequately protected.

### 4.6. Pregnancy/Lactation and Contraceptive Requirements

A blood pregnancy test will be performed at screening and at EOS for female subjects of childbearing potential. During the study, a pregnancy test will be performed at the time specified in the procedure, and a blood or urine pregnancy test will be performed at the discretion of the investigator.

Female subjects and their partners must agree to use highly effective contraception methods (see Appendix 6) from the time of signing the ICF until 4 months after the last dose of IMP, and male subjects and their partners must agree to use highly effective contraception methods from the time of signing the ICF until 6 months after the last dose of IMP.

Women who have a positive blood pregnancy test at screening are not eligible for enrollment. If a female subject becomes pregnant while receiving the IMP, she will be withdrawn from the study immediately. If a female subject or the partner of a male subject becomes pregnant, the investigator should report it to the sponsor immediately. The investigator should obtain the result of pregnancy and report the condition of the fetus or newborn child to the sponsor.

# 5. Methods of Assessment

## 5.1. Routine Assessments

Demographic data: The subjects' age, sex, ethnicity, smoking history, alcohol consumption history and menstrual history will be inquired and recorded at screening.

Medical, surgical and medication history: ① History of SLE: The date of SLE diagnosis will be recorded. ② SLE medication history: The medication use (including specific drugs/treatment, dosage, and usage, etc.) for at least 2 years prior to the screening visit should be recorded. ③ Medical history of other diseases: The occurrence of other diseases (including disease name and time of onset, etc.) within at least 6 months prior to the screening visit should be recorded. ④ Previous treatment history of other diseases: The treatment (including specific drugs/treatment, usage and dosage, etc.) within at least 1 month prior to the screening visit should be recorded. ⑤ Previous surgical history: The surgery (including name and date of surgery, etc.) within at least 6 months prior to the screening visit should be recorded.

Body height, weight, BMI: Body height (cm) and weight (kg) will be measured during the screening period V1 (D-28 to D-1), and BMI will be calculated based on body height and weight. Only weight will be measured at visits V2, V6, V7, V8, V9, EOT, V12, and EOS.

Infectious disease screening: This will be conducted during the screening period V1 (D-28 to D-1) and includes: hepatitis B screening: hepatitis B five items (HBsAg, HBeAg, HBsAb, HBcAb, HBeAb, and HBV-DNA if necessary); hepatitis C screening: HCV antibody (HCV-RNA if necessary); HIV screening: HIV antibody; tuberculosis screening: chest X-ray (posteroanterior and lateral position), additional tuberculosis screening-related tests may be added according to the study site's diagnostic and treatment routine; syphilis screening: syphilis spirochete antibody.

## 5.2. Safety Assessments

All samples will be collected by authorized study personnel. During the study, the site will review the test results based on the latest reference ranges (including laboratory tests and non-laboratory tests such as physical examination, vital signs,12-lead ECG, etc.) and identify results outside the normal range. The investigator should assess whether the abnormal results obtained are clinically significant.

### 5.2.1. Physical Examination

Physical examination includes head and face, eyes, ears, nose and throat, oral cavity, skin, lymph nodes, respiratory system, cardiovascular system, abdomen, genitourinary system (if necessary), musculoskeletal system, nervous system, and mental status. It will be conducted at V1 (screening visit), V2 (baseline visit), V3, V4, V5, V6, V7, V8, V9, V10 (EOT visit), V11, V12, V13, and V14 (EOS visit).

Unscheduled physical examinations may be performed as deemed necessary by the investigator. Abnormalities of physical examination judged by the investigator to be clinically significant should be recorded as AEs.

### 5.2.2. Vital Signs

Vital signs include temperature, pulse, respiration, blood pressure. They should be measured at V1 (screening visit), V2 (baseline visit), V3, V4, V5, V6, V7, V8, V9, V10 (EOT visit), V11, V12, V13, and V14 (EOS visit).

Subjects should rest for at least 5 min before vital signs are measured. Pulse and blood pressure should be measured with the same arm whenever possible throughout the study.

In the event of abnormal blood pressure measurements, triplicate measurements (at least 5 min apart between two measurements) should be averaged. Additional measurement of vital signs may be performed as deemed necessary by the investigator. Abnormal vital sign findings judged by the investigator to be clinically significant should be recorded as AEs.

### 5.2.3. 12-lead ECG

12-lead ECG includes heart rate, PR interval, QRS duration, QT interval, and QTcF [QTcF = QT/(RR^0.33)]. It will be conducted at V1 (screening visit), V2 (baseline visit), V3, V4, V5, V6, V7, V8, V9, V10 (EOT visit), V11, V12, V13, and V14 (EOS visit).

If blood samples need to be collected on the day of 12-lead ECG, the 12-lead ECG should be performed before blood collection or at least 20 minutes after the completion of blood collection. Subjects should rest for at least 5 min prior to examination.

Additional 12-lead ECGs may be performed as deemed necessary by the investigator. Abnormal 12-lead ECG findings judged by the investigator to be clinically significant should be recorded as AEs.

#### 5.2.4. Laboratory Tests

Laboratory tests include hematology, blood chemistry, urinalysis, and coagulation function. See Section 3.9.1 for specific details. It will be conducted at V1 (screening visit), V2 (baseline visit), V3, V4, V5, V6, V7, V8, V9, V10 (EOT visit), V11, V12, V13, and V14 (EOS visit). During the course of the study, the frequency of clinical laboratory tests may be increased if the investigator deems that it is necessary for safety considerations. Abnormal laboratory results judged by the investigator to be clinically significant should be reported as AEs.

#### 5.2.5. Adverse Events

All AEs occurring in all subjects after signing the ICF until the end of the follow-up period will be monitored, with their clinical characteristics, severity, time of onset, end time (if applicable), treatment measures, and outcome recorded, and their causal relationship with the IMP will be determined.

#### 5.2.6. Pregnancy Test

Pregnancy test is for women of childbearing potential only. It will be performed at V1 (screening visit), V2 (baseline visit), V6, V10 (EOT visit), V12, and V14 (EOS visit). Blood pregnancy test will be performed at screening visit and EOS visit, while blood or urine pregnancy tests will be performed at other visits.

Additional blood or urine pregnancy tests may be performed as deemed necessary by the investigator.

### 5.3. Efficacy Evaluation

#### 5.3.1. SELENA-SLEDAI Score

SELENA-SLEDAI is a validated SLE activity index system used to measure disease activity in SLE patients over the past 10 days, primarily evaluating the presence of 24 clinical characteristics across 9 organ systems. In this index system, the presence of different clinical characteristics is assigned corresponding weighted scores, as detailed in Appendix 2. The total SELENA-SLEDAI score is the sum of the weighted scores corresponding to the 24 clinical characteristics, with a total score range of 0 to 105. The SELENA-SLEDAI score will be assessed by the evaluating physician, with higher total SELENA-SLEDAI scores indicating more severe disease.

SELENA-SLEDAI scores will be assessed at V1 (screening visit), V2 (baseline visit), V4, V6, V8, V10 (EOT visit), V11, V12, V13, and V14 (EOS visit).

### 5.3.2. SLE Flare/BILAG-2004/PGA Score

The SLE Flare Index (see Appendix 3) is used to detect SLE flares and distinguish between mild to moderate flares and severe flares.

BILAG-2004 (see Appendix 4) is a validated SLE disease activity index that assesses changes in the patient's clinical manifestations over the past 4 weeks compared to the previous 4 weeks (not present, improving, same, worse, or new), based on the physician's treatment intent, including 97 clinical signs, symptoms, and laboratory parameters across 9 organ systems. A five-level system is used to assess the degree of disease change in all 9 organ systems: A (severe), B (moderate), C (mild), D (inactive), E (inactive with no previous involvement).

The PGA score (see Appendix 5) is the physician's assessment of the overall disease activity in SLE patients over the past 2 weeks, using a visual analog scale to assess disease activity. SLE patients will undergo PGA assessment, with 0 points representing no activity, 1 point representing mild activity, 2.0 to 2.5 points representing moderate activity, and 3 points representing severe activity.

The number of SLE flares and the severity of each flare, as well as the date of each flare, will be recorded at V2 (baseline visit), V4, V6, V8, V10 (EOT visit), V11, V12, V13, and V14 (EOS visit), with the severity of the flare referring to the SLE Flare Scoring Index.

BILAG-2004 scores will be assessed at V2 (baseline visit), V6, V10 (EOT visit), V12, and V14 (EOS visit).

PGA scores will be assessed at V1 (screening visit), V2 (baseline visit), V4, V6, V8, V10 (EOT visit), V11, V12, V13, and V14 (EOS visit).

### 5.3.3. Urine Protein/Creatinine Ratio, 24-hour Urine Protein

SLE can affect the kidneys. As the disease progresses, the urine protein/creatinine ratio and 24-hour urine protein tend to increase. Therefore, the urine protein/creatinine ratio or 24-hour urine protein is an indicator reflecting the outcome of SLE.

Urine protein/creatinine ratio will be tested at V1 (screening visit), V2 (baseline visit), V4, V6, V8, V10 (EOT visit), V11, V12, V13, and V14 (EOS visit).

24-hour urine protein will be tested at V1 (screening visit), V2 (baseline visit), V4, V6, V8, V10 (EOT visit), V11, V12, V13, and V14 (EOS visit) only for subjects whose urine protein/creatinine ratio exceeds the ULN during the screening period.

### 5.3.4. Prednisone Dosage

Glucocorticoids are one of the basic drugs for SLE treatment. The dosage of glucocorticoids increases with the severity of the disease. Therefore, the dosage of glucocorticoids is an indicator reflecting the outcome of SLE.

Prednisone dosage will be recorded at V1 (screening visit), V2 (baseline visit), V3, V4, V5, V6, V7, V8, V9, V10 (EOT visit), V11, V12, V13, and V14 (EOS visit).

### 5.3.5. IgG, IgA, IgM, IgE, Anti-ds-DNA Antibody, Complement (C3, C4), CRP, ESR

Serological indicators IgG, IgA, IgM, IgE, anti-ds-DNA antibody, complement (C3, C4), CRP, and ESR can reflect the disease activity of SLE. Based on their dynamic changes, they can assist in judging the outcome of SLE.

IgG, IgA, IgM, IgE, anti-ds-DNA antibody, complement (C3, C4), CRP, and ESR will be tested at V1 (screening visit), V2 (baseline visit), V4, V6, V8, V10 (EOT visit), V11, V12, V13, and V14 (EOS visit).

Samples for IgG, IgA, IgM, IgE, anti-ds-DNA antibody, and complement (C3, C4) from baseline and subsequent visits need to be sent to the central laboratory. A separate Sample Management Manual will detail the sampling, storage, and transportation procedures that should be followed in this study. The sponsor will provide the Sample Management Manual before the start of the study. A total of 2 mL of venous blood will be drawn for each of the 6 items of IgG, IgA, IgM, IgE, and complement (C3, C4), and 2 mL of venous blood will be drawn for anti-ds-DNA antibody each time. The results of IgG, IgA, IgM, IgE at baseline and subsequent visits will not be provided to the study sites before unblinding to avoid breaking of blindness.

## 5.4. Pharmacokinetic Evaluation

### 5.4.1. Pharmacokinetic Sample Collection

Blood samples will be collected for PK analysis. The sampling schedule is shown in Table 2.

### 5.4.2. Pharmacokinetic Sample Processing

Four mL of venous blood will be collected before the first dose of IMP, and 2 mL of venous blood will be collected each time thereafter. The serum will be separated and sent to the central laboratory for testing.

A separate Sample Management Manual will specify the sampling, storage, and transportation procedures to be followed for this study, and the sponsor will provide a Sample Management Manual prior to the start of the study.

## 5.5. Pharmacodynamic Evaluation

### 5.5.1. Biomarker Sample Collection

Blood samples will be collected for immune cell phenotyping analysis (including T lymphocytes (CD3/CD4/CD8/CD38), B lymphocytes (CD19/CD138/CD27/CD38), pDC cells (CD123/BDCA2/CD38), and NK cells (CD56/CD16/CD38)) and type I IFN-related gene expression. The sampling time is shown in Table 2.

### 5.5.2. Biomarker Sample Processing

For immune cell phenotyping analysis, 3 mL of venous blood will be collected each time. For type I IFN-related gene expression, 3 mL of venous blood will be collected each time. All blood samples need to be sent to the central laboratory for testing after collection.

A separate Sample Management Manual will specify the sampling, storage, and transportation procedures to be followed for this study, and the sponsor will provide a Sample Management Manual prior to the start of the study.

## 5.6. Immunogenicity Evaluation

### 5.6.1. Immunogenicity Sample Collection

Blood samples will be collected to assess ADA. The sampling time is shown in Table 2.

### 5.6.2. Immunogenicity Sample Processing

At each time, 3 mL of venous blood will be collected. After serum separation, the samples will be sent to the central laboratory for testing.

A separate Sample Management Manual will specify the sampling, storage, and transportation procedures to be followed for this study, and the sponsor will provide a Sample Management Manual prior to the start of the study.

# 6. Safety Assessments

## 6.1. Adverse Events

### 6.1.1. Definition of Adverse Event

An AE is defined as any untoward medical event the occurs after the subject signs the ICF, which may be manifested as symptoms, signs, diseases, or laboratory test abnormal, but may not necessarily have a causal relationship with the IMP.

### 6.1.2. Recording of Adverse Events

All AEs will be collected from the signing of the ICF until the end of the follow-up period, regardless of their severity or relationship to the IMP, and should be recorded on the corresponding pages of the original medical record and eCRF.

When recording AEs, AEs should be described as much as possible with a single diagnosis or syndrome rather than a symptom. The investigator should record in detail the start date (the date of the first occurrence of AE-related symptoms) and end date of AEs, AE severity, assessment of the relatedness of the AE to the IMP or study procedures, actions taken with the IMP, treatment given for the AE, and outcome of the AE, etc.

To ensure the safety of the subject, the investigator should take appropriate measures to track all AEs (including adverse medical events from signing the ICF until the first dose) until the AE resolves, stabilizes, has an alternative explanation, or the subject is lost to follow-up. This means that observations may last beyond the last visit as specified in the protocol.

### 6.1.3. Severity

The investigator should assess the severity of AEs according to the following criteria:

- Mild: Events that require minimal treatment or no treatment and do not interfere with the subject’s daily activities.
- Moderate: Events leading to minor inconvenience or requiring treatment. Moderate events may cause some interference with body function.
- Severe: Events that interfere with the daily activities of the subject and may require systemic drug therapy or other treatment. Severe events are usually potentially life-threatening or incapacitating. It should be noted that the term "severe" does not necessarily equate to "serious".

#### 6.1.4. Causality

The causality between AE and the IMP is determined by the investigator based on the following criteria:

| Relatedness | Description |
|---|---|
| Definitely related | There is a reasonable temporal sequence between the occurrence of an AE and the use of the IMP. The AE is a known adverse reaction of the IMP, which is alleviated or disappeared after drug withdrawal, and recurs after drug re-administration, and cannot be explained by the subject's own disease. |
| Probably related | There is a reasonable temporal sequence between the occurrence of AE and the use of the IMP. The AE is a known adverse reaction of the IMP, which is alleviated or disappeared after discontinuation of the IMP and cannot be explained by the subject's own disease, and the result after readministration is unknown. |
| Possibly related | There is a reasonable temporal sequence between the occurrence of AE and the use of the IMP. AE is a known or suspected adverse reaction of the IMP, but there are other factors that may cause the event, such as diseases, and concomitant medications. The result of dechallenge is unknown, unclear, or lacks conclusive information. |
| Unlikely related | There is a reasonable temporal sequence between the occurrence of AE and the use of the IMP, but the event is not of a known type of adverse drug reaction and is most likely caused by the subject's disease or other treatment. |
| Not related | There is no reasonable temporal sequence between the occurrence of AE and the use of the IMP, e.g., the event has occurred before the use of the IMP; the event is not a known adverse drug reaction; or the event is indeed caused by other factors, such as the subject's disease, other treatments and concomitant medications. |

### 6.2. Serious Adverse Events

#### 6.2.1. Definition of Serious Adverse Event

An SAE is defined as any untoward medical occurrence that at any dose meets any of the following criteria:

- Leading to death;
- Life-threatening circumstances, which refer to an immediate risk of death in a seriously affected patient, rather than a speculated death that may occur in the future if the condition gets serious;
- Requiring hospitalization or prolongation of hospitalization;
- Permanent or significant disability/incapacity;
- Teratogenic effect, leading to birth defect;
- Other medically significant events: Medical and scientific judgment must be used to determine whether expedited reporting of other events is required; in general, any medically significant event that may not be immediately life-threatening, or leading

to death, or resulting in hospitalization, but for which medical interventions are required to prevent any of the above from happening, is also considered an SAE.

**Suspected unexpected serious adverse reactions (SUSARs):**

A SUSAR is defined as a suspected and unexpected serious adverse reaction of which nature and severity of clinical manifestations are more serious than that described in the available materials, such as the Investigator's Brochure of test drug, and the package insert or summary of product characteristics of a marketed drug.

### 6.2.2. Reporting of Serious Adverse Events

From the signing of the ICF until the end of the follow-up period, for all SAEs that occur, regardless of their relationship to the IMP, the investigator will immediately take timely and appropriate treatment measures for the subject (if applicable), completely and promptly record all relevant information with as much detail as possible, fill in the SAE report form provided by the sponsor, complete all applicable sections of the form, and report in writing to the sponsor and its designated contact person within 24 hours of becoming aware of the event (including becoming aware of new follow-up information). Follow-up information will be provided in a new SAE report form, labeled as follow-up information for previously reported SAEs, and reported to the sponsor and its designated contact person within 24 hours of awareness. Identification code of the subject in the clinical study should be indicated in the SAE report and follow-up report, instead of identity information such as the subject's real name, citizen ID number and address. For reports involving death events, the investigator should provide the sponsor and the EC with other information required, such as autopsy report and final medical report. For SAEs that occur from the end of the clinical trial or follow-up to the time when the conclusion of review and approval is obtained, the investigator should also notify the sponsor within 24 hours of awareness (the reporting process is detailed in the corresponding safety document).

SAEs that have not recovered at the end of the study or at the time of early withdrawal must be followed up until one of the following is achieved:

The event is recovered; or

The event becomes stable; or

The event returns to baseline level (if baseline records are available); or

The event can be attributed to medications other than the IMP or factors unrelated to the behavior of the investigator, or when more information is unlikely to be available (the subject

or healthcare provider refuses to provide further information, or there are evidences proving that the subject is lost to follow-up even after the best efforts have been made).

Upon receiving relevant clinical trial safety information from the sponsor, the investigator should promptly sign and acknowledge receipt, read the information, and consider whether any adjustments to the subject's treatment are necessary. If needed, the investigator should communicate with the subject as early as possible and report the SUSAR provided by the sponsor to the EC.

## 6.3. Other Safety Information

### 6.3.1. Abnormal Laboratory Results

Abnormal laboratory test results that occur during the study will be determined by the investigator based on the subject's symptoms, signs, etc. Abnormal and clinically significant laboratory results will be recorded as AEs.

### 6.3.2. Overdose

Any overdose occurring in the study, whether related to an AE or not, must be reported to the CRA or to the sponsor-designated medical monitor and documented in the eCRF. Any AE associated with an overdose should be recorded on the appropriate AE/SAE page in the eCRF.

### 6.3.3. Pregnancy

Pregnancy itself is not considered as an AE. However, pregnancies of female subjects that occur between signing the ICF and 4 months after the last dose, and pregnancies of partners of male subjects that occur between signing the ICF and 6 months after the last dose, will be reported by the investigator to the sponsor and its designated contact person, and EC (if applicable) in the form of pregnancy reports within 24 hours of awareness of the pregnancy event. Subjects or subject's partners should also be followed up until the end of pregnancy/termination of pregnancy (abortion spontaneous, abortion induced, normal delivery, or congenital malformation), and the follow-up information should be recorded in the pregnancy report form and reported to the sponsor and its designated contact person and EC (if applicable).

All congenital malformations/birth defects are SAEs. Spontaneous abortion, or termination of pregnancy for medical reasons, should also be considered as an SAE and reported according to the SAE reporting procedure. Induced abortion without any complications should not be managed as an AE. Hospitalization for a normal birth of a healthy infant should not be

considered as an SAE. The outcome of all pregnancy events should be reported to the sponsor and its designated contact person, and EC (if applicable).

# 7. Data Management

## 7.1. Data Completion Requirements for Investigators

The investigator should collect the data required for clinical trial in accordance with the protocol requirements and ensure that all clinical trial data are obtained from the source documents and trial records, and are accurate, complete, readable and timely. The data in the eCRF should be consistent with the source documents. If any inconsistencies exist, a reasonable explanation should be provided.

All the items in the study records should be filled in, with no empty or missing items.

The modifications to source data should be traceable, the initial data should not be covered, and the reason and time of the modification should be recorded.

## 7.2. Data Monitoring Requirements for Monitors

All subjects should be checked for the signing of ICF before participating in the study, and the screening and enrollment should be verified.

In case of early withdrawal of subjects, it should be checked whether the withdrawal information has been fully and accurately recorded.

It is required to check if the data specified in the protocol are accurately recorded in the eCRF, whether the eCRF is filled in completely and accurately, and consistent with the source documents.

Whether the errors or omissions in all paper source have been corrected or noted, as well as signed and dated by the investigator should be checked.

Whether all AEs have been documented and SAEs have been reported and documented should be confirmed.

Whether the IMP has been supplied, stored, dispensed and returned in accordance with relevant regulations, and whether the corresponding records are true and complete should be verified.

## 7.3. Data Management Requirements

### 7.3.1. Case Report Form Design

The eCRFs will be used for this study. The eCRFs are designed by data manager according to study procedures and flowchart specified in the protocol, and the design should ensure that all data collected can meet the statistical analysis. The eCRFs should be reviewed jointly by the sponsor or contract research organization (CRO) entrusted by the sponsor, the clinical personnel, medical monitor and statistician, etc., to ensure compliance with the protocol and

relevant laws and regulations. The design, approval and version control process of eCRFs needs to be fully documented.

### 7.3.2. Database Design

An EDC system will be used for the collection and management of study data in this study. The database establishment shall be set up in accordance with standard operating procedures. After the database is established, it shall be tested, and the database test report shall be issued. The database can be released for use only after it passes the test and gets approved.

### 7.3.3. Electronic Case Report Form Completion Guidelines

The eCRF completion guidelines are specific instructions for each form and each data point of the eCRF according to the protocol. It should be ensured that the study site receives the eCRF and its completion guidance before enrolling subjects, and that relevant personnel at the study site are trained on the EDC system usage process and eCRF completion. The training process should be archived.

### 7.3.4. Case Report Form Completion

The original records shall be accurately, timely, completely and normatively recorded into the eCRF by authorized study personnel in accordance with the eCRF completion guidelines. Modifications to the eCRFs should be the traceable.

### 7.3.5. Data Review and Query Management

Data verification includes computerized program edit check, manual review and data verification meeting. Edit checks are integrated into the electronic database and data queries pop out in real time. During the study, data managers, medical monitor, etc., also need to manually check the data, and issue the online queries if any doubt arises. The investigator should verify the data according to the query, and modify the data or respond to the data queries based on the queries received, and the data manager, medical monitor, etc. should review the modified data or query response, and issue queries again if necessary until all data are cleaned. After the data entry is completed and data is cleaned up, the principal investigator should conduct a final review and give electronic signature to ensure that the data records are accurate and logical. If the data is revised after signature, it needs to be re-signed.

#### 7.3.6. Medical Coding

The medical history, AEs, prior and concomitant medications and other data needing coded collected in clinical trials should be coded using standard dictionaries. The dictionary and its version used for coding should be clearly documented for the coded dataset.

#### 7.3.7. Consistency Comparison of Serious Adverse Events

All SAE-related data points in the EDC system should be compared with the safety database for consistency according to the reconciliation plan.

#### 7.3.8. Data Review Meetings

Before the data review meeting, a draft data review report should be prepared. The sponsor, investigator, data management personnel, medical personnel, statistician and others will jointly review the data and verify the AE/SAE reporting and handling records according to the clinical trial protocol. The data review report should be finalized after the data review meeting.

#### 7.3.9. Database Lock and Unlock

Database lock is an important milestone in the clinical study process. The locking process and time shall be clearly documented. Locking is to cancel the permission to edit the database, so that the data cannot be edited by any account.

If any modifications are needed after the database is locked, an application must be submitted. The modifications can only be implemented after joint discussion and signature confirmation by the sponsor, data management personnel, medical personnel, and statistician. The reasons for unlocking the database should be recorded in detail.

# 8. Statistical Considerations

The safety evaluation, efficacy evaluation, PK evaluation, PD evaluation, immunogenicity evaluation, and other analyses of this study will be specified in detail in a separate SAP. The SAP will be finalized before database lock.

## 8.1. Sample Size Estimation

The sample size of this study is not based on statistical calculations. It is planned to enroll 40 SLE subjects, with 10 subjects in each of the 2 mg/kg, 4 mg/kg, 8 mg/kg, and 16 mg/kg dose levels. At each dose level, 8 subjects will receive CM313 and 2 subjects will receive placebo.

## 8.2. Analysis Sets

**Full Analysis Set (FAS):** It includes all randomized subjects who have received at least one dose of IMP.

**Per-Protocol Set (PPS):** It is a subset of the FAS, including all subjects in the study who complete treatment with IMP as required by the protocol, have no major protocol deviations, and have available efficacy endpoints, and do not affect the efficacy evaluation. The PPS should satisfy at least the following criteria:

- Meeting the inclusion criteria specified in the study protocol;
- Meeting no exclusion criteria specified in the study protocol;
- Receiving no medications or treatments that may affect the efficacy evaluation during the study;
- Good compliance.

**PK Concentration Set for the First Dose (PKCS1):** It includes all subjects who have completed the first dose of IMP and have at least one valid post-first dose concentration data.

**PK Parameter Set for the First Dose (PKPS1):** It includes all subjects who have completed the first dose of IMP and have at least one valid post-first dose PK parameter.

**PK Concentration Set for the Last Dose (PKCS2):** It includes all subjects who have completed all doses of IMP and have at least one valid post-last dose concentration data.

**PK Parameter Set for the Last Dose (PKPS2):** It includes all subjects who have completed all doses of IMP and have at least one valid post-last dose PK parameter.

**PD Set (PDS):** It includes all subjects who have received at least one dose of IMP and have one valid PD data.

**Immunogenicity Set (IMGS):** It includes all subjects who have received at least one dose of IMP and have one valid post-dose immunogenicity data.

**Safety Set (SS):** It includes all subjects who have received at least one dose of IMP.

The above analysis sets will be discussed and decided jointly by the principal investigator, sponsor, statistician and data management personnel in the blind data review meeting before database lock.

## 8.3. Statistical Analyses

### 8.3.1. General Principles

Continuous variables will be summarized by mean, median, standard deviation, maximum and minimum; categorical or ordinal variables will be summarized by count and percentage.

All statistical analysis will be performed using SAS v9.4 statistical software.

### 8.3.2. Subject Disposition

The number of subjects who have been screened, have failed the screening, have been randomized into each dose level, completed treatment, and completed the study, as well as the number of subjects included in each analysis set will be summarized. Separate analyses will be conducted on the reasons for premature treatment discontinuation and withdrawal from the study.

### 8.3.3. Demographics and Baseline Characteristics

Descriptive statistics will be used to summarize demographics and baseline characteristics.

### 8.3.4. Efficacy Evaluation

Descriptive statistics will be performed on the SELENA-SLEDAI score, PGA score, prednisone dosage, BILAG-2004 score, 24-hour urine protein quantification (only for subjects with urine protein/creatinine ratio exceeding the ULN during screening), urine protein/creatinine ratio (only for subjects with urine protein/creatinine ratio exceeding the ULN during screening), and their changes from baseline, as well as immunology-related endpoints, CRP, ESR, and their percentage change from baseline at each visit after treatment for each group.

The Kaplan-Meier method will be used to estimate the first-flare rate of SLE at different time points in each group, as well as the 25th percentile, median, and 75th percentile of the time to first-flare of SLE and their 95% confidence intervals (Brookmeyer-Crowley method). Kaplan-Meier survival curves will also be plotted.

### 8.3.5. Pharmacokinetic Evaluation

Descriptive statistics of serum concentrations at different time points for each dose level will be performed by the scheduled sampling time points, including the number of subjects, arithmetic mean, standard deviation, coefficient of variation, median, minimum, maximum, geometric mean and geometric coefficient of variation. Linear as well as semi-logarithmic plots of mean serum concentrations over time should be plotted for each dose level by scheduled sampling time points. Linear as well as semi-logarithmic plots of serum concentrations over time should be plotted for individual subjects by actual sampling time points.

PK parameters after the first dose and the last dose will be calculated using a non-compartmental model. Descriptive statistics will be performed for each PK parameter at each dose level.

The dose linearity of $C_{max}$, $AUC_{0-t}$, and $AUC_{0-\infty}$ (for the last dose only) will be assessed using the Power Model for the first dose and the last dose, respectively. A linear regression model will be fitted using log-transformed PK parameters as dependent variables and log-transformed doses as independent variables, and the slope and its 90% confidence interval (CI) will be estimated.

### 8.3.6. Pharmacodynamic Evaluation

Descriptive statistics will be performed on the PD endpoints at each evaluation time point after treatment in each group, as well as their changes and percentage changes from baseline.

### 8.3.7. Immunogenicity Evaluation

Descriptive statistics will be performed on ADA results at each evaluation time point for each group, and the number and percentage of ADA positive subjects in each group will be calculated.

### 8.3.8. Safety Evaluation

#### 8.3.8.1. Drug Exposure

Descriptive statistics will be performed on study duration, treatment duration, times of administration, and compliance, and compliance will be summarized categorically by <80%, 80%-120%, and >120%. Among them, study duration and exposure duration are calculated as follows:

Study duration (days) = date of study completion - date of signing the ICF + 1, where the date of withdrawal will replace the date of study completion for subjects who withdraw from the study early.

Treatment duration (days) = (date of last IMP injection - date of first IMP injection) + 1 day.

Compliance will be calculated by drug exposure, i.e., compliance =(actual total dosage/planned total dosage)×100%.

#### 8.3.8.2. Adverse Events

AEs will be coded using the Medical Dictionary for Regulatory Activities (MedDRA) and classified by SOC and PT. The analysis of AE in this study will mainly focus on TEAEs (defined as any AE that newly appears after treatment, or any AE that is present before treatment but worsens in severity after treatment). AEs that occur before treatment will be listed. Drug-related is defined as the relationship of an AE to IMP as "definitely related", "probably related", or "possibly related".

The number of events, number of subjects, and percentage of the following AEs will be calculated for each group by SOC and PT: all TEAEs, IMP-related TEAEs, severe TEAEs, IMP-related severe TEAEs, SAEs, IMP-related SAEs, TEAEs leading to treatment discontinuation, IMP-related TEAEs leading to treatment discontinuation, TEAEs leading to early withdrawal, IMP-related TEAEs leading to early withdrawal, TEAEs leading to death, and IMP-related TEAEs leading to death.

#### 8.3.8.3. Laboratory Tests

Descriptive statistics will be performed for laboratory results in the form of shift tables to compare baseline and post-baseline results (based on the normal range and investigator's judgment of clinical significance).

#### 8.3.8.4. Vital Signs

Descriptive statistics will be performed on vital sign variables and their changes from baseline at each post-treatment evaluation time point in each group.

#### 8.3.8.5. Physical Examination

Descriptive statistics will be performed for physical examination results in the form of shift tables to compare baseline and post-baseline results (based on investigator's judgment of clinical significance).

#### 8.3.8.6. 12-lead ECG

Descriptive statistics will be performed for 12-lead ECG findings in the form of shift tables to compare baseline and post-baseline results (based on the normal range and the investigator's judgment of clinical significance).

### 8.3.9. Subgroup Analysis

No subgroup analysis is planned for this study.

### 8.3.10. Interim Analysis

No interim analysis is planned for this study.

### 8.3.11. Multiplicity Issues

Multiplicity issues are not addressed in this study.

### 8.3.12. Handling of Missing Data

The rules for handling missing data in the efficacy data of this study are detailed in the SAP. Missing data in PK, PD, immunogenicity, and safety data will not be handled.

# 9. Quality Control and Assurance

## 9.1. Monitoring

The sponsor has a moral, legal, and scientific obligation to ensure that the study is conducted in accordance with established study guidelines, GCP guidelines, applicable regulatory requirements, and regulations. As a coordinated measure to fulfill these obligations, in the study, the sponsor's monitor or his/her representative should visit the study site on a regular basis, in addition to frequent telephone and written communication with the study site. In order to assess subject enrollment, protocol compliance, completeness and accuracy of CRF entry, validation of CRF data and source documents, and occurrence of AEs, on-site monitoring, telephone inquiries, and periodic CRF reconciliation will be performed. The investigator must provide the monitor with access to all source materials and study documents.

The study site must complete the CRF in a timely manner and allow periodic reconciliation by the study monitor.

## 9.2. Audit and Inspection

Site audits may be conducted by the sponsor or its representatives. Audits include, but are not limited to, IMP supply, completeness of required documents, informed consent process, and

consistency of eCRFs to source documents. The investigator agrees and participates in audits conducted in a reasonable manner and at a reasonable time.

Sites may be inspected by regulatory authorities during or after the end of the study. Once an inspection has been performed, the investigator should contact the sponsor/CRO immediately. The investigator should fully cooperate with the inspection performed at a reasonable time and in a reasonable manner.

The purpose of the audit is to confirm that the study is accordance with ethical, regulatory and quality requirements.

## 9.3. Quality Control

All personnel participating in the study should be trained regarding the protocol prior to the start of the clinical study. The investigators should read and understand the contents of the study protocol, understand the principles of GCP, unify recording methods and judgment standards, and strictly follow the protocol.

Quality control must be performed by qualified designee. Quality control will be performed at participating sites during the study. If special problems are identified in some sites, corrective actions should be established.

# 10. Ethics

## 10.1. Ethics Committee

This study will be conducted in accordance with EC, GCP, ICH-GCP E6 (R2) guidelines and in compliance with applicable regulatory and data protection requirements.

Written, dated approval/favorable comment of the EC for protocol/protocol amendments, ICFs, other updated ICFs, subject recruitment process (e.g., recruitment advertisements), and other written materials provided to subjects must be obtained prior to the initiation of the study. In the EC approval letter, the version number of the protocol and the name of each document reviewed should be specified.

The EC shall keep all records of ethical review, including its written records, information of members, submitted documents, meeting minutes and relevant correspondence, etc. All records should be retained for at least 5 years after the end of this clinical study. The investigator, sponsor or drug regulatory authority may request the EC to provide its standard operating procedures and a list of ethical review members.

## 10.2. Ethical Guidance for the Study

This study will be conducted in strict compliance with the protocol, the Declaration of Helsinki, GCP, ICH-GCP E6 (R2), and all other applicable laws and regulations.

## 10.3. Informed Consent of the Subjects

The investigator will explain the benefits and risks of study participation to each subject, the subject's guardian, or an impartial witness, and obtain the written informed consent. Written informed consent must be obtained before the subject enters the study and any study-related procedures are conducted, including the use of IMP.

The original ICF signed and dated by the subject or his/her guardian and the relevant person conducting the informed consent discussion will be retained by the investigator as the study data. The investigator will provide the signed ICFs to all subjects.

During the study, the ICF may be revised after new and important data related to subject safety become available. In this case, EC approval is required and the investigator should communicate with the subject who has previously signed the ICF with the updated information, and obtain the written informed consent again. This process will also be documented.

With the consent of the subject, the investigator may inform relevant clinician of the subject's participation in the study.

# 11. Study Management and Materials

## 11.1. Electronic Case Report Form

Electronic data management will be used in this study. The eCRFs will be designed by the data management team in the EDC system according to the protocol, and can only be released for use after passing tests and obtaining approval. The eCRFs will be used to store and transmit subject information.

Permissions will be managed in the EDC system by setting role and assigning password. Investigators or designated personnel will enter data into the database in a timely, complete, and standardized manner after the completion of the original records. If data needs to be changed due to erroneous input or other reasons, an electronic audit trail will track these changes. If it is necessary for audit or inspection, the corresponding personnel will be given corresponding authority with the approval of the sponsor.

After the data entry is completed and the data is cleaned, the investigator will review and confirm by electronic signature. If the data is revised after signature, it needs to be re-signed for confirmation.

## 11.2. Data Collection

At each study visit, the study doctor will record the disease course and record all important observations in the subject's medical records with a water pen. These records include at least:

- Date of visit and the corresponding day or visit in the study plan (e.g., Screening, Day 1, etc.).
- General health status and condition noted for the subject, including any important medical findings. The severity, frequency, duration, and outcome of any reported AE and the investigator's assessment of the causal relationship between AE and IMP.
- Concomitant medications or dose changes.
- A general description of procedures that have been completed.
- Signatures or initials of all physicians who record in the medical records (course records).

In addition, any contact with the subject by telephone or other means, if clinically significant information can be provided, will also be recorded in medical records (course records) as described above.

The information on medical records (course records) and other source documents will be transcribed into the corresponding parts of the CRF in a timely manner.

Changes to information on the medical records (course records), CRFs, and other source documents will be signed and dated by the investigator or his/her designee on the day of change. If the reason for change is not clear, make a brief description of the change alongside.

## 11.3. Storage of Source Documents

Original documents include original observations and clinical study activities. Source documents include, but are not limited to, medical records (course records), computer printouts, screening logs, and recorded data from automated instruments.

All original documents of this study will be maintained by the investigator and made available for inspection by authorized personnel. Subjects may retain one of the signed ICF and the other one will be kept at the study site.

## 11.4. Study Data Records

All data obtained in this study will be the property of the sponsor.

Records must be maintained in accordance with current GCP guidelines. All necessary study documents, including subject records, original documents, CRFs, and IMP inventory must be documented.

In accordance with China GCP requirements, essential documents of clinical studies used to support drug registration applications should be retained for at least 5 years after the test drug has been approved for marketing. For clinical studies not designed to support drug registration applications, the essential documents should be preserved for at least 5 years after the termination of the clinical study.

Without written permission from the sponsor, the investigator should not process any records related to the study, which should be collected by the sponsor instead. The investigator should be responsible for maintaining appropriate and accurate hard disk copies of all observations and data generated during the course of this study. The investigator and the clinical trial institution should allow monitors, auditors, reviewers of the EC and inspectors of drug regulatory authority to have direct access to the source data and source documents related to the clinical study.

If the investigator leaves, withdraws from the study, or retires, the responsibility for maintaining records may be transited to another person. A notification of responsibility transition should be issued and agreed to by the sponsor.

## 11.5. Confidentiality

All information related to the health status of the subject obtained during the conduct of the study will be considered confidential. Written consent is required for the disclosure of any such information.

The investigator must ensure that each subject is kept secret. Subjects should not be identified by name on CRFs and other documents to be submitted to the sponsor or CRO.

In order to comply with the guidelines in government regulations and to ensure the safety of subjects, it is necessary for the sponsor and its representatives, CRO personnel, the local study review committee, or the National Medical Products Administration (NMPA) to review the subject's medical records when they are relevant to the study. The subject should only be identified by the unique number on the CRF, but his/her full name may be known by the drug regulatory authority or other authorized government or health officials, if necessary, and the person designated by the sponsor.

Documents not required to be submitted to the sponsor or to the CRO (e.g., ICFs) will be kept in absolute confidentiality by the investigator, unless they are required to be monitored by the sponsor and CRO, and audited by regulatory authorities. Documents containing subject's name cannot be taken away from the study site and the subject's identity should not be revealed in any publications related to the study.

## 11.6. Review by the Ethics Committee

The sponsor or its designated agent will be responsible for obtaining approval from the relevant regulatory authority in accordance with local country requirements.

Subjects are not allowed to participate in the study until approval is obtained. A copy of the approval letter (as required by the local country) will be provided to the investigator and the IRB/IEC/EC.

## 11.7. Protocol Amendments

In accordance with ICH-GCP E6 guidelines, the investigator should not deviate from or modify the protocol arbitrarily without the sponsor's permission and EC's approval for protocol amendments, unless such deviation or modification is necessary to eliminate a direct hazard to study subjects, or the changes are only related to the logistical or administrative aspects of the study (e.g., change of monitor or telephone number).

Any changes to the protocol must be treated as protocol amendments. Any possible amendments must be approved by the sponsor. Written protocol amendments must be

submitted to the relevant regulatory authorities and the responsible EC. The investigator must obtain EC approval of protocol amendments before implementing these changes, unless it is necessary to eliminate apparent direct hazards to the subject. In this case, the EC must be notified within 5 days of the protocol change.

All amendments to the protocol should obtain the written approval from the relevant regulatory authorities and the EC. As for amendments concerning administrative matters, only notification is required, but written approval is not needed. Once approved, protocol amendment will be distributed to all persons who receive the original protocol, with operational guidelines appended.

If, in the judgment of the local EC, the investigator, and/or sponsor, the protocol amendment alters the study design, process, and/or increases the potential risk to the subject, the currently approved written ICF will require similar modification. The modified ICF must also be reviewed and approved by the sponsor, relevant regulatory authorities, and IRB/IEC/EC. In this case, the subject's informed consent must be obtained again before continuing in the study.

## 11.8. Protocol Compliance and Protocol Deviations

The protocol must be thoroughly read and guidelines must be followed. There will be exceptions in emergency situations where direct intervention is required for subject protection, safety and health considerations based on the investigator's judgment or investigator assigned, appropriately trained, and professionally qualified sub-investigator's judgment.

For important protocol deviations due to an emergency, accident, or error, the investigator or his/her designee must contact medical monitor by telephone as early as possible. This allows for an early consensus as to whether the subject should continue the study. The investigator, sponsor, and medical monitor will document this decision.

## 11.9. Publication of Study Results

After completion of this study, the investigator may prepare for joint publication with the sponsor. The investigator cannot submit any data in this protocol for publication without consent of the sponsor.

## 11.10. Clinical Study Report

The final clinical study report will be prepared in accordance with the NMPA guidelines for the structure and content of clinical study reports. A final clinical study report will be prepared regardless of whether the study is completed or terminated early. The sponsor will provide each investigator with a copy of the final report for archiving.

## 11.11. Insurance, Indemnity, and Compensation

The sponsor is responsible for providing an appropriate clinical study insurance contract. Deviations from the protocol, in particular noncompliance with the planned dose instructions in the protocol, other modes of administration, other indications, and extended treatment duration, are not allowed and will not be covered by the subject's statutory insurance plan.

## 11.12. Study Termination

The sponsor may terminate the study. The study may also be terminated prematurely at any time, if agreed by the investigator and the sponsor, in the best interest of the subject and out of reasonable medical or ethical considerations. During the termination of the study, the sponsor, the CRO and the investigator should ensure that adequate consideration is given to the protection of the subjects' interests.

When the clinical study is prematurely terminated or suspended, the investigator should promptly notify the subject and give the subject appropriate treatment and follow-up.

## 11.13. Document Management at Study Site

Essential documents for drug clinical study refer to documents that assess the conduct of a clinical study and the quality of study data, which are used to demonstrate that investigators, the sponsor, and monitors have complied with GCP and relevant regulatory requirements for drug clinical study during this clinical study.

It is the responsibility of the investigator to maintain and preserve the essential documents for clinical study in accordance with the Guidelines for Preservation of Essential Documents for Drug Clinical Studies.

## 12. References

[1]. Rheumatology Branch of Chinese Medical Association, et al. 2020 Chinese guidelines for the diagnosis and treatment of systemic lupus erythematosus, Chinese Journal of Internal Medicine, March 2020, Vol.59, No.3, 172-185.

[2]. Jimenez S, Cervera R, et al. The epidemiology of systemic lupus erythematosus. Clin Rev Allergy & Immunology 2003;25:3-11.

[3]. Zeng QY, Chen R, Darmawan J, et al. Rheumatic diseases in China. Arthritis Res Ther, 2008, 10: R17-R27.

[4]. Aringer M, Costenbader K, Daikh D, et al. 2019 European League Against Rheumatism / American College of Rheumatology classification criteria for systemic lupus erythematosus. Ann Rheum Dis, 2019, 78(9): 1151-1159.

[5]. Tektonidou MG, Lewandowski LB, Hu J, et al. Survival in adults and children with systemic lupus erythematosus: a systematic review and Bayesian meta-analysis of studies from 1950 to 2016. Ann Rheum Dis, 2017, 76(12): 2009-2016.

[6]. Tsokos GC. Systemic lupus erythematosus. N Engl J Med. 2011;365:2110-2121.

[7]. Singh RR, Yen EY. SLE mortality remains disproportionately high, despite improvements over the last decade. Lupus. 2018;27(10):1577. Epub 2018 Jul 17.

[8]. Levy R A, Gonzalez-Rivera T, Khamashta M, et al. 10 Years of belimumab experience: What have we learnt?. Lupus, 2021 Oct;30(11):1705-1721.

[9]. Morand E F, Furie R, Tanaka Y, et al. Trial of anifrolumab in active systemic lupus erythematosus. New England Journal of Medicine, 2020, 382(3): 211-221.

[10]. Lipsky PE. Systemic lupus erythematosus: an autoimmune disease of B cell hyperactivity. Nature Immunology. 2001;2(9):764-766.

[11]. Hutloff A, Buchner K, Reiter K, et al. Involvement of inducible costimulatory in the exaggerated memory B cell and plasma cell generation in systemic lupus erythematosus. Arthritis Rheum. 2004;50(10):3211-3220.

[12]. Jacobi AM, Mei H, Hoyer BF, Mumtaz IM, Thiele K, Radbruch A, Burmester GR, Hiepe F, Dorner T. HLA-DRhigh/CD27high plasmablasts indicate active disease in patients with systemic lupus erythematosus. Ann Rheum Dis. 2010;69(1):305-308

[13]. Suzanne Cole, et al. Integrative analysis reveals CD38 as a therapeutic target for plasma cell-rich pre-disease and established rheumatoid arthritis and systemic lupus erythematosus. Arthritis Research & Therapy (2018) 20:85.

[14]. Falk Hiepe, Andreas Radbruch. Plasma cells as an innovative target in autoimmune disease with renal manifestations. Nat Rev Nephrol. 2016 Apr;12(4):232-240.

[15]. van de Donk, N.W., et al., Monoclonal antibodies targeting CD38 in hematological malignancies and beyond. Immunol Rev. 2016. 270(1): 95-112

[16]. Amici, S.A.; Young, N.A.; Narvaez-Miranda, J.; Jablonski, K.A.; Arcos, J.; Rosas, L.; Papenfuss, T.L.; Torrelles, J.B.; Jarjour, W.N.; Guerau-De-Arellano, M. CD38 Is Robustly Induced in Human Macrophages and Monocytes in Inflammatory Conditions. Front. Immunol. 2018, 9, 1593.

[17]. Lennard Ostendorf, et al. Targeting CD38 with Daratumumab in Refractory Systemic Lupus Erythematosus. N Engl J Med. 2020 Sep 17;383(12):1149-1155.

[18]. Expert group of Chinese Systemic Lupus Erythematosus Treatment and Research Group. Expert Consensus on the Rational Use of Glucocorticoids in Patients with Systemic Lupus Erythematosus [J]. Chinese Journal of Internal Medicine, 2014, 053(006):502-504.

## 13. Appendixes

### Appendix 1 2019 EULAR/ACR Classification Criteria for SLE

| 2019 EULAR/ACR Classification Criteria for SLE | | | |
|---|---|---|---|
| **Clinical Domain** | Weight | **Clinical Domain** | Weight |
| **1. Constitutional** | | **6. Hematologic** | |
| Fever ≥ 38.3°C | 2 | Leukopenia (< 4 × $10^9$/L) | 3 |
| **2. Mucocutaneous** | | Thrombocytopenia (< 100 × $10^9$/L) | 4 |
| Non-scarring alopecia | 2 | Autoimmune hemolysis | 4 |
| Oral ulcers | 2 | **7. Renal** | |
| Subacute cutaneous OR discoid lupus | 4 | Proteinuria > 0.5 g/24 h | 4 |
| Acute cutaneous lupus | 6 | Renal biopsy Class II or V lupus nephritis | 8 |
| **3. Arthritis** | | Renal biopsy Class III or IV lupus nephritis | 10 |
| ≥ 2 joints with synovitis/≥ 2 tender joints + morning stiffness lasting ≥ 30 minutes | 6 | **Immunology Domain** | Weight |
| | | **1. Antiphospholipid antibodies** | |
| **4. Neuropsychiatric** | | Anti-cardiolipin antibodies IgG > 40 GPL units OR anti-β2-GP1 antibodies IgG > 40 units OR lupus anticoagulant positive | 2 |
| Delirium | 2 | | |
| Psychosis | 3 | | |
| Seizure | 5 | **2. Complement proteins** | |
| **5. Serosal** | | Low C3 OR low C4 | 3 |
| Pleural or pericardial effusion | 5 | Low C3 AND Low C4 | 4 |
| Acute pericarditis | 6 | **3. Highly specific antibodies** | |
| | | Anti-ds-DNA antibody positive | 6 |
| | | Anti-Sm antibody positive | 6 |

Note: (1) The included patients must meet the criterion of ANA positivity (Hep2 immunofluorescence method ≥ 1:80); (2) In each domain, only the score of the highest weighted criterion is counted towards the total score; (3) For each criterion, causes such as infection, neoplasm malignant, and drug use should be excluded; (4) At least one clinical criterion must be met; (5) Both past and present conditions can be scored; (6) Patients with a total score of ≥ 10 points from the highest weighted criteria in each domain can be classified as having SLE.

Source: [1] Aringer M, et al. 2019 European League Against Rheumatism/American College of Rheumatology classification criteria for systemic lupus erythematosus Ann Rheum Dis 2019;78:1151-1159. [2] Zeng Xiaofeng, Li Mengtao, Tian Xinping. Systemic Lupus Erythematosus in China: A National Report of 2020. P192

## Appendix 2 SELENA-SLEDAI Score

### SELENA-SLEDAI Instrument Score

Record the patient's disease activity score for the current or past 10 days

| Weight | Descriptor | Definition |
|---|---|---|
| 8 | Seizure | Recent onset (last 10 days). Exclude metabolic, infectious or drug cause, or seizure due to past irreversible CNS damage. |
| 8 | Psychosis | Altered ability to function in normal activity due to severe disturbance in the perception of reality. Include hallucinations; incoherence; marked loose associations; impoverished thought content; marked illogical thinking, bizarre, disorganized or catatonic behavior. Exclude uremia and drug causes. |
| 8 | Organic brain syndrome | Altered mental function with impaired orientation, memory or other intellectual function, with rapid onset and fluctuating clinical features. Include clouding of consciousness with reduced capacity to focus, and inability to sustain attention to environment, plus at least 2 of the following: perceptual disturbance, incoherent speech, insonmia or daytime drowsiness, or increased or decreased psychomotor activity. Exclude metabolic, infectious or drug causes. |
| 8 | Visual disturbance | Retinal and eye changes of SLE. Include cytoid bodies, retinal hemorrhages, serous exudate orhemorrhages in the choroid, optic neuritis, scleritis or episcleritis. Exclude hypertension, infection or drug causes. |
| 8 | Cranial nerve disorder | New onset of sensory or motor neuropathy involving cranial nerves. Include vertigo due tolupus. |
| 8 | Lupus headache | Severe persistent headache: may be migramous, but must be nonresponsive to narcotic analgesia. |
| 8 | CVA | New onset of cerebrovascular accident(s). Exclude arteriosclerosis or hypertensive causes. |
| 8 | Vasculitis | Ulceration, gangrene, tender finger nodules, periungual infarction, splinter hemorrhages, or biopsy or angiogram proof of vasculitis. |
| 4 | Arthritis | More than 2 joints with pain and signs of inflammation (i.e., tenderness, swelling or effusion). |
| 4 | Myositis | Proximal muscle aching/weakness, associated with elevated creatine phosphokinase/aldolase or electromyogram changes or a biopsy showing myositis. |
| 4 | Urinary casts | Heme-granular or red blood cell casts. |
| 4 | Hematuria | >5 red blood cells/high power field. Exclude stone, infection or other cause. |
| 4 | Proteinuria | New onset or recent increase of more than 0.5 gm/24 hours. |
| 4 | Pyuria | >5 white blood cells/high power field. Exclude infection. |
| 2 | Alopecia | Ongoing abnormal, patchy or diffuse loss of hair due to active lupus. |
| 2 | Rash | Ongoing inflammatory lupus rash. |
| 2 | Mucosal ulcers | Ongoing oral or nasal ulcerations due to active lupus. |
| 2 | Pleurisy | Classic and severe pleuritic chest pain or pleural rub or effusion or new pleural thickening due to lupus. |

| | | |
|---|---|---|
| 2 | Pericarditis | Classic and severe pencardial pain or rub or effusion, or electrocardiogram confirmation. |
| 2 | Low complement | Decrease in CH50, C3 or C4 below the lower limit of normal for testing laboratory. |
| 2 | Increased DNA binding | >25% binding by Farr assay or above normal range for testing laboratory. |
| 1 | Fever | >38°C. Exclude infectious cause. |
| 1 | Thrombocytopenia | $< 100 \times 10^9$/L. |
| 1 | Leukopenia | $< 3 \times 10^9$/L. Exclude drug causes. |

Source: Petri M, Kim MY, Kalunian KC, et al. Combined oral contraceptives in women with systemic lupus erythematosus. N Engl J Med. 2005;353 (suppl): 2550 - 2558.

## Appendix 3 SLE Flare Scoring Index

| Mild or Moderate Flares | Severe Flare |
|---|---|
| … A change in SELENA-SLEDAI score of 3 points or more (but not exceeding 12 points)<br>… New/worsening:<br>Deep photo-sensitive discoid erythema<br>Cutaneous vasculitis and blisters<br>Nasopharyngeal ulcer<br>Pleurisy<br>Pericarditis<br>Arthritis<br>Pyrexia (due to SLE disease activity)<br>… Prednisone dose increased, but not exceeding 0.5 mg/kg/day<br>… NSAIDs or hydroxychloroquine added for active SLE<br>… An increase of 1.0 or more, but no more than 2.5, in PGA score | … SELENA-SLEDAI score change greater than 12<br>… New/worsening:<br>Central nervous system manifestations—systemic lupus erythematosus<br>Vasculitis<br>Nephritis<br>Myositis<br>Platelet count < 60,000<br>Hemolytic anaemia: hemoglobin < 70 g/L or a decrease in hemoglobin > 30 g/L<br>**Require:** Double the dose of prednisone, or increase prednisone to >0.5 mg/kg/day, or hospitalisation<br>… Prednisone increased to >0.5 mg/kg/day<br>… New cyclophosphamide, azathioprine, methotrexate, and mycophenolate used for active SLE<br>… Hospitalization due to active SLE<br>… Physician Global Assessment score (PGA score) increased by > 2.5 |

Source: [1] Petri M, Kim MY, Kalunian KC, et al. Combined oral contraceptives in women with systemic lupus erythematosus. N Engl J Med. 2005;353 (suppl): 2550 - 2558.

[2] Jill P Buyon, Michelle A Petri, Mimi Y Kim, et al. The effect of combined estrogen and progesterone hormone replacement therapy on disease activity in systemic lupus erythematosus: a randomized trial. Ann Intern Med. 2005 Jun 21;142(12 Pt 1):953-62.

## Appendix 4 BILAG-2004 Score

BILAG-2004 assesses the disease activity status of each organ system in the past 4 weeks, involving 9 organ systems: constitutional, mucocutaneous, neuropsyiatric, renal, hematologic, musculoskeletal, cardiorespiratory, gastrointestinal, and ocular.

**BILAG-2004 Assessment Criteria**

| Grade | Definition |
|---|---|
| A | Severe disease activity requiring any of the following treatment:<br>1. systemic high-dose oral corticosteroids (equivalent to prednisolone > 20 mg/day)<br>2. intravenous pulse glucocorticoids (equivalent to pulse methylprednisolone ≥ 500 mg)<br>3. systemic immunomodulators (include biologicals, immunoglobulins and plasmapheresis)<br>4. therapeutic high-dose anticoagulation in the presence of high-dose steroids or immunomodulators; e.g., warfarin with target INR 3-4 |
| B | Moderate disease activity requiring any of the following treatment:<br>1. systemic low-dose oral glucocorticoids (equivalent to prednisone ≤ 20 mg/day)<br>2. intramuscular or intra-articular or soft tissue glucocorticoids injection (equivalent to methylprednisolone < 500 mg)<br>3. topical glucocorticoids<br>4. topical immunomodulators<br>5. antimalarials or thalidomide or prasterone or acitretin<br>6. symptomatic therapy; e.g., NSAIDs for inflammatory arthritis |
| C | Mild |
| D | Inactive but previously affected |
| E | Inactive with no previous involvement |

Source: Chee-Seng Yee, Vernon Farewell, David A Isenberg, et al. The BILAG-2004 index is sensitive to change for assessment of SLE disease activity. Rheumatology 2009;48: 691-695

## BILAG-2004 Assessment Scale

Only record manifestations/items due to SLE disease activity, and assessment referes to manifestations occurring in the last 4 weeks (compared with the previous 4 weeks).

Scoring Criteria
- 0 Not present
- 1 Improving
- 2 Same
- 3 Worse
- 4 New
- or ND not done
- or Enter laboratory value at designated location
- or Yes/No
- *Yes/No Circle "Yes" or "No" to indicate whether the abnormal value is caused by SLE.

**Constitutional**

1. Pyrexia - documented >37.5°C ( )
2. Weight loss - unintentional >5% ( )
3. Lymphadenopathy/splenomegaly ( )
4. Anorexia ( )

**Mucocutaneous**

5. Skin eruption - severe ( )
6. Skin eruption - mild ( )
7. Angio-oedema - severe ( )
8. Angio-oedema - mild ( )
9. Mucosal ulceration - severe ( )
10. Mucosal ulceration - mild ( )
11. Panniculitis/Bullous lupus - severe ( )
12. Panniculitis/Bullous lupus - mild ( )
13. Major cutaneous vasculitis/thrombosis ( )
14. Digital infarcts or nodular vasculitis ( )
15. Alopecia - severe ( )
16. Alopecia - mild ( )
17. Periungual erythema/chilblains ( )
18. Splinter haemorrhage ( )

**Neuropsychiatric**

19. Aseptic meningitis ( )
20. Cerebral vasculitis ( )
21. Demyelinating syndrome ( )
22, Myelopathy ( )
23. Acute confusional state ( )
24. Psychosis ( )
25. Acute inflammatory demyelinating polyradiculoneuropathy ( )
26. Mononeuropathy (single/multiplex) ( )
27. Cranial neuropathy ( )

**Cardiopulmonary**

44. Myocarditis - mild ( )
45. Myocarditis/endocarditis + cardiac failure ( )
46. Arrhythmia ( )
47. New valvular dysfunction ( )
48. Pleurisy/pericarditis ( )
49. Cardiac tamponade ( )
50. Pleural effusion with dyspnoea ( )
51. Pulmonary haemorrhage/vasculitis ( )
52. Interstitial alveolitis/pneumonia ( )
53. Shrinking lung syndrome ( )
54. Aortitis ( )
55. Coronary vasculitis ( )

**Gastrointestinal**

56. Lupus peritonitis ( )
57. Abdominal serositis or ascites ( )
58. Lupus enteritis/colitis ( )
59. Malabsorption ( )
60. Protein-losing enteropathy ( )
61. Intestinal pseudo-obstruction ( )
62. Lupus hepatitis ( )
63. Acute lupus cholecystitis ( )
64. Acute lupus pancreatitis ( )

**Ophthalmic**

65. Orbital inflammation/myositis/proptosis ( )
66. Keratitis - severe ( )
67. Keratitis - mild ( )
68. Anterior uveitis ( )
69. Posterior uveitis/retinal vasculitis - severe ( )
70. Posterior uveitis/retinal vasculitis - mild ( )
71. Episcleritis ( )
72. Scleritis - severe ( )
73. Scleritis - mild ( )
74. Retinal/choroidal vaso-occlusive disease ( )
75. Isolated cotton-wool spots (cytoid bodies) ( )
76. Optic neuritis ( )
77. Anterior ischaemic optic neuropathy ( )

**Renal**

78. Systolic blood pressure (abnormal value > 140 mm Hg) value ( ) Yes/No*
79. Diastolic blood pressure (abnormal value > 90 mm Hg) value ( ) Yes/No*
80. Accelerated hypertension Yes/No ( )

| | |
|---|---|
| 28. Plexopathy ( )<br>29. Polyneuropathy ( )<br>30. Seizure disorder ( )<br>31. Status epilepticus ( )<br>32. Cerebrovascular disease (not due to vasculitis)<br>33. Cognitive dysfunction ( )<br>34. Movement disorder ( )<br>35. Autonomic disorder ( )<br>36. Cerebellar ataxia (isolated) ( )<br>37. Lupus headache - severe unremitting ( )<br>38. Headache from IC hypertension ( )<br>**Musculoskeletal**<br>39. Myositis severe ( )<br>40. Myositis - mild ( )<br>41. Arthritis (severe) ( )<br>42. Arthritis (moderate)/tendonitis/tenosynovitis ( )<br>43. Arthritis (mild)/arthralgia/myalgia ( ) | 81. Urine dipstick protein (+=1, ++=2, +++=3) value ( ) Yes/No*<br>82. UACR (mg/mmol) value ( ) Yes/No*<br>83. UPCR (mg/mmol) value ( ) Yes/No*<br>84. 24-hour urine protein (g) value ( ) Yes/No*<br>85. Nephrotic syndrome Yes/No ( )<br>86. Creatinine (plasma/serum: abnormal value >130 µmol/L) value ( ) Yes/No*<br>87. GFR (abnormal value < 80 mL/min/1.73 $m^2$) value ( ) Yes/No*<br>88. Active urinary sediment Yes/No ( )<br>89. Active nephritis Yes/No ( )<br>**Haematological**<br>90. Haemoglobin (abnormal value ≤ 10.9 g/dL) value ( ) Yes/No*<br>91. Total white cell count (abnormal value ≤ 3.9 × $10^9$/L) value ( ) Yes/No*<br>92. Neutrophils (abnormal value ≤ 1.9 × $10^9$/L) value ( ) Yes/No*<br>93. Lymphocytes (abnormal value ≤ 1.0 × $10^9$/L) value ( ) Yes/No*<br>94. Platelets (abnormal value ≤ 149 × $10^9$/L) value ( ) Yes/No*<br>95. TTP records 0, 1, 2, 3, 4.<br>96. Evidence of active hemolysis Yes/No ( )<br>97. Coomb's test positive (isolated) Yes/No ( ) |

Abbreviations: GFR=glomerular filtration rate; TTP=thrombotic thrombocytopenic purpura; UACR=urine albumin/creatinine ratio; UPCR=urine protein/creatinine ratio.

## Appendix 5 PGA Score

PGA is an index system in which the physician directly asks or observes the patient's overall condition in the past 2 weeks and then conducts a subjective score. For SLE patients undergoing PGA assessment, 0 points represent no activity, 1 point represents mild activity, 2.0 to 2.5 points represent moderate activity, and 3 points represent severe activity. For non-initial assessments, if the score increases by ≥ 0.3 compared to the last findings, the patient's condition is considered to have worsened.

**PGA score**

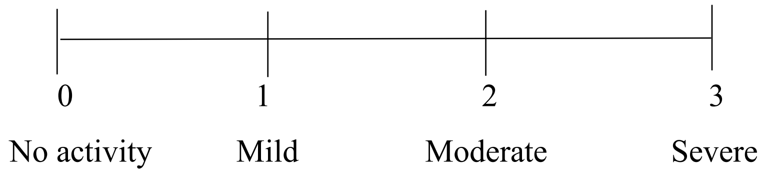


Note: PGA = Physician Global Assessment.

Source: [1] Petri M, Kim MY, Kalunian KC, et al. Combined oral contraceptives in women with systemic lupus erythematosus. N Engl J Med. 2005;353 (suppl): 2550 - 2558. [2] K M A C Luijten, J Tekstra, J W J Bijlsma, et al. The Systemic Lupus Erythematosus Responder Index (SRI); A new SLE disease activity assessment. Autoimmunity Reviews 11 (2012) 326-329

## Appendix 6 Contraceptive Measures, Definition of Women of Childbearing Potential, and Contraceptive Requirements

Male subjects with azoospermia (due to vasectomy or other underlying diseases) and their partners are not required to use contraception.

Female subjects are considered not of childbearing potential if:

1) Postmenopausal (postmenopausal status is defined as menopause for 12 months without other medical reasons. For women who are not using hormonal contraception or hormone replacement therapy, if follicle stimulating hormone [FSH] levels are within the postmenopausal range, their postmenopausal state can be confirmed);

or

2) Have undergone hysterectomy and/or bilateral oophorectomy, bilateral salpingectomy, or bilateral tubal ligation/occlusion surgery at least 6 weeks prior to screening;

or

3) Congenital or acquired infertility.

Female subjects of childbearing potential and male subjects with a female partner of childbearing potential must agree to use contraception. Female subjects and their partners must use one of the following highly effective methods of contraception from signing the informed consent form (ICF) until 4 months after the last dose of the IMP. Male subjects and their partners must use contraception from signing the ICF until 6 months after the last dose of the IMP. When used consistently and correctly, methods that can achieve a failure rate of less than 1% are considered highly effective methods of contraception and include:

- Combined (estrogen and progestogen containing) hormonal contraception associated with inhibition of ovulation that is oral, intravaginal or transdermal;
- Progesterone contraceptive associated with the inhibition of ovulation that is oral, injectable, implantable;
- Intra-uterine device (IUD);
- Intrauterine hormone-releasing system (IUS);
- Bilateral tubal ligation;
- A vasectomized partner is considered a highly effective method of contraception provided that the partner is the sole sexual partner of the female subject of childbearing potential and has undergone a successful vasectomy (medically assessed);

- Sexual abstinence is considered a highly effective method only if defined as refraining from heterosexual intercourse during the entire period of risk associated with the IMPs. The reliability of sexual abstinence needs to be evaluated in relation to the duration of the study and the preferred and usual lifestyle of the subject. ）

In addition, the subjects should be informed that the investigator should be notified immediately if they discontinue the contraceptive methods or if they or their partner have a suspected or confirmed pregnancy.
